# Marine fish ranges are expanding and shifting deeper across the European continental shelf

**DOI:** 10.1038/s41467-026-74371-8

**Published:** 2026-07-15

**Authors:** Arnaud Auber, Martin P. Marzloff, David Mouillot, Kari E. Ellingsen, Kirsteen M. MacKenzie, Fabien Leprieur, Patrica Belloeil, Matthew J. McLean, Daniel G. Boyce, Adrián A. González Ortiz, Rita Vasconcelos, David M. Keith, Eva Maire, Aurore Receveur, Bastien Mourguiart, Camille Albouy

**Affiliations:** 1 IFREMER, HMMN, Laboratoire Ressources Halieutiques, Boulogne-sur-Mer, France; 2 IFREMER, DYNECO, Plouzané, France; 3 MARBEC, Univ Montpellier, CNRS, Ifremer, IRD, Montpellier, France; 4 Norwegian Institute for Nature Research (NINA); Fram Centre, Tromsø, Norway; 5 Department of Biology and Marine Biology, Center for Marine Science, University of North Carolina Wilmington, Wilmington, NC USA; 6 Wild Ocean Research, Cow Bay, NS Canada; 7 Portuguese Institute for Sea and Atmosphere, Lisbon, Portugal; 8 Fisheries and Oceans Canada, Bedford Institute of Oceanography, Halifax, NS Canada; 9 Lancaster Environment Centre, Lancaster University, Lancaster, UK; 10 Fondation Pour la Recherche sur la Biodiversité, CESAB, Montpellier, France; 11 Unit of Land Change Science, Swiss Federal Research Institute WSL, Birmensdorf, Switzerland; 12 Department of Environmental Systems Science, ETH Zürich, Ecosystems and Landscape Evolution, Institute of Terrestrial Ecosystems, Zürich, Switzerland

**Keywords:** Climate-change ecology, Biogeography, Marine biology

## Abstract

Understanding how climate change and exploitation affect marine species distribution is key for biodiversity conservation. While warming is typically expected to drive species poleward and into deeper waters, unexpected patterns such as equatorward shifts and shallowing also occur. Fishing pressure may further contract species’ ranges. Yet, the mechanisms underpinning these 3-dimensional range shifts remain largely unresolved. Using 92,961 bottom trawl samples across the European continental shelf, covering 607 fish species over four decades, we show that deepening is predominant, with 60% of populations experiencing an increase in their average depth. Contrary to expectations, latitudinal shifts are balanced, with 49% of populations shifting poleward and 51% equatorward. Here, we quantify how population relocations are driven by the interplay between environmental factors and species traits as species track their optimal ecological niches. We also highlight that the recent reduction in fishing pressure has likely facilitated spatial expansions and increases in populations abundance.

## Abstract (web summary)

As climate warms, marine species are expected to shift poleward or to deeper, cooler waters. This study shows that marine fish populations may move to deeper habitats, while latitudinal changes are balanced between poleward and equatorward shifts.

## Introduction

Species range shifts toward higher latitudes, greater elevations, or deeper depths are among the most widely recognized and anticipated biodiversity responses to climate change^1–3^. Nevertheless, a growing body of evidence suggests that species responses to climate change are, however, far more complex and multifaceted^3–5^, leading to unexpected species range shifts^3,6^. For instance, intricate yet poorly understood interactions among multiple factors—such as regional variations in climate change^7^, biotic interactions^8^, or size-, zone-, and depth-specific harvesting^9,10^—can result in unexpected or even absent range shifts^3,6^. Counterintuitively, human-induced pressures, particularly those related to fisheries, are often overlooked, even in historically exploited regions like the Northeast Atlantic (NeA)^11^ and the Mediterranean Sea (MedS)^12^. In these areas, research has primarily concentrated on the effects of rapid ocean warming^13,14^, whereas the few studies addressing commercial species have focused only on a narrow range of taxa^15^. Accumulated evidence reveals substantial reorganizations in fish communities, in both taxonomic and functional composition^16,17^, as rapid warming induced both overall tropicalisation^18^ and an increase in commercial fish abundances^19^ and biodiversity metrics through poleward shifts^20^. Depth shifts have also been detected for a limited number of species and in specific regions^21–23^ (see Supplementary Data 1 and 2 for an extensive literature review of latitudinal and bathymetric shifts). Moreover, substantial changes in the spatial distribution of commercial fish stocks in the NeA over recent decades^15^ create novel challenges for fisheries stock assessment and management. To support effective biodiversity conservation and resource management^22^, there is consequently an urgent need to understand how marine fish are shifting across European seas and how the interaction between climate, fishing, and other factors are influencing these redistributions.

**Fig. 1:**
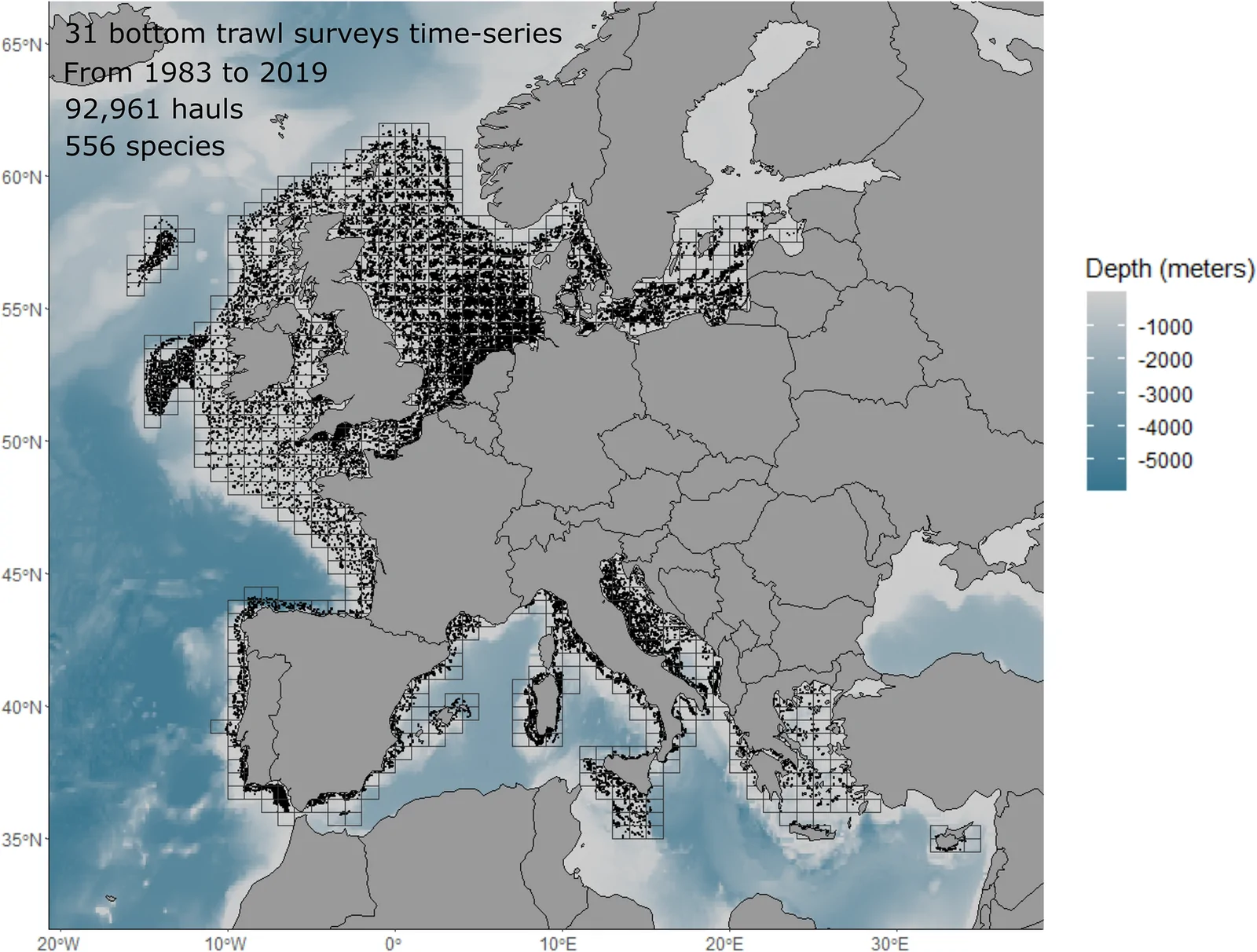
Spatial distribution of the 92,961 haul stations conducted during 21 bottom trawl programs in the Northeast Atlantic (NeA) and Mediterranean Sea (MedS) from 1983 to 2019. Each dot represents a haul and grid cells corresponds to 1° longitude by 0.5° latitude spatial units defined by the International Council for the Exploration of the Sea (ICES). To cover the entire study area with a consistent spatial resolution, we expanded the Atlantic ICES grid to the MedS. Sampling was conducted during daylight hours at an average speed of 3.4 ± 0.5 knots and an average duration of 34.4 ± 13.2 min.

Here, we take advantage of standardized abundance data collected from 21 long-term scientific bottom trawl programs (representing a total of 31 survey time-series, each corresponding to a specific program conducted during a given season quarter) to examine depth and latitudinal range shifts for 556 fish species across the NeA and MedS regions between 1983 and 2019 (Fig. 1). We assess the extent to which species have moved deeper or northward, both at the survey time-series scale and European scale. Then, we develop a niche-tracking framework and model the relationships between population shifts (where “population” is a species in a given survey time-series), species traits, environmental safety margins (i.e., the difference between environmental condition values and species’ lower and upper environmental tolerance limits), and fishing pressure to gain deeper insight into the causes of species range shifts. The replicated nature of the multiple survey time-series and their long-term monitoring offers a unique framework to evaluate the extent of species range shifts in relation to theoretical expectations.

## Results

### Bathymetric and latitudinal range shifts

**Fig. 2:**
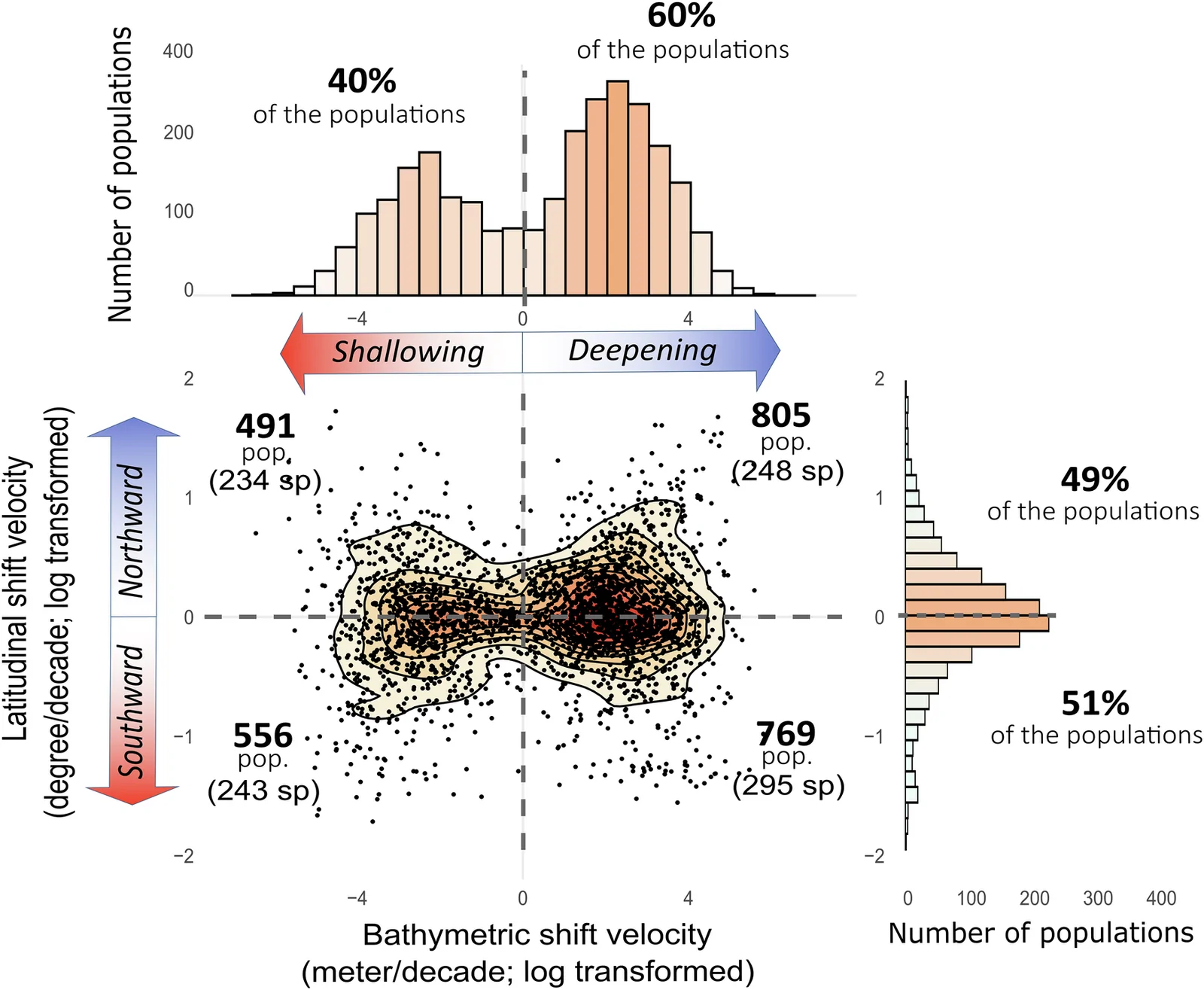
Depth and latitudinal shift velocities of European marine fish populations. Shift velocities of all populations (i.e., exhibiting significant and non-significant temporal change in depth and latitudinal position of their centroid of geographic distribution are displayed and used for calculations). This analysis is conducted at the survey time-series scale, with each dot representing a population (i.e., a species in a survey time-series). Orange gradients represent the density populations along the bathymetric and latitudinal velocity axes. In the figure, “pop” refers to population. The density polygons were built using the stat_density_2d and geom_density_2d from the ggplot2 R package^73^.

**Fig. 3:**
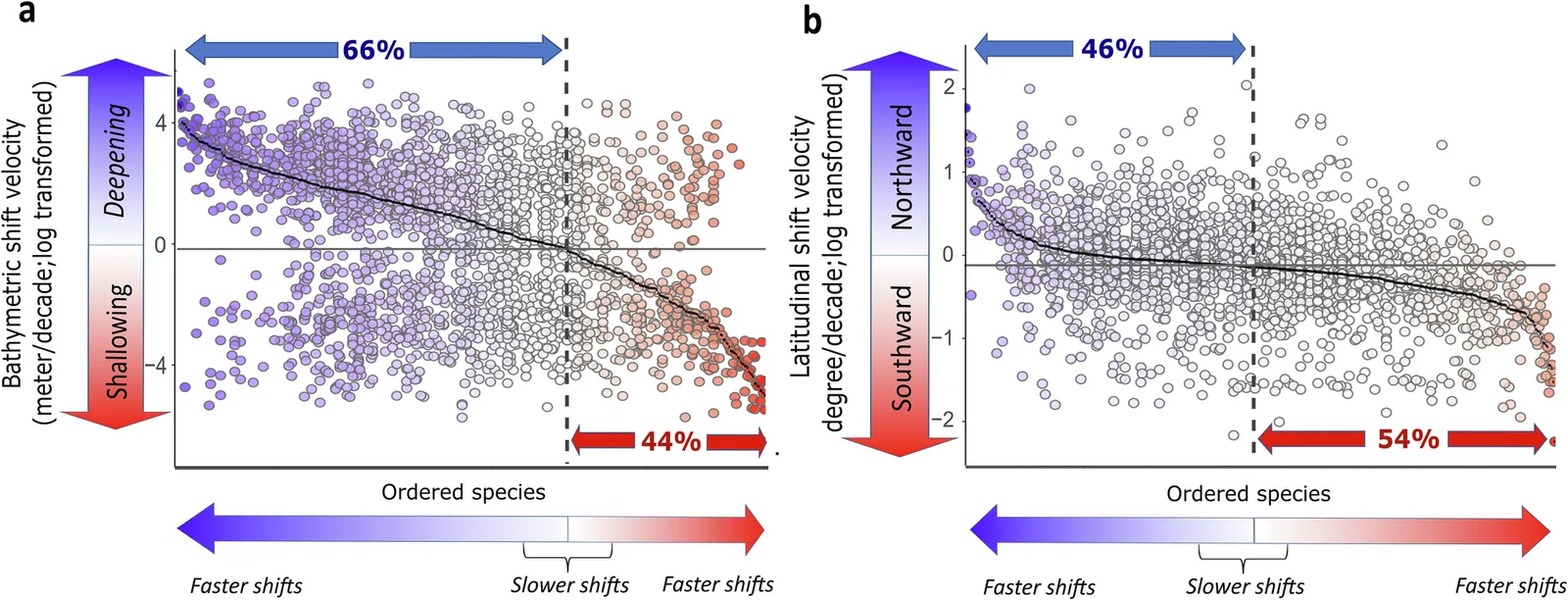
Species ranking based on their median shift velocity across all survey time-series. **a** Species ranking based on their median depth shift velocity. **b** Species ranking based on their median latitudinal shift velocity. All species are shown, whether they exhibit a significant or a non-significant temporal change in depth and latitudinal position of their centroid of geographic distribution. The blue and red color intensities correspond to the population’ shift velocity, where each dot represents a population (i.e., a species in a survey time-series). This analysis is conducted at the survey time-series scale. A black dashed line represents the median value of a given species (based on all survey time-series where that species was found).

To quantify the speed at which species shift their ranges, we calculated their centroid of geographic distribution (CGD) (a weighted average of their distribution across depth and latitude), adjusted for sampling effort, along both the bathymetric and latitudinal gradients within each survey time-series and for each year, and then determined how this center changed over time (“Methods”). We found a marked deepening tendency for most fish species in European waters. Approximately 60% of fish populations showed an increase in the mean depth of their centroid at a mean speed of 18 ± 27 m.decade^−1^ (“Methods”; Fig. 2 and Supplementary Table 1), representing about 12% of the starting depth per decade, and 9% of the starting depth range (Supplementary Tables 2 and 3, respectively; see also Supplementary Figs. 1–5 for comparison across survey time-series). When considering only species exhibiting significant increases in the mean depth, the median relative velocity was about 23% of the starting depth per decade, and 16% of the starting depth range (“Methods”). The predominant deepening trend (i.e., 60%) became even more pronounced when considering only species exhibiting statistically significant depth and latitudinal shifts (in order to remove potential noise that might be hovering around low shift values), with approximately 76% of these moving deeper at a mean speed of 24 ± 29 m.decade^−1^ (Supplementary Fig. 6a). Because catchability may vary among species^24^, and as all abundance data were obtained from bottom trawl programs, pelagic species were excluded from the analyses to evaluate whether the percentage of populations shifting deeper was affected. The previously observed prevalence remained virtually unchanged, decreasing only slightly from 60.1 to 59.5% after excluding pelagic species. When considering only significant bathymetric shifts, this proportion also remained stable, changing from 76 to 75.5%. According to the median depth shift velocity at the species level (i.e., across all populations for a given species), 66% of species moved to deeper waters (Fig. 3a). This percentage was even higher (i.e., 76%) when considering only species with significant shifts (Supplementary Fig. 7a). We also detected the dominance of deepening over shallowing when analyses were performed at the European scale, both in the NeA and MedS, and in almost all seasonal quarters (Supplementary Figs. 8 and 9).

Conversely, no clear trends of polewards range shifts emerged, with similar percentages for both northward and southward shifts. Specifically, 49% of fish populations showed a northward displacement (63 ± 87 km dec^−1^), while the remaining 51% shifted southward (70 ± 101 km dec^−1^; Fig. 2 and Supplementary Table 4) in the centroid of their geographic distribution. Similar percentages were observed when considering only species with significant latitudinal shifts, with 52 and 48%, respectively showed a northward 103 ± 101 km.dec^−1^) or southward displacement of their centroid (94 ± 106 km.dec^−1^; Supplementary Fig. 6 and Supplementary Table 4). The percentage of populations shifting northward remained virtually unchanged when pelagic species were excluded, decreasing only slightly from 49.4 to 48.8%. When considering only significant latitudinal shifts, this proportion also showed little variation, from 51.9 to 50.7%. Because the latitudinal range of each population may represent only a fraction of its overall global distribution (median = 33%; Supplementary Fig. 10), our ability to detect latitudinal shifts may be limited. To assess this limitation, we conducted a complementary analysis to determine whether the observed balance between northward and southward shifts remained consistent across species pools stratified by their relative latitudinal range—that is, the proportion of a species’ global latitudinal distribution encompassed by the population’s range within a given survey time-series. This analysis confirmed that the balanced pattern of northward and southward shifts persisted even when excluding populations whose survey-scale latitudinal range was small relative to their global scale range (Supplementary Fig. 11). The median latitudinal shift velocities confirmed the absence of a clear poleward direction in latitudinal shifts, as 46 and 54% of species exhibited significant northward and southward shifts, respectively (Fig. 3b). These percentages were even more balanced (i.e., 47 and 53%) when considering only species with significant shifts (Supplementary Fig. 7b). In agreement with these results at the survey time-series scale, similar percentages of northward and southward shifts were also quantified at larger scale when aggregating all survey time-series (Supplementary Figs. 8 and 9). We note, however, that the MedS was predominantly characterized by southward shifts (Supplementary Figs. 8 and 9).

### Spatial expansions and abundance trends

**Fig. 4:**
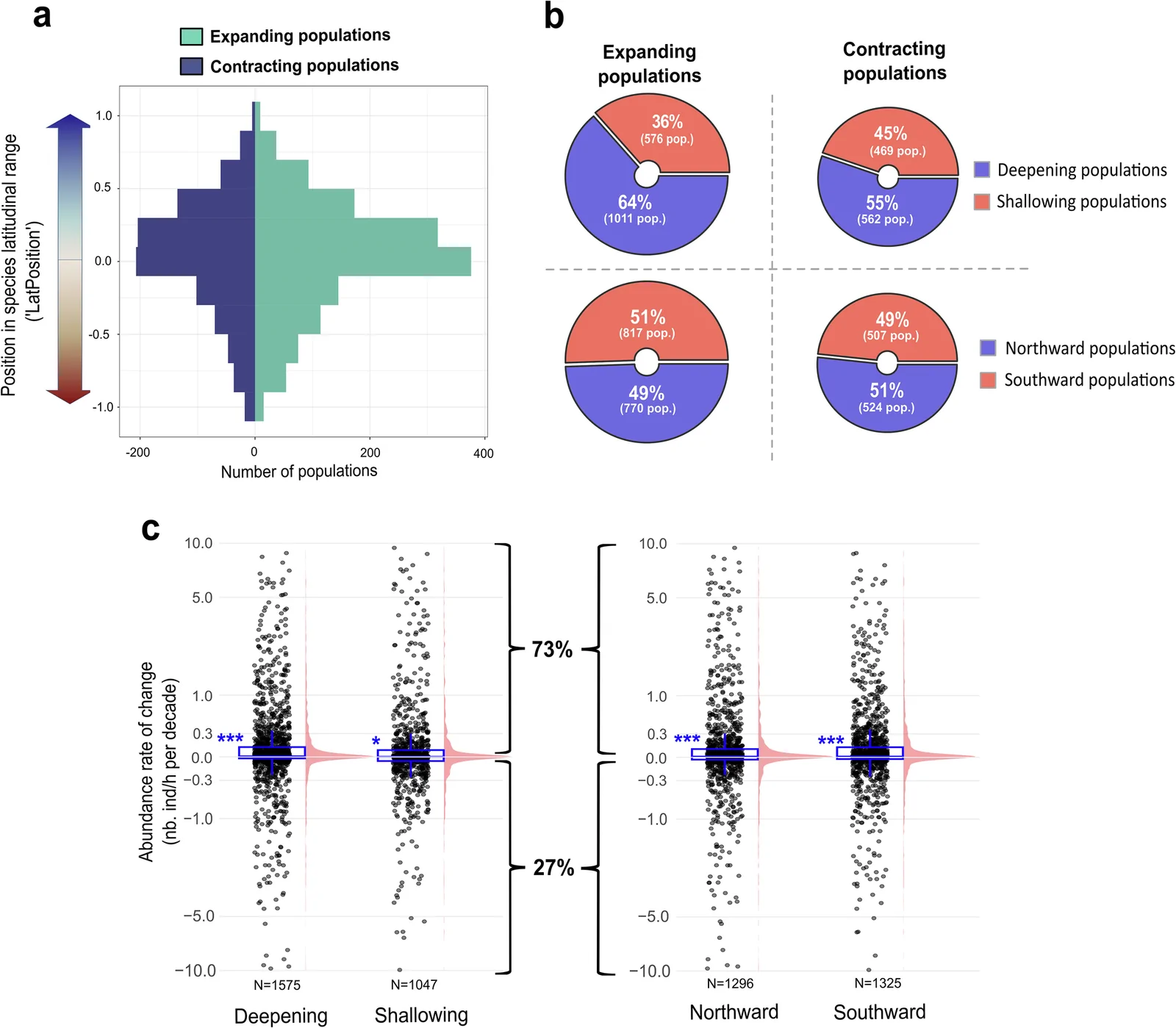
Range expansion/contraction of populations along the latitudinal gradient and rate of change in population abundances. **a** Number of populations exhibiting spatial expansion or contraction within each survey time-series, depending on the position of the survey within the species range (−1, 0, and 1 represent the southern limit, center, and northern limit, respectively). **b** Percentages of the populations exhibiting deepening vs. shallowing and northward vs. southward shifts within expanding species and contracting species. **c** Rate of change in population abundances for populations shifting deeper (*p* < 2.2e-16), shallower (*p* = 0.011), northward (*p* < 2.3e-08), or southward (*p* < 4.5e-11). Each sample represents a species within a survey, characterized by its bathymetric (or latitudinal) shift trend and its rate of change in abundance (measured as the number of individuals per hour per decade). The box plots show the median (centre line), the interquartile range (box bounds, from the 25th to the 75th percentile: IQR), whiskers extending to the minimum and maximum (i.e., within 1.5 × IQR). All these analyses are conducted at the spatial scale of survey time-series. Stars denote the level of significance based on two-sided Wilcoxon signed-rank tests used to compare the median rate of abundance change against zero (i.e., no temporal trend): *: 0.01 <*p* < 0.05; **: 0.001 <*p* < 0.01; ***: *p* < 0.001.

Interestingly, we found that most fish populations (i.e., 61%) expanded their spatial extent, regardless of their position within their latitudinal distribution (Fig. 4a), supporting the absence of a consistent trend towards either northward or southward dominance of movement. This absence of a clear trend along the latitudinal gradient was also reflected in the rate of change in spatial extent, which was not consistently positive in the northern parts of species’ latitudinal ranges nor negative in the southern parts, contrary to the expected northward shift hypothesis associated with global warming (Supplementary Fig. 12). More specifically, the spatial expansion of populations was primarily associated with geographic shifts toward deeper rather than shallower areas, whereas northward and southward expansions were proportionally similar (Fig. 4b). Finally, we observed that the majority (i.e., 73%) of populations, whatever the direction of change (i.e., deepening, shallowing, northward, or southward) were characterized by abundance increase (Fig. 4c). This percentage remained very consistent, changing only from 73 to 68% when non-commercial species were excluded, and from 73 to 72% when commercial species were removed from analyses (Supplementary Fig. 13). This majority (i.e., 73%) was even higher when considering only populations associated with a significant depth or latitudinal shift (75 and 78%, respectively; Supplementary Fig. 14).

### Niche tracking

To understand whether fish are shifting their distributions to escape unsuitable environmental conditions or to find more suitable ones, we developed a niche-tracking framework based on two metrics. The “experienced environment” reflects the conditions where the species was most abundant each year, and the “reference environment” is based on conditions in areas where the species was initially found. By analyzing how these metrics changed over time, we assessed whether fish were tracking favorable habitats or responding differently to environmental changes (“Methods”). We also developed an original niche-tracking framework (“Methods”) to quantify how species redistribute to track long-term environmental changes (Supplementary Fig. 15 for temporal trends of environmental parameters). During recent decades, the NeA and MedS generally experienced an increase in sea bottom temperature, chlorophyll-a, mixed layer depth (MLD) (i.e., the depth in the upper layer where physical properties are relatively uniform) and pH (70, 76, 89, and 99% of fish populations, respectively), a mixture of increasing and decreasing bottom salinities (55 vs 45%, respectively), and a predominant decrease (91%) in bottom oxygen concentrations (Supplementary Figs. 15–17). We observed that most populations experienced less environmental change—such as variations in sea bottom temperature, chlorophyll-a concentration (Chl-a), MLD, and salinity—primarily as a result of their spatial relocation rather than remaining stationary (Supplementary Figs. 16 and 17). These findings therefore support the hypothesis that most fish populations responded to environmental changes by tracking their ecological niches.

### The role of environment, fishing, safety margins, and traits

**Fig. 5:**
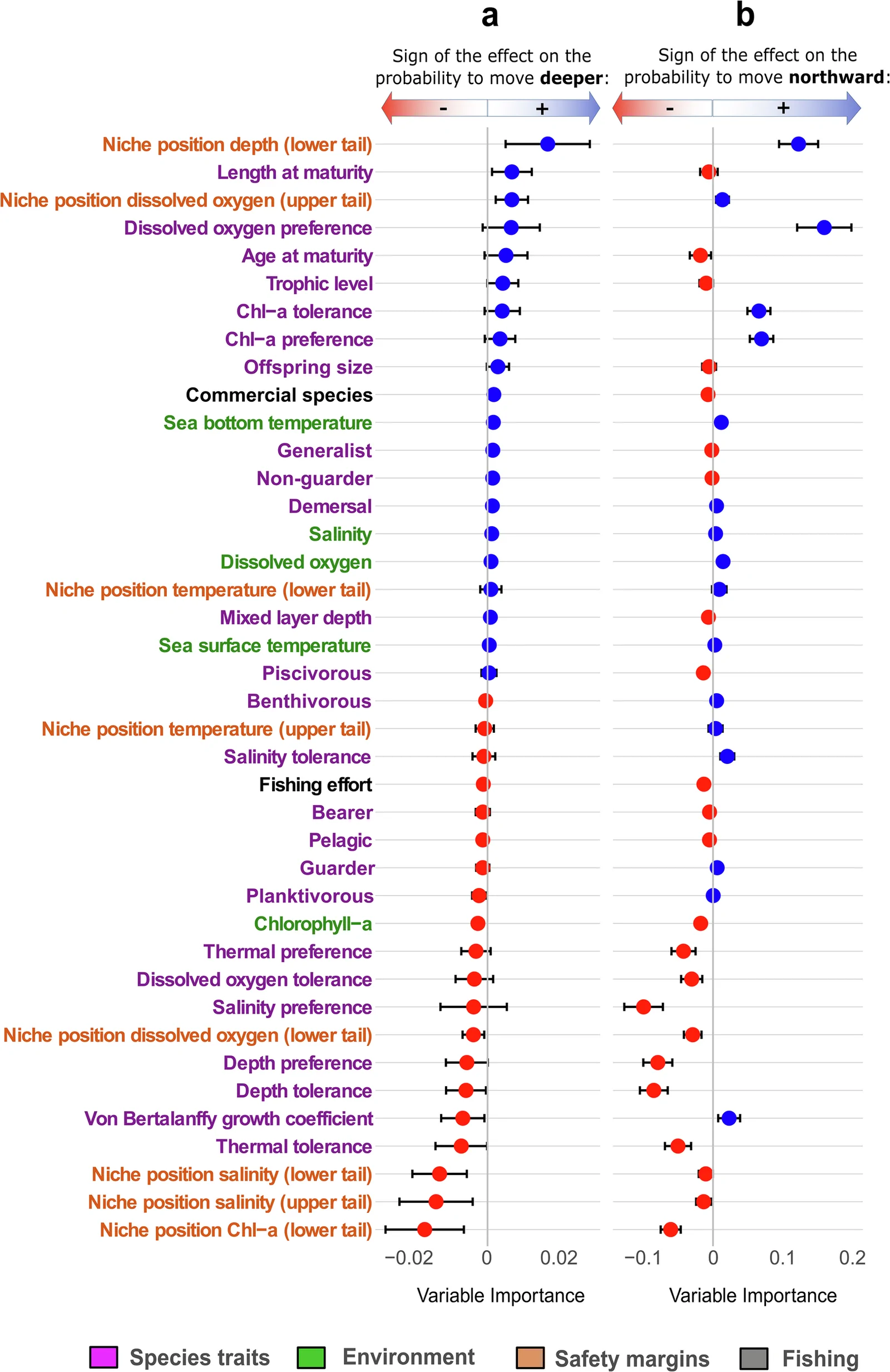
Influence of safety margins, species traits, fishing, and environmental changes on the probability of a species shifting deeper and northward. **a** Extreme Gradient Boosting model results for deepening shifts. **b** Extreme Gradient Boosting model results for northward shifts. Horizontal bars represent standard deviations of variable importance derived from 5000 null model runs (see “Methods”). For the bathymetric and latitudinal analyses, the total number of samples (i.e., a species within a survey) was 386 and 509, respectively. Each sample is characterized by the temporal trend in bathymetric (or latitudinal) shift, as well as by the set of model predictors (i.e., environmental trends, fishing effort trends, species traits, and safety margins). Yearly means of environmental drivers are considered. This analysis was conducted at the spatial scale of survey time-series. When predictors relative to “Niche position” increase, it indicates that the species experienced environmental conditions near the limits of its niche, with “lower tail” and “upper tail” corresponding to the lower and upper part of their niche, respectively.

Using an extreme gradient boosting (XGBoost)-based machine learning method, we revealed that a combination of species traits, environmental safety margins, and environmental changes influenced both depth and latitudinal shifts (Fig. 5). High trophic level, slow-growing, long-lived species, which prefer colder and shallower waters, were more likely to shift their distribution towards deeper areas than fast-growing species. This finding aligns with previous hypotheses suggesting that long-lived species, which are generally larger and more mobile, can access a broader range of environmental conditions^1,25^. Furthermore, species with narrower bathymetric, oxygen, and thermal niches were more inclined to move deeper than species with broader environmental niches (Fig. 5a, Supplementary Fig. 18, and Supplementary Table 5), which supports above results related to the niche-tracking hypothesis. Salinity generally increases with depth both in NeA and MedS (Supplementary Fig. 19), and species experiencing salinity close to their upper limit were less likely to move deeper (Fig. 5a). We also noted that decreases in oxygen levels may hinder species from moving deeper, especially when species experienced oxygen levels close to their lower limit tolerance (Fig. 5a and Supplementary Fig. 18). Species were more likely to shift to deeper areas when experiencing decreasing Chl-a concentrations (Fig. 5a). Similarly, increases in sea surface (and bottom) temperature and bottom salinity enhanced the likelihood of species migrating to greater depths (Fig. 5a). Similarly, environmental safety margins emerged as good predictors of depth shifts, with species living close to their upper or lower environmental tolerance limits being more likely to shift. Combining environmental niches with species traits thus offers greater insight than considering species traits alone to explain species range shifts^1,26^. Counterintuitively, the thermal safety margin had a very low impact, with species experiencing temperatures near their upper limit less likely to move deeper (Fig. 5a). We also observed that sea bottom temperature was not identified as one of the most influential drivers, highlighting that species responses are considerably more complex than being solely temperature-driven. Species’ commercial status and fishing effort had a limited effect on the probability of moving deeper (Fig. 5a) or northward (Fig. 5b). These findings were further corroborated by complementary analyses incorporating spatially resolved fishing effort data, which revealed no clear differences in species redistribution toward greater/lower depths and northern/southern latitudes (Supplementary Figs. 20 and 21). However, we note that when species with both significant and non-significant shifts are considered, fishing appears to have a marginal impact, with a general trend of species shifting their distributions toward greater depths (Supplementary Fig. 22), consistent with the negative relationship between fishing effort and depth (Supplementary Fig. 23). Finally, the influence of species’ traits on latitudinal shifts mirrored the patterns observed with depth shifts (Fig. 5a, b), underscoring the consistent role of species traits and safety margins in shaping species’ responses to environmental changes.

## Discussion

Using an extensive dataset from 31 bottom trawl survey time-series conducted across European continental shelf waters over the last four decades, we identify a strong discrepancy between depth and latitudinal shifts in marine fish species. Species tended to move deeper, and while no consistent patterns of latitudinal shifts were observed in the NeA, evidence of southward shifts was found in the MedS. Although both deeper and poleward shifts are recognized responses to ocean warming, the pronounced prevalence of deepening compared to the absence of a latitudinal trend is striking. These findings challenge the assumption that species predominently shift their ranges polewards under climate change, highlighting instead a strong tendency to move into deeper waters. This suggests a nuanced response of marine fish populations to environmental variability, with species having their movements constrained by geographical barriers (e.g., southern coasts of England). This result is further supported by the observed southward shifts in the northern MedS, which were likely attributable to the region’s bathymetry, where depth generally increases with southward movement. This widespread deepening trend, observed across both the NeA and MedS, is not surprising, as the three-dimensional nature of bathymetry creates a highly diverse mosaic of environmental conditions within ecosystems^1^. This diversity, in turn, provides potential refuges for populations experiencing changing conditions^27,28^, underscoring the importance of prioritizing three-dimensional species distribution models when projecting the distribution of marine fishes under climate change scenarios^29^. Consequently, including the bathymetric dimension in marine conservation prediction and planning models appears essential for more accurately capturing the spatial dynamics of species^30^. Furthermore, because bathymetric heterogeneity may offer refuges for fish populations^31^, protecting areas with diverse environments and depths should enhance the resilience of marine communities to climate change.

Our findings highlight that, contrary to common assumptions, sea temperature was not the primary driver of distribution shifts. Instead, these shifts were largely determined by species traits and environmental safety margins in response to a broad range of environmental changes, while fishing pressure emerged as a minor contributor. However, the fact that fishing effort is generally higher in shallow waters, particularly from artisanal and recreational fisheries, may still have contributed, at least to some extent, to shifts in population centroids through the uneven removal of individuals. Interestingly, in 27 out of 31 survey time-series, fishing effort declined more rapidly in shallow waters (Supplementary Fig. 24). However, this faster decrease was not sufficient to overturn the bathymetric gradient of absolute fishing effort (Supplementary Fig. 23). Hence, despite a steeper reduction in shallow areas, absolute fishing effort remained higher there than in deeper waters, and thus likely contributed more to deepening than shallowing shifts. In parallel, our niche-tracking approach demonstrated that most species experienced less severe environmental changes by relocating, suggesting a behavioral response through movement towards areas with environmental conditions more suited to their ecological niches^28^. Most surprisingly, we observed that, regardless of the direction of the shift (i.e., deeper, shallower, northward, or southward), most species increased in abundance and expanded their spatial range. These relocations could reflect an overall positive trend in population dynamics, affecting both commercial and non-commercial species, and may be partly linked to the gradual reduction in fishing pressure under the Common Fisheries Policy^12,32^, one of the main frameworks for sustainably managing European fisheries. This was supported by the significant temporal trends in abundance among populations, most of those corresponding to exploited species exhibited increasing trends (highly commercial: 61%; commercial: 76%; minor commercial: 75%), with significant positive median values in abundance rate of change (Supplementary Fig. 13). Interestingly, species classified as “of no interest” significantly increased in abundance (Supplementary Fig. 13), thereby suggesting a positive effect of the reduction in fishing effort. However, all these patterns do not allow us to attribute causality. The observation that a substantial percentage of populations (i.e., 33 to 48%; Supplementary Fig. 16) responded to environmental changes in ways that did not align with their expected ecological niches. This pattern is consistent with, but does not demonstrate, the potential influences of fisheries management. Further work, including targeted analyses and broader spatial replication, is required before drawing firm conclusions about the role of fishing reduction in shaping these trends. Additionally, we could not determine whether these spatial relocations resulted from changes in larval dispersal, the physical movement of adults, or demographic shifts across different locations. To address this uncertainty, we strongly support the recommendations by ref. ^1^ to prioritize the use of dedicated methodologies, such as electronic tags to track adult movements, or genomic approaches combined with otolith ecogeochemistry to study larval dispersal trajectories. Similarly, to distinguish spatial changes in abundance from actual movement, it would be relevant to compare estimated shifts derived from abundance data with those based on presence-absence data. While negative impacts of fishing on exploited stocks are well-documented^33^, effects of fisheries management on population abundance and subsequent range expansion deserve dedicated future work, as this poorly characterised mechanism is likely to mitigate or interact with climate-driven redistribution of marine life. For example, because historically depleted stocks are unlikely to fill their potential environmental or ecological niche, effective fishery management is highly likely to restore exploited stocks, promoting their ability to fill these niches.

Species range shifts may result from an intricate interplay of mechanisms^1^, increasing the risk of drawing erroneous conclusions. In this context, the three-dimensional topology of the seafloor and land mass pattern may constrain species responses locally. For example, species in the northern MedS could theoretically shift poleward but are unable to move directly northward due to landmass and the narrow strait of Gibraltar to the West, which blocks access to the Atlantic^34^. Analogous to the limitations imposed on latitudinal shifts by land barriers (e.g., southern coasts of England), inherent bathymetric constraints likely limited our ability to fully assess bathymetric shifts. Specifically, species that tend to deepen may have been physically restricted by the maximum depth of the study area (the “seafloor barrier”). While our results show relatively low bathymetric shift velocities (9% of the survey depth range per decade), we must acknowledge that this seafloor barrier likely limits the actual deepening potential of certain species, particularly within surveys exhibiting low depth range (Supplementary Fig. 25). Likewise, incorporating the distribution of land barriers is essential for accurately assessing and interpreting changes in species distributions. Hence, accounting for physical constraints that create environmental mosaics (e.g., directional dependence of conditions^35^), therefore appears to be a key step to properly understand the drivers of spatial movement patterns^3,35^. For example, bed shear stress^36^, light availability^37^, seafloor morphology^38^, proximity to upwelling areas^39^, and sediment type^36^ are among the numerous factors that may influence species distribution shifts. Such spatial relocations are also influenced by complex, larger-scale environmental factors^4,35^. In this context, considering natural climate oscillations at oceanic scales is critical for better predicting how species will respond to future environmental changes driven by natural and human-induced climate change^40^. Future research should also consider the capacity of species to coexist, including “prey-predator” relationships through food-web modeling approaches. Constrained by ecological niche conservatism, species may shift their distributions to track suitable climatic conditions. However, because they may not always encounter the trophic resources required for their growth^41^ in these newly occupied areas, some species may fail to follow their expected environmental niches or may even move in directions opposite to the anticipated poleward or deeper shifts. Therefore, further development of advanced numerical approaches that incorporate local hydrography, natural climate oscillations, and biotic interactions should be a priority for this field of research.

Our findings mostly reveal significant deepening shifts, spatial expansions, and increased abundance over recent decades. However, it remains unclear which species can adapt their physiological tolerance to cope with future environmental changes^42^, particularly in ecosystems with intense human activity. For instance, while long-lived species appear more inclined to move to deeper waters, their life history traits, which result in delayed responses to environmental changes, suggest they may face an accumulated extinction debt^43^. The future will not necessarily be defined by depth shifts, as moving to even greater depths might be limited by resource availability^44^, alongside geographical or physiological constraints^45^. Similarly, although excluding species that are poorly sampled by bottom trawl programs (i.e., pelagic species) does little to change the overall pattern (i.e., most species have shifted mainly to deeper waters rather than northward), we wish to emphasize that differences in catchability among species may have introduced uncertainties in the estimated abundances of certain species, such as pelagic species. Consequently, shifts in depth and latitude for pelagic populations should be interpreted with greater caution. Although species-specific catchability estimates are notoriously difficult to obtain due to their strong context dependence^24^ and the large number of species to assess, developing such estimates is a necessary step to reduce potential biases and improve the assessment of population movements from survey data. We should also note that, although the survey time-series scale offers numerous advantages, the limited spatial coverage of these surveys may not have fully captured latitudinal or bathymetric shifts for some species, particularly for highly mobile species (median = 33% of the global scale range; Supplementary Fig. 10). Harmonizing scientific survey protocols and extending their temporal coverage then appear to be key steps in minimizing biases from protocol heterogeneity, particularly when aiming to detect emerging changes and assess the effectiveness of implemented policies at both local and large spatial scales. Finally, it should also be acknowledged that, since bathymetry is often correlated with distance from the coast, population centroids may also have shifted offshore. If so, it would therefore be relevant to examine, using some methodologies devoted to unravel temporal trends of weighted means^46^, whether these potential distributional shifts from coastal to offshore areas were mainly driven by an increase in offshore abundances or by a decrease near the coast, or both.

While there is still considerable room for improvement, our study contributes to a better understanding of how environmental changes influence species, particularly through the use of the niche tracking approach and safety margin traits. Nevertheless, we stress that, alongside these persistent gaps, understanding the mechanisms driving species’ responses to changing environmental conditions is crucial for the effective implementation of climate change mitigation and adaptation policies to tackle the ongoing biodiversity crisis.

## Methods

### Bottom trawl programs and spatial scales used for analyses

We collated fish species abundance data from 92,961 hauls conducted from 1983 to 2019 in the European continental shelf seas across 21 scientific bottom trawl programs (Supplementary Table 6), cumulatively covering approximately 4,800,000 km² in the NeA and MedS (Fig. 1 and Supplementary Fig. 26). To prevent the inclusion of non-representative catch data, hauls associated with excessively low or high trawling speeds were excluded. For each survey, only hauls with speeds between the 5th and 95th percentiles were retained. Additionally, hauls with a trawling duration of less than 10 min were removed to ensure consistency in catch data. Sampling was conducted during daylight hours at an average speed of 3.4 ± 0.5 knots (min: 2.3; 1st decile: 2.8; median: 3.2; last decile: 4.1; max: 5.4) and an average duration of 34.4 ± 13.2 min (min: 10; 1st decile: 22; median: 30; last decile: 60; max: 90). NeA programs locations are gridded into grid cells of 1° longitude by 0.5° latitude (as defined by the International Council for the Exploration of the Sea; ICES). To cover the entire study area with a consistent spatial resolution, we expanded the Atlantic ICES grid to the MedS (Supplementary Fig. 26). We assigned each haul to a grid cell (Supplementary Fig. 26). All captured individuals were identified at the finest possible taxonomic level and reported as a standardized number of caught individuals per species per hour of trawling. We extracted these data from the ICES database on Trawl Surveys (DATRAS; https://datras.ices.dk) in the NeA and the MEDITS database for the MedS^47^. For each survey, every year, a trawling permit was obtained from local authorities to collect the samples. These permits are standard requests made by most European countries whenever their vessels need to operate in foreign waters, and they are submitted regularly each year to each of the embassies. The scientific surveys conducted here are coordinated by the International Council for the Exploration of the Sea ICES: info@ices.dk, and the Mediterranean International Trawl Survey MEDITS group. For the present work, we only considered taxa identified at the species level and their taxonomy was verified and updated using the World Register of Marine Species (WoRMS Editorial Board 2023), leading to a total of 607 species. For the sake of brevity in the paper, the term “population” will encompass the notions of “survey time-series*species” combination.

To investigate depth and latitudinal shifts in species distribution, we conducted analyses both at the survey time-series scale (i.e., one depth and latitudinal shift velocity value per population, where “population” is a species in a given survey time-series) and at the European scale (i.e., one depth and latitudinal shift velocity value per species for the entire European region after pooling all survey time-series together). Conducting analyses at the survey time-series scale offers several advantages: (1) because survey time-series are season-specific, they do not capture seasonal variability in species abundances and distributions; (2) consistency in gear and survey design across the entire time-series and all sampling stations means that all data can be retained for analyses; (3) estimates of species range shifts are replicated thanks to the multiplicity of survey time-series; and (4) each species in each survey time-series can be considered as an independent data point, as they are often demographically independent across different survey time-series^5^. In contrast, the spatial extent of the survey time-series is smaller than the European scale. Consequently, if a species with a very localized range, particularly a highly mobile one, has shifted beyond the survey area, its latitudinal or bathymetric shift may not be fully captured by the survey-scale analysis. We therefore performed analyses at both scales for comparison. At both scales, we excluded survey time-series for which the total number of hauls over the entire period did not exceed 300, resulting in 31 bottom trawl survey time-series across European waters in various seasons and yielding a total of 556 species out of 607. At the survey time-series scale only, to minimize potential biases from undersampled species, we included only those present in the abundance dataset for at least five years. This selection resulted in a total of 414 species out of 556, representing approximately 99.93% of the total abundance.

Due to the geophysical constraint of the Strait of Gibraltar, the European scale analyses were split into two regions: the NeA and the MedS. For each region, we further split the analyses by quarter. Because not all survey time-series were conducted every year, we aggregated years into periods of five years (i.e., 1980–1985, 1985–1990, etc.) and further excluded species that were not present in at least four periods to compute reliable shift velocity values. Additionally, only for analyses conducted at the European scale, and within each of the two regions, species occurring in fewer than 50 hauls were excluded. Finally, we excluded data in grid cells located in extremely deep, shallow, north, and south areas that were not sampled during most of the periods (see selection rules in Supplementary Table 7). For this European scale analysis, the total number of remaining species was 279 out of 556 species, which represents around 99.84% of total abundances.

### Depth/latitudinal shift velocity and species abundance trends

To quantify the velocity of species range shifts along both the bathymetric and latitudinal gradients within each survey time-series, we calculated each species’ CGD in each year as follows^5,21^:

1 $${{{{\rm{CGD}}}}}_{{{{\rm{i}}}}}=\,\frac{{\sum }_{{{{\rm{j}}}}=1}^{{{{\rm{n}}}}}{{{{\rm{Ab}}}}}_{{{{\rm{i}}}},{{{\rm{j}}}}}.{{{{\rm{par}}}}}_{{{{\rm{i}}}}}}{{\sum }_{{{{\rm{j}}}}=1}^{{{{\rm{n}}}}}{{{{\rm{Ab}}}}}_{{{{\rm{i}}}},{{{\rm{j}}}}}}$$

where, for a given population i, Ab_i,j_ is the species density (number of individuals/hour of trawling; all abundance data were square root transformed) in the haul j, par_j_ is the depth or latitude value of haul j. To prevent biased estimates of CGD due to uneven distribution of sampling effort, we randomly resampled the data 100 times to achieve a consistent number of hauls along the latitudinal and bathymetric axes per species per survey time-series. We then calculated the mean of the CGD per year and computed the depth and latitudinal shift velocities as the slope of the linear relationship between CGD_i_ and time (in m/decade and degree/decade, respectively). The standard *t*-test of the lm R function was then applied to identify populations characterized by significant temporal changes of CGD. Finally, because catchability may vary among species^24^, and since all abundance data were obtained from bottom trawl programs, we compared the prevalence of populations shifting to deeper waters (i.e., the percentage of populations showing an increase in the mean depth of their centroid) with and without pelagic species, which are known to be less effectively sampled by bottom trawls than demersal species. In parallel, the same approach of removing pelagic species was used to test the stability of the prevalence of northward shifts (i.e., the percentage of populations showing an increase in the mean latitude of their centroid).

Because the latitudinal range of each population within a given survey time-series may represent only a fraction of its full global distribution (Supplementary Fig. 10), our ability to detect latitudinal shifts may be limited. To assess this limitation, we conducted a complementary analysis to determine whether the balance between northward and southward shifts remained consistent across populations pools stratified by their relative latitudinal range—that is, the proportion of a species’ global latitudinal distribution encompassed by the population’s range within a given survey time-series. To do so, latitudinal values associated with global scale occurrences data were extracted from the OBIS and GBIF platforms through the occurrence function of the robis R package^48^ and the occ_download function of the rgbif R package^49^, respectively.

Since a depth and latitudinal shift velocity value was computed for each species within each survey time-series, we calculated the median velocity of these shifts across all survey time-series for each species. This allowed us to compare the number of species that predominantly shifted deeper versus shallower and those that shifted northward versus southward. Finally, within each survey time-series, we assessed the temporal trends of species abundance from the relationship between the yearly mean species abundances and time.

As an additional analysis to further evaluate how species’ spatial geographic distribution has changed over time (e.g., expansion vs. contraction), we applied the method described in ref. ^15^, which assesses temporal changes in species’ spatial extent along a latitudinal gradient. First, for each species in each survey time-series, we calculated the percentage of grid cells where the species was present each year (“species’ spatial extent”), relative to the total number of grid cells where at least one haul was conducted that year. The slope of the relationship between spatial extent and time was then used to determine whether the species experienced a “contraction” (negative slope) of an “expansion” (positive slope) of its range. Second, for each population, an index of latitudinal position within the species biogeographical range was estimated using the approach developed by Brunel and Boucher (2006):

2 $${{{{\rm{LatPosition}}}}}_{{{{\rm{i}}}},{{{\rm{p}}}}}=\,\frac{{{{{\rm{lat}}}}}_{{{{\rm{s}}}}}-{{{{\rm{lat}}}}}_{{{{\rm{center}}}},{{{\rm{i}}}}}}{0.5({{{{\rm{lat}}}}}_{{{{\rm{north}}}},{{{\rm{i}}}}}-{{{{\rm{lat}}}}}_{{{{\rm{south}}}},{{{\rm{i}}}}})}$$

where, for a given species i in a given survey time-series s, lat_s_ is the median latitude across all hauls performed in the survey time-series s, lat_center,i_ is the center of the distribution range of species i, defined by the mean latitude between the northern and southern limits, lat_north,i_ and lat_south,i_ respectively. To mitigate the potential influence of outliers, lat_north,i_ and lat_south,i_ were defined for each species as the 90th and 10th percentiles, respectively, of the latitudinal values associated with global scale occurrences data (themselves extracted from the OBIS and GBIF platforms through the occurrence function of the robis R package^48^ and the occ_download function of the rgbif R package^49^, respectively). LatPosition_i,s_ values range from −1 to 1 for survey time-series located at the southern and northern boundaries of the species range, respectively, while survey time-series located in the middle of the range will be close to 0. All populations for which lat_s_ is outside of the species latitudinal range (based on OBIS and GBIF) were removed (13.5% of the data set). For all species, we then examined how spatial occurrences have changed along the latitudinal gradient ranging from −1 to 1 to investigate potential expansions or contractions along this gradient.

### Environmental drivers

To investigate the influence of the environment on species shifts along bathymetric and latitudinal gradients, we selected seven environmental drivers based on published literature: sea bottom and surface temperature (respectively SBT and SST), bottom oxygen concentration (O_2_), surface Chl-a, sea bottom salinity (SBS), water acidity (pH), and MLD^50^. We extracted the physical variables (SBT, SST, SBS, and MLD) from the ARMOR3D reanalysis (European Union Copernicus Marine Service 2020) and the biogeochemical variables (i.e., Chl-a, pH, and O_2_) from a NEMO-PISCES biogeochemical model simulation. NEMO-PISCES simulations were forced by reanalysis data (GLORYS2V4-FREE and ERA-Interim for the oceanic and the atmospheric conditions, respectively) and validated by comparison with observed data (European Union-Copernicus Marine Service 2018). We extracted all environmental drivers as monthly averaged values between 1993 and 2019 on a 0.25° spatial grid, and averaged them per grid cell (0.5° × 1°) to match the survey time-series data’s resolution, then calculated annual means. To test the robustness of our results, we also performed analyses by considering the quarterly means of each environmental driver. We finally extracted depth values from the General Bathymetric Chart of the Oceans database at the trawl station scale (GEBCO 2022, see data availability section).

### Testing the “Niche tracking” hypothesis

To test if geographic shifts were driven by a need to escape from adverse environmental conditions to more suitable environments, we calculated and compared two metrics for each environmental driver, per species and survey time-series. The first metric, labeled “experienced environment”, was calculated for each year of the time-series as the species abundance-weighted mean of the environmental driver among all hauls containing the species. This metric was calculated to capture interannual variability in the environmental driver associated with variability in the species distribution. The second metric, labeled “reference environment”, was calculated annually as the average environmental driver value in the area (i.e., one or more grid cells) where the species was caught during its first two years of capture. More specifically, this metric was calculated as the abundance-weighted mean of the environmental driver, where the weights correspond to the mean species abundance within each grid cell during these first two years. These two metrics were only considered for species caught during at least five years. We then compared the temporal trends (i.e., the slope of the linear relationship between each metric and year) of these two metrics, hypothesizing that, in the case of escape from adverse conditions or movement towards favorable conditions for a given fish species, the “experienced environment” will present lower temporal changes than the “reference environment” (Supplementary Fig. 16). Conversely, if the “experienced environment” presents higher temporal changes than the “reference environment”, this would not suggest niche tracking. Finally, we calculated for each environmental driver the percentage of populations that shifted according to the expectations of the “niche-tracking” hypothesis.

### Species traits

Species ability to successfully colonize new habitats partly depends on their reproduction rate and dispersal capabilities^1,51^. Following dispersal, the establishment of populations requires individuals to find suitable food^51^. In parallel, species exhibit a wide diversity of biological responses to climate change^52^, suggesting that range shift may depend on multiple intrinsic species characteristics and external drivers^53^). Thus, the relationship between range shifts and species traits—ecological characteristics that characterize organisms’ responses to environmental change or their effects on ecosystem processes— is a relevant way to better understand species’ responses to climate change^1^. Despite these theoretical expectations, empirical studies rarely succeed at identifying trait-shift relationships. This shortcoming could be attributed to factors like nonlinear associations between range shifts and traits^53^, the influence of biotic interactions, incomplete knowledge across taxa, covariation among traits^53^, and/or methodological shortcomings^54^.

We selected species traits influencing fish responses to environmental conditions and their impact on ecosystem functioning (Supplementary Table 8). These included length and age at maturity, water column position, feeding mode, spawning type, trophic level, offspring size, and the von Bertalanffy K growth coefficient. These trait data were extracted from various sources and collated in ref. ^55^ and from the data-integrated life cycle predictive model^56^, accessible through the FishLife R package^57^.

### Environmental safety margins

Because range shifts are driven by complex interactions between species traits and biotic and abiotic conditions^53^, species traits alone often fail to decipher the processes underlying population responses to climate change^53^. Using metrics that combine climate exposure with species traits, like thermal safety margins, i.e., the difference between the experienced temperature and the upper thermal tolerance limit^1^, may, therefore, provide more insightful analyses than examining traits alone^26,53,58^. There is increasing evidence, particularly in marine ecosystems^2,19^, that latitudinal shifts in response to climate warming^2^ are primarily due to relatively narrow thermal safety margins characteristic of marine species^50^. When considering the multiple dimensions of climate change (beyond rising temperatures), environmental safety margins generally offer a more robust framework than species traits alone for explaining species responses. Surprisingly, safety margins are seldom considered when analyzing past changes in species spatial distribution, although thermal biases are effectively used to predict potential community responses to future warming^58^, and environmental drivers other than temperature are rarely considered. As marine species are known to be sensitive to decreases in oxygen levels^59^, oxygen-related safety margins can, for example, be key to understanding marine biogeographic changes better.

To capture the distance between environmental conditions and species’ lower and upper environmental tolerance limits, we estimated the global range of environmental conditions experienced by the species for each environmental driver (chlorophyll-a, depth, oxygen, pH, salinity, and temperature). We then compared these to the reference environment by extracting occurrence data at the global scale from the OBIS and GBIF platforms through the occurrence function of the robis R package^48^ and the occ_download function of the rgbif R package^49^, respectively. We removed duplicated records shared between the two platforms. We finally overlapped species occurrences with Bio-ORACLE environmental layers available globally from https://www.bio-oracle.org using the load_layers function of the sdmpredictors R package^60^ (Supplementary Table 9). For each species i, we then calculated, for each environmental driver p, the minimum NCmin_i,p_, median (used as a preference metric), maximum NCmax_i,p_, and the difference between the 90th percentile and 10th percentile (used as a tolerance metric). From these values, we calculated how much each species has been exposed to environmental conditions close to its niche limits as follows:

3 $${{{{\rm{NichePosition}}}}}_{{{{\rm{i}}}},{{{\rm{s}}}},{{{\rm{p}}}}}=\,\frac{{{{{\rm{par}}}}}_{{{{\rm{i}}}},{{{\rm{s}}}}}\,-\,{{{{\rm{NCmid}}}}}_{{{{\rm{i}}}},{{{\rm{p}}}}}}{0.5({{{{\rm{NC}}}}\max }_{{{{\rm{i}}}},{{{\rm{p}}}}}-{{{{\rm{NC}}}}\min }_{{{{\rm{i}}}},{{{\rm{p}}}}})}$$

where, for a given species i in a given survey time-series s and a given environmental driver p, par_i,s_ is the mean value of the environmental driver (Chl-a, depth, oxygen, pH, SBS and SBT) over the reference period (i.e., the first two years with species present), NCmid_i,p_ is the center value of the species niche range, defined as the mean between minimum and maximum values of the niche (NCmin_i,p_ and NCmax_i,p_, respectively). The NichePosition_i,s,p_ values range from −1 to 1 when the species experienced mean environmental conditions near the lower and higher limits of its niche, respectively, while NichePosition_i,s,p_ converges to 0 when the species experienced environmental conditions close to the middle of its niche. Finally, we converted the NichePosition_i,s,p_ index into two predictors quantifying proximity to the extreme values of the niche. The first predictor (“Niche position lower tail”), corresponds to the lower values of the niche (i.e., −1 <NichePosition_i,s,p_ < 0) transformed into absolute values, while the second predictor (“Niche position upper tail”), corresponds to the higher values of the niche (i.e., 0 <NichePosition_i,s,p_ < 1) not transformed. Thereby, when these predictors increase towards 1, it indicates that the species experienced environmental conditions near the limits of its niche, providing an estimate of the environmental safety margins relative to both the lower and upper tails of its realized niche. For each environmental driver, we used these two predictors (with species traits and environmental trends) to predict the probability of deeper and northward movements.

Relationship between species traits, environmental safety margins, environment, and fishing on deepening and northward shifts.

To assess the effects of species traits, environmental safety margins, environmental drivers, and fishing on the depth and latitudinal shifts, we implemented an Extreme Gradient Boosting (XGBoost)-based machine learning method. The XGBoost model starts by building an initial decision tree and then constructs a second model that focuses on accurately predicting the observations that were poorly predicted by the first model, defined by their residual errors. Each individual data point is then weighed according to the prediction’s performance. By considering the fit of previous trees, the model continuously strives to improve its accuracy. This sequential approach is unique to boosting and allows the XGBoost model to provide high performance in predictions^61^. Among various machine learning algorithms, XGBoost provides better predictions with faster computation times^62^.

Predictors describing environmental changes (i.e., temporal trends in the “reference environment” for Chl-a, MLD, bottom dissolved oxygen, bottom salinity, SBT, and SST) were transformed into binary variables. Here, 0 indicates a decrease, and 1 indicates an increase in the environmental driver within the “reference environment” over the time-series. The temporal trend in acidity (pH) was not included as a predictor in the model because 99.8% of fish populations were characterized by an increase in acidity (Supplementary Fig. 15). Predictors relative to species traits were kept as continuous variables but log_10_-transformed to mitigate the effect of extreme values. Finally, fishing effect was considered through two predictors: the commercial status of the species (extracted using the species function of the rfishbase R package^63^), and the change in fishing effort in the “reference environment”. Fishing effort data were extracted from ref. ^64^, who provided a global database of fishing activities from 1950 to 2017, mapped to 30-min spatial cells (see Supplementary Fig. 27), and demonstrated comparable patterns to those obtained with Automatic Identification System (AIS) datasets. This dataset offers the advantage of harmonizing multiple sources of data (FAO, governmental data, scientific and gray literature) over a long time-series, while also including small fleets (even those below 15 m compared with AIS) and artisanal segments^64^. As for environmental drivers, the predictor relative to change in fishing effort was transformed into a binary variable (0 indicates a decrease, and 1 indicates an increase in the fishing effort within the “reference environment” over the time-series).

All predictors were normalized using the min-max method to assess their relative effect sizes. Two separate XGBoost analyses were performed: one with “depth shift trend” (0: shallowing; 1: deepening) as the dependent variable and one with “latitudinal shift trend” (0: southward; 1: northward) as the dependent variable. One model including all predictors (i.e., species traits, environmental safety margins, environmental drivers changes, commercial status, and fishing effort changes) was built for each analysis. For these analyses, the commercial status was recoded as 1 for commercial species and as 0 for non-commercial species (Supplementary Table 8). All predictor variables were retained in the model, as XGBoost is robust to multicollinearity and can effectively handle correlated features by selecting the most informative splits during tree construction^62^.

The data set was split into two portions for each of these two analyses: the training set representing 75% of randomly selected populations to train the model, and the testing set to evaluate the quality of the model using the remaining 25% of the data set. The trainControl function (caret R package^65^) was used to optimize tuning parameters in the model (number of trees: 200; learning rate: 0.01). We set the number of cross-validations at five, therefore training it five times on different portions of the training data set before settling on the best tuning parameters. Once done, the 25% remaining rows (“test data set”) were used to predict range shift probability (i.e., deepening for the depth shift trend analysis or northward for the latitudinal shift trend analysis).

To determine the direction of each predictor’s effect, we constructed null models following the approach of ref. ^66^. By doing so, we examined whether the observed relationships between model predictors (i.e., species traits and environmental trends) and the probability of moving to a greater depth or higher latitude were different than expected by chance. For each null model, we randomly permuted predicted probabilities (i.e., probabilities were randomly shuffled between populations in the testing data set). We recalculated the linear regression slope between the model predictor and the predicted probability. The entire XGBoost procedure described above was repeated 5000 times. For each predictor, the mean of differences between the observed and null model slopes were calculated to quantify the strength and direction of each predictor’ effect. We then derived the variable importance (see Fig. 5) by combining the direction (i.e., sign) of each predictor’s effect with its corresponding effect strength. To assess model performances, the accuracy (%) and the adjusted log-likelihood *R*^2^ of McFadden^67^ were computed for each of the 5000 runs and then averaged. Finally, we performed a sensitivity analysis to test the effect of the temporal resolution of environmental predictors (yearly or quarterly mean) by comparing the order of predictor variables’ importance and the sign of their effect on the probability of shifting towards greater depth or northward. This similarity (in %) was calculated from the Spearman Footrule Distance and the Hamming distance for the order of each predictor’s effect size and the sign of these effects, respectively. Only for these analyses aimed at identifying the causes of the shifts, we selected the time window for which data availability was consistent across all variables (i.e., 1993–2019). XGBoost models demonstrated relatively good performance based on accuracy scores and McFadden’s adjusted log-likelihood *R*² values (mean accuracy = 77% and 67%, and pseudo *R*² = 0.24 and 0.23 for the models related to depth and latitudinal shifts, respectively; a McFadden’ *R*^2^ of between 0.2 and 0.4 is considered a “very good fit”)^68^.

Machine-learning models being more prone to overfitting, the area under the curve (AUC) was computed separately for the training data set and the test data set. The relative difference between these two AUC values was used as a diagnostic metric to assess how much performance deteriorated when the model was applied to the test data. Our analyses indicated a moderate degree of overfitting, with the mean relative difference in AUC of approximately 9 ± 10% for the bathymetric model and 6 ± 7% for the latitudinal model. Moreover, two metrics were used to quantify the models’ sensitivity to the removal of predictors. First, we calculated the coefficient of determination of the relationship between predictor importance values from the complete model (i.e., 40 predictors) and those from the reduced models (i.e., from 10 to 38 predictors). Second, we measured the proportion of predictors for which the sign of the estimated effect was preserved across models. Both analyses indicated relatively low sensitivity to predictor omission, particularly with respect to the direction of each predictor’s effect on the probability of shifting deeper or northward, but also regarding predictors’ importance (Supplementary Fig. 28). These analyses showed that predictor importance values were relatively stable regardless of the model used, with a median *R*² of approximately 0.79 ± 0.17 for the bathymetric model and 0.84 ± 0.13 for the latitudinal model.

A last supplementary analysis was conducted in two successive steps to enhance and validate the fishing effect results generated by the XGBoost models. First, for each species within each survey time-series, we estimated the significance of the relationship between species abundance and fishing effort (kW*days at sea) over time with spatial grid cells as a random effect in a Generalized Linear Mixed Model (lme4 R package^69^). In the first step of the analysis, the fishing effect on abundance was estimated by comparing the model with and without fishing effort as an independent variable (ANOVA; all abundance data were square root transformed, only species caught in at least five years were kept, and the temporal window used for fishing effort was selected in order to match with the years were survey data were available). Lastly, the density distribution of depth and latitudinal shift velocities was compared between species that significantly correlated with fishing effort and those that did not.

All statistical analyses were performed under the R environment (R Core Team, 2024).

### Reporting summary

Further information on research design is available in the Nature Portfolio Reporting Summary linked to this article.

## Supplementary information

Supplementary information

Description of Additional Supplementary Information

Supplementary Data 1

Supplementary Data 2

Reporting Summary

Transparent Peer Review file

## Data Availability

Abundance data are publicly available for the North East Atlantic (DATRAS database; https://datras.ices.dk/Data_products/Download/Download_Data_public.aspx). Mediterranean data (MEDITS database) are available upon request to Maritime Affairs and Fisheries (MARE DATACOLLECTIONFRAMEWORK: https://dcf.ec.europa.eu/guidelines_en). Some of the datasets used here are also available in the SEANOE public repository: French NS-IBTSQ1: 10.17882/100023^70^, EVHOE: 10.17882/80041^71^, and French MEDITS: 10.17882/99052^72^). Species traits were collected by extracting traits values from ref. ^55^ openly available on 10.6084/m9.figshare.19833304.v1. It is a combination of the publicly available PANGAEA database (10.1594/PANGAEA.900866), Fishbase information (https://www.fishbase.se/search.php), and inference based on the FISHLIFE project (https://esajournals.onlinelibrary.wiley.com/doi/10.1002/eap.1606). Commercial status of fish species were extracted from Fishbase (https://www.fishbase.se/search.php). Global occurrence data were extracted occurrence data from the OBIS and GBIF platforms (https://www.gbif.org/), thanks to the occurrence function of the robis R package and the occ_download function of the rgbif R package, respectively. Fishing effort data were obtained from Rousseau et al., 2024: https://metadata.imas.utas.edu.au/geonetwork/srv/eng/catalog.search#/metadata/1241a51d-c8c2-4432-aa68-3d2bae142794; doi:10.25959/MNGY-0Q43). Environmental data are all publicly available on Copernicus website (https://data.marine.copernicus.eu/products). Depth values are available from the General Bathymetric Chart of the Oceans database at the trawl station scale (GEBCO 2022: https://www.bodc.ac.uk/data/published_data_library/catalogue/10.5285/e0f0bb80-ab44-2739-e053-6c86abc0289c). All data used to produce all the figures and tables of the present study are available at 10.6084/m9.figshare.28081469.

## code availability

All R codes used to produce all the figures and tables of the present study are available at 10.6084/m9.figshare.28081469.
