# Supplementary material for: Marine fish ranges are expanding and shifting deeper across the European continental shelf
Source: Nat Commun. 2026 Jul 15;17:8702. doi: 10.1038/s41467-026-74371-8 (PMC13490448; doi:10.1038/s41467-026-74371-8)
Supplement: Supplementary file 1 [file 41467_2026_74371_MOESM1_ESM.pdf]

Supplementary information

Title :

Marine fish ranges are expanding and shifting deeper across the European continental shelf

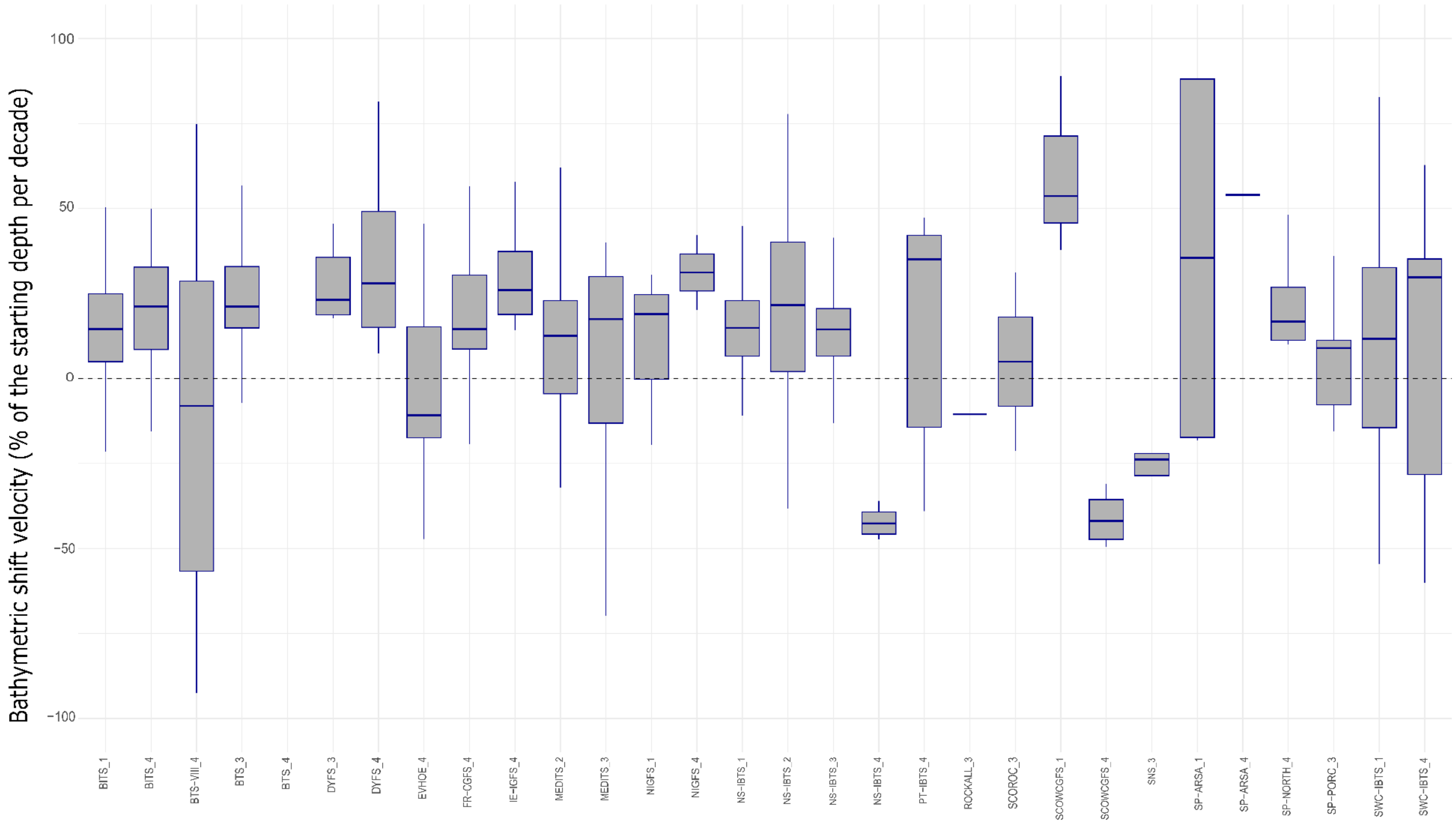

Supplementary Figure 1. **Relative bathymetric shift velocity of fish populations (i.e., the percentage change per decade relative to their starting depth, defined as the depth of the population's centroid in the first survey year in which the population was recorded) across survey time series conducted in the NeA and MedS**.

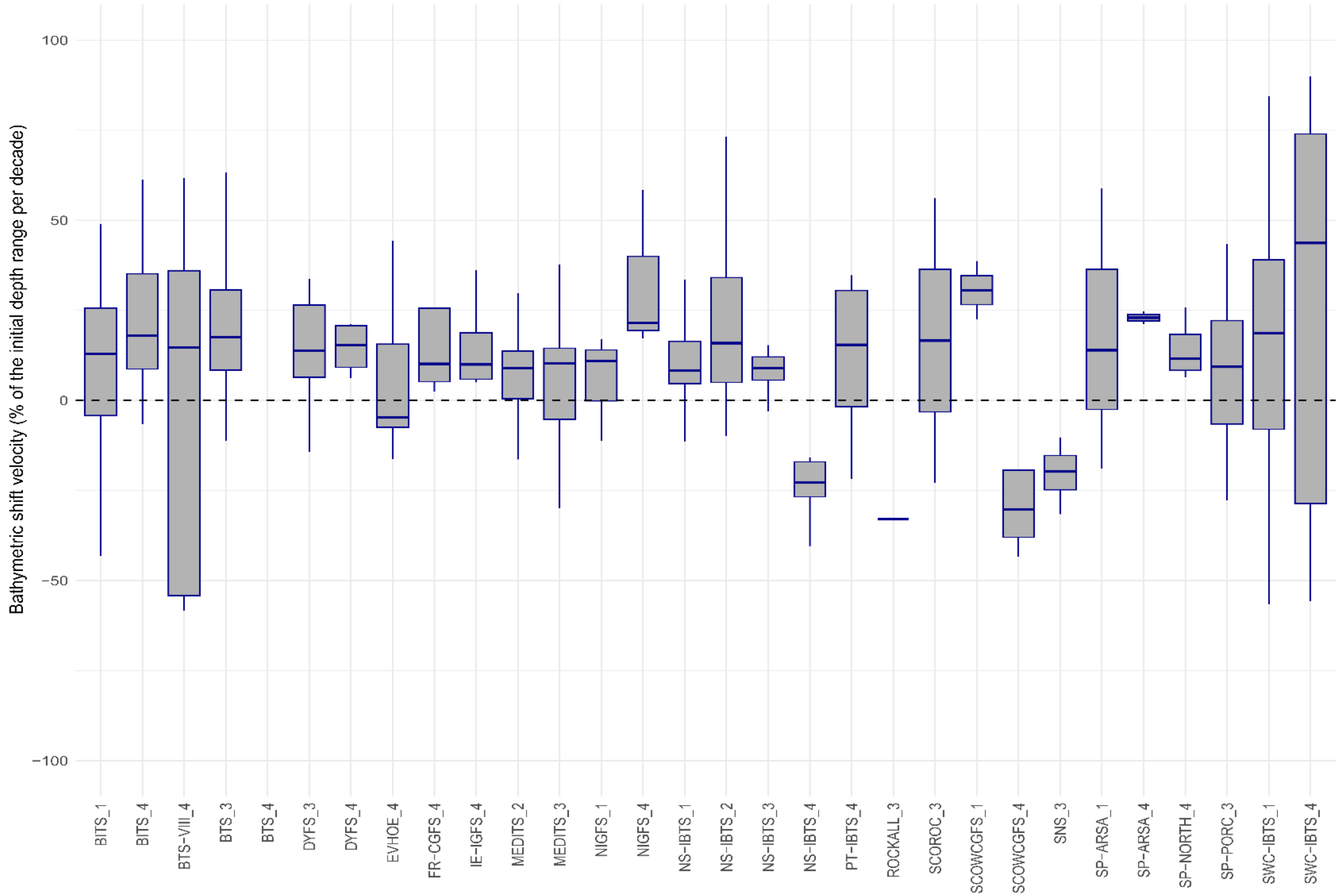

Supplementary Figure 2. **Relative bathymetric shift velocity of fish populations (i.e., the percentage change per decade relative to their starting depth range, defined as the difference between the minimum and maximum depth of the population during the three first years in which the population was recorded) across survey time series conducted in the NeA and MedS.**

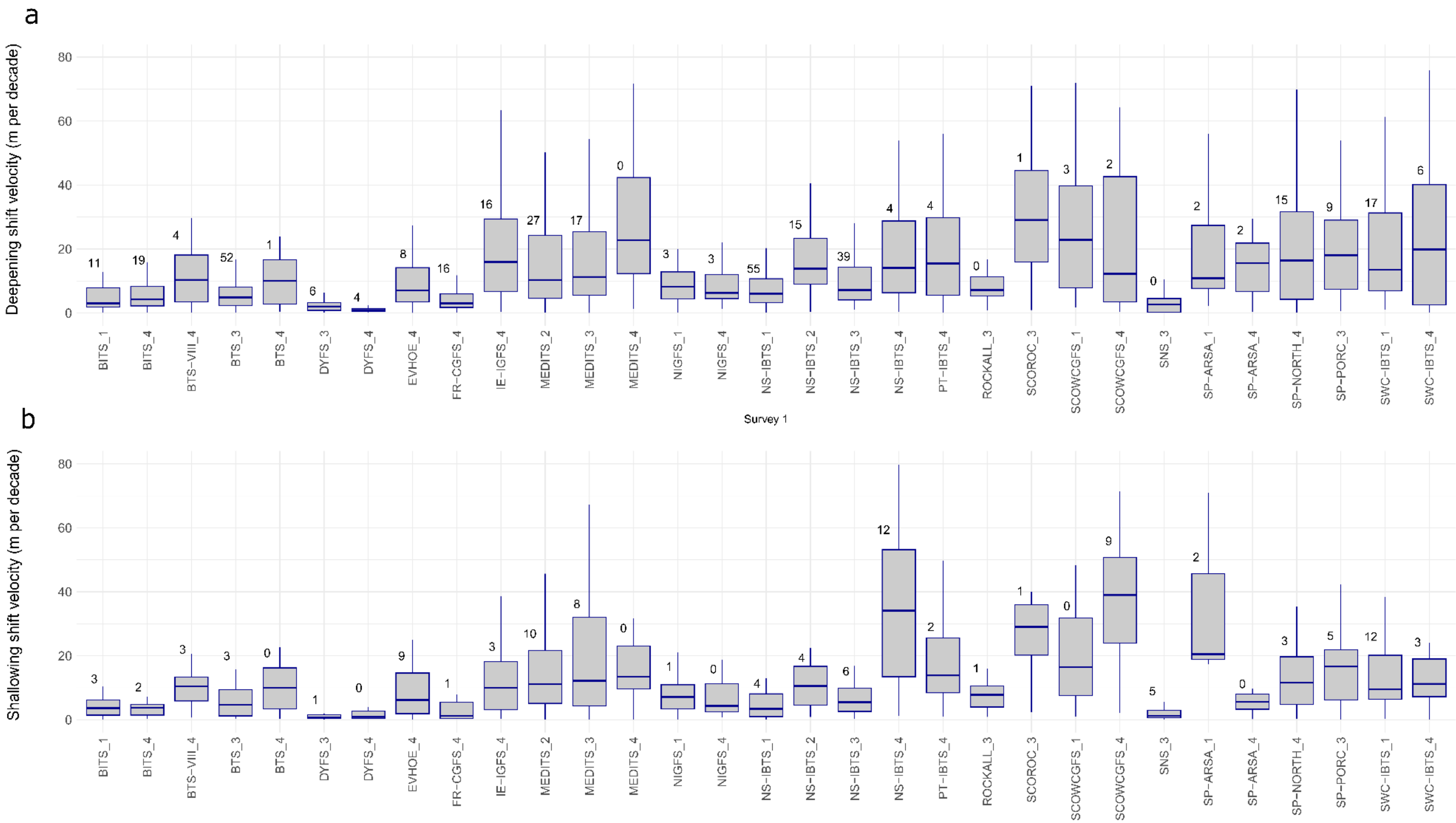

Supplementary Figure 3. **Absolute bathymetric shift velocity of fish populations across survey time-series conducted in the NeA and MedS**. **a**. Only populations exhibiting an increase in the mean depth of their centroid **b.** Only populations exhibiting a decrease in the mean depth of their centroid. The values indicated on top of each box indicates the number of populations exhibiting a significant change in the mean depth of their centroid.

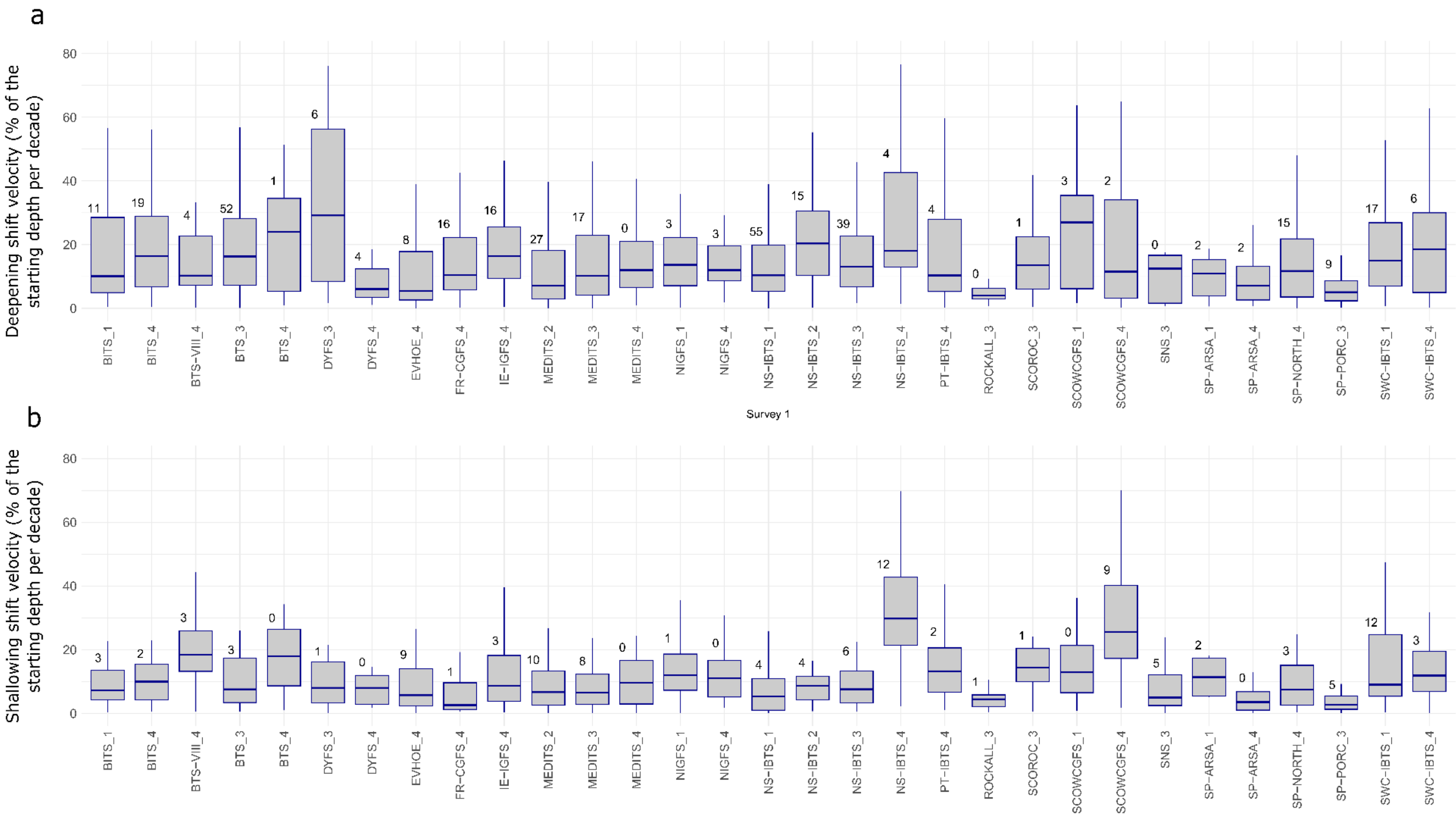

Supplementary Figure 4. **Relative bathymetric shift velocity of fish populations (i.e., the percentage change per decade relative to their starting depth, defined as the depth of the population's centroid in the first survey year in which the population was recorded) across survey time-series conducted in the NeA and MedS. a**. Only populations exhibiting an increase in the mean depth of their centroid **b.** Only populations exhibiting a decrease in the mean depth of their centroid. The values indicated on top of each box indicates the number of populations exhibiting a significant change in the mean depth of their centroid.

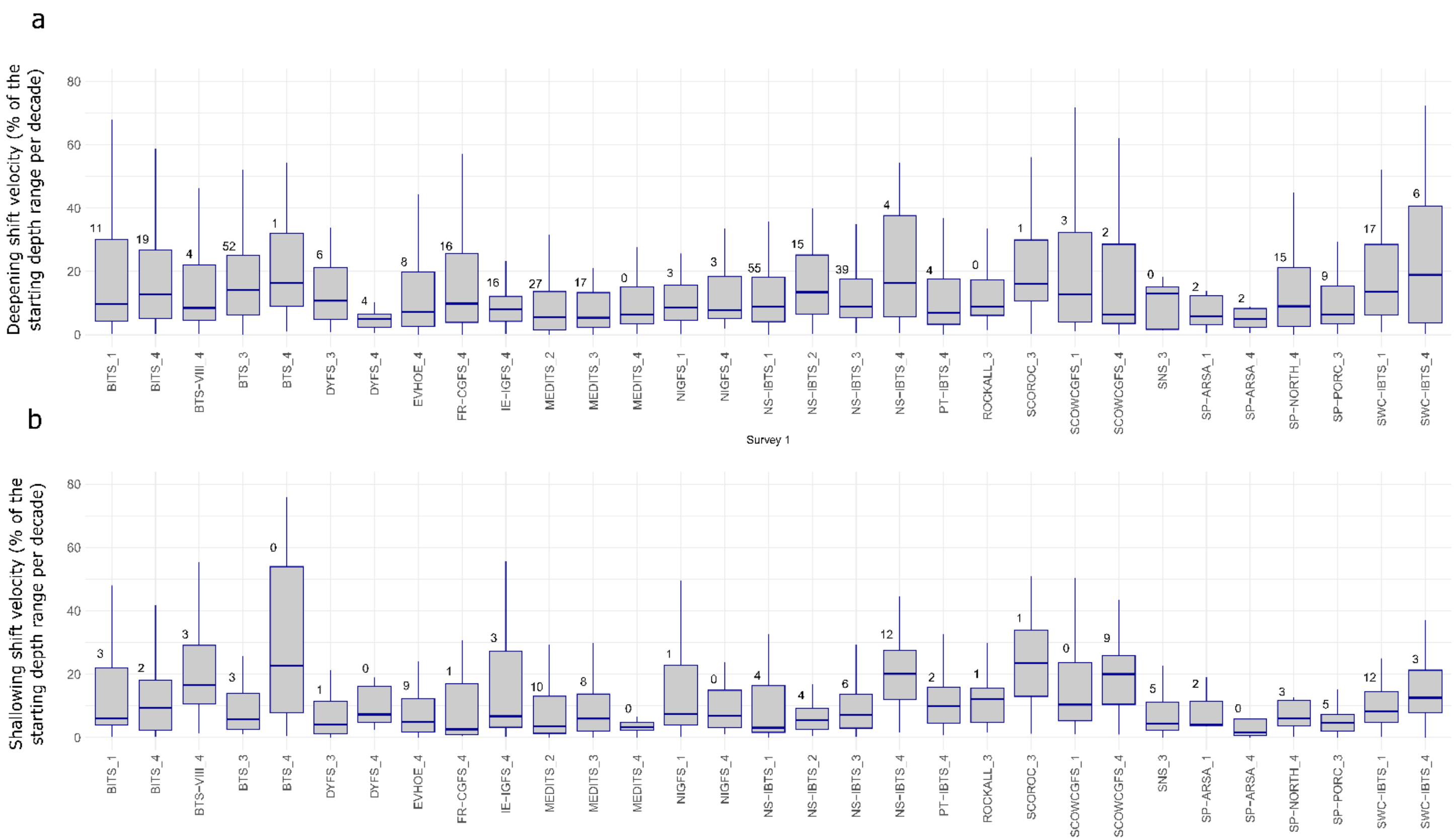

Supplementary Figure 5. **Relative bathymetric shift velocity of fish populations (i.e., the percentage change per decade relative to their starting depth range, defined as the difference between the minimum and maximum depth of the population during the three first years in which the population was recorded) across survey time-series conducted in the NeA and MedS. a**. Only populations exhibiting an increase in the mean depth of their centroid **b.** Only populations exhibiting a decrease in the mean depth of their centroid. The values indicated on top of each box indicates the number of populations exhibiting a significant change in the mean depth of their centroid.

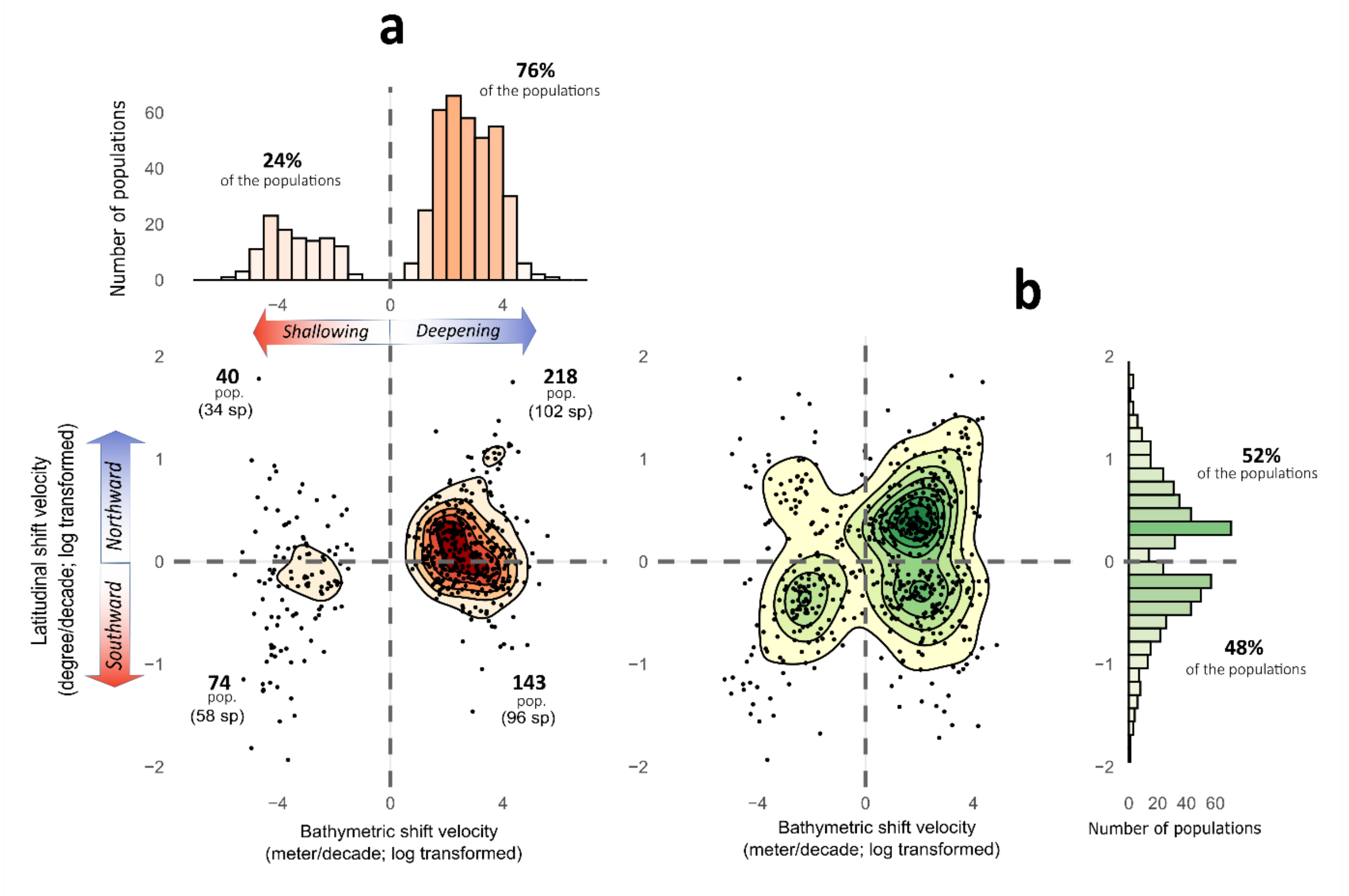

Supplementary Figure 6. **Depth and latitudinal shift velocities of European marine fish populations.** **a**, Shift velocities of the 183 populations exhibiting significant depth shift, **b,** Shift velocities of the 222 populations exhibiting significant latitudinal shift. This analysis only on significant depth shifts was conducted to remove potential noise that might be hovering around low shift values. Orange and green gradients represent the density of dots (i.e. populations) along the bathymetric and latitudinal velocity axes. The density polygons were built using the *stat_density_2d* and *geom_density_2d* from the *ggplot2* R package[35].

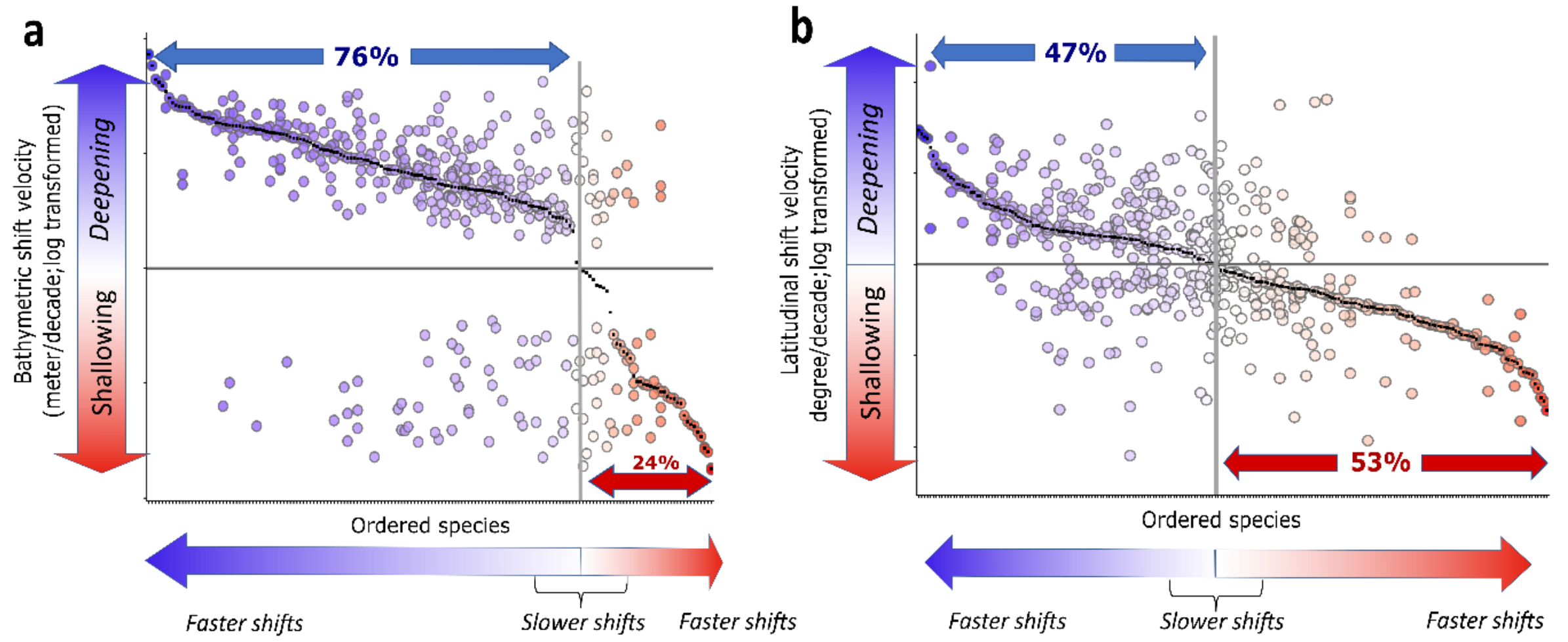

Supplementary Figure 7. **Species ranking based on their median shift velocity across all survey time-series. a**, Species ranking based on their median depth shift velocity. **b**, Species ranking based on their median latitudinal shift velocity. Only species exhibiting significant temporal change in depth and latitudinal position of their centroid of geographic distribution are displayed. The blue and red color intensities correspond to the population' shift velocity, where each dot represents a population (i.e. a species in a survey). A black dashed line represents the median value of a given species (based on all survey time-series where that species was found).

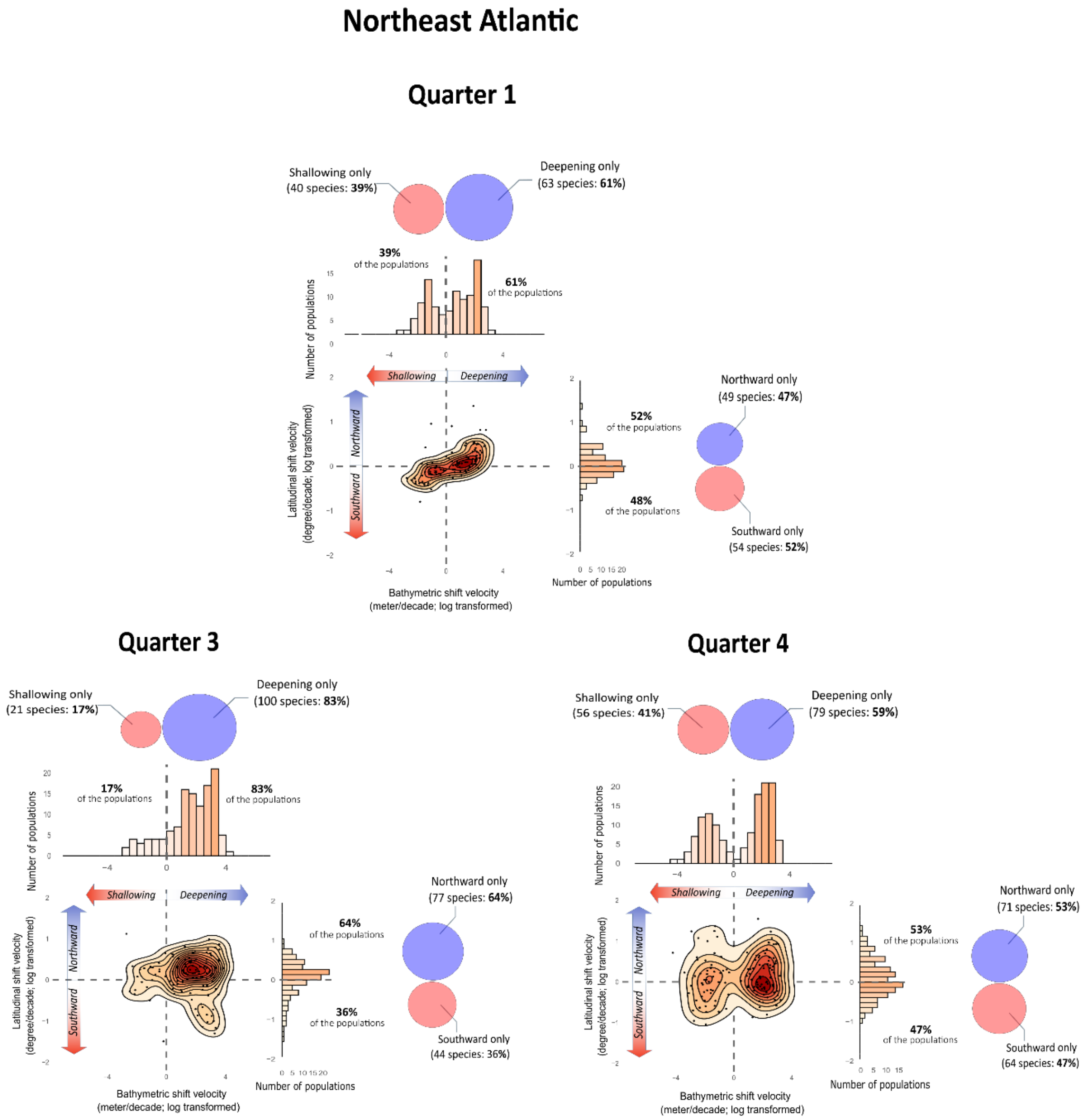

Supplementary Figure 8. **Depth and latitudinal shift velocities of marine fish species in the Northeast Atlantic area per quarter**. Shift velocities of species exhibiting both significant and non-significant temporal change in depth and latitudinal position of their centroid of geographic distribution are displayed and used for calculations. Orange gradients represent the density of points (i.e. populations) along the bathymetric and latitudinal velocity axes. The density polygons were built using the *stat_density_2d* and *geom_density_2d* from the *ggplot2* R package (Wickham *et al.*, 2024).

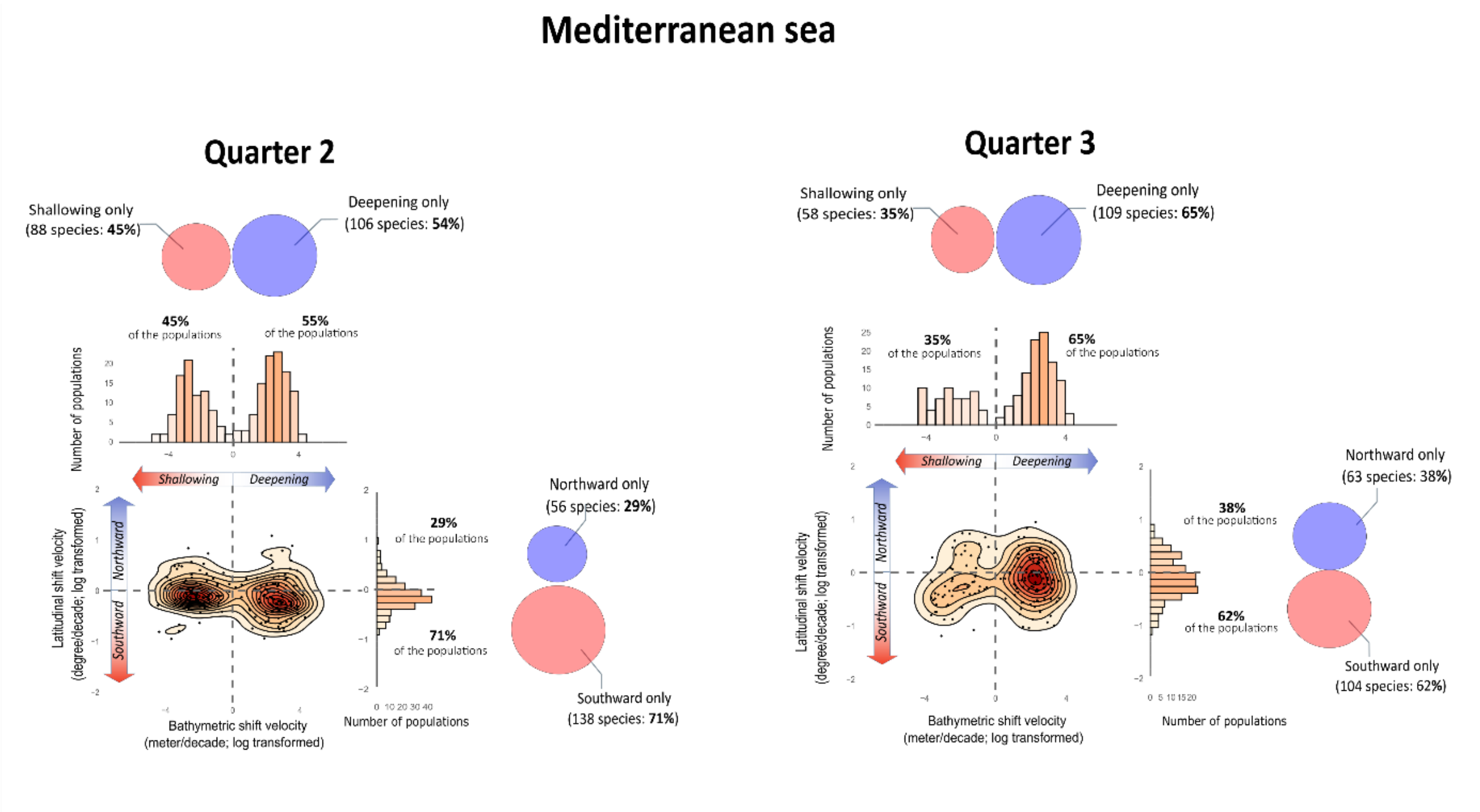

Supplementary Figure 9. **Depth and latitudinal shift velocities of marine fish species in the Mediterranean basin per quarter (2 and 3)**. Shift velocities of species exhibiting both significant and non-significant temporal change in depth and latitudinal position of their centroid of geographic distribution are displayed and used for calculations. Orange gradients represent the density of points (i.e. populations) along the bathymetric and latitudinal velocity axes. The fourth quarter was excluded from the analyses due to insufficient data caused by the short temporal window.

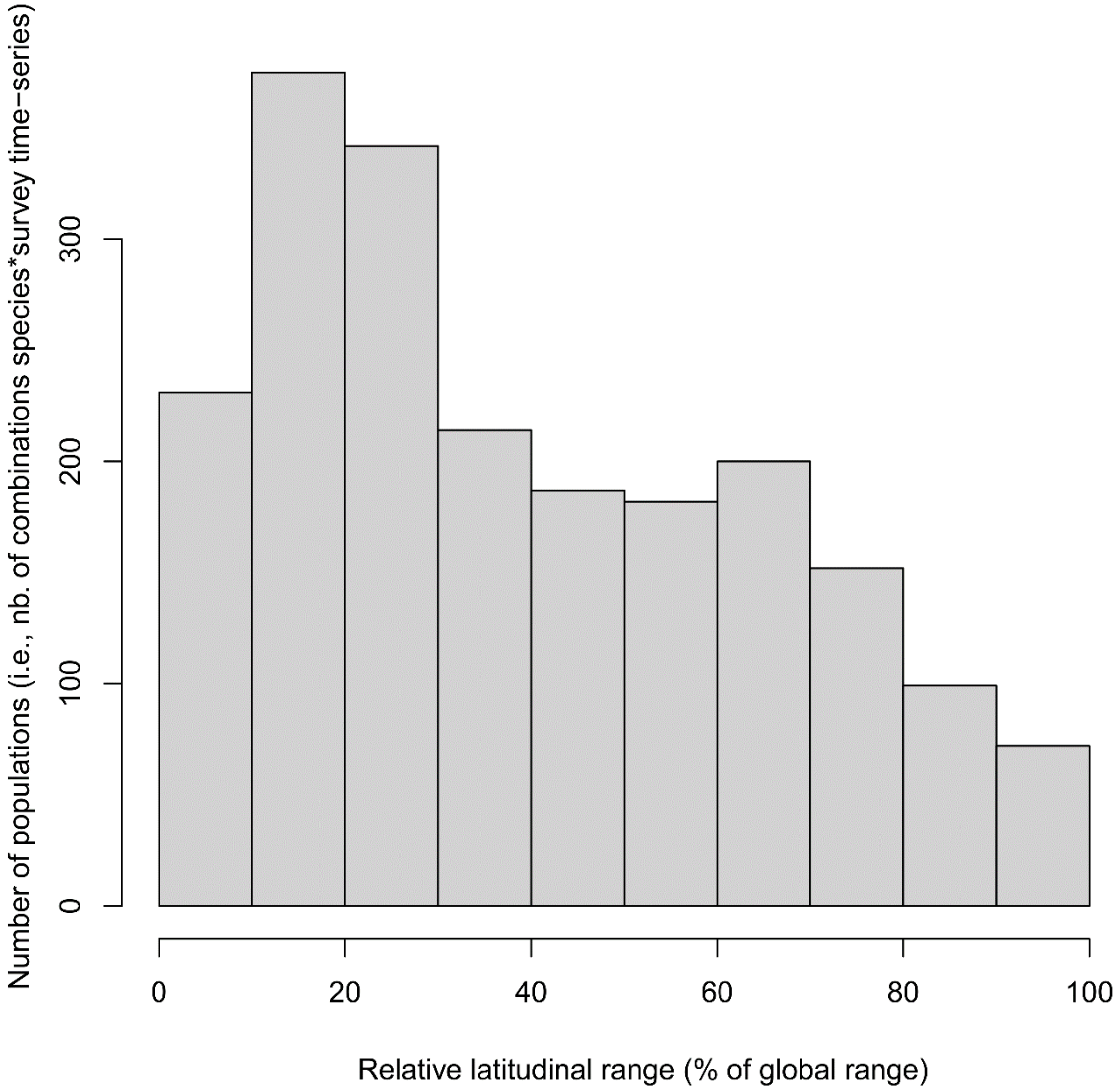

Supplementary Figure 10. **Distribution of relative latitudinal ranges (i.e. latitudinal range of a population within a survey compared with its global-scale latitudinal range) for all fish populations collected by the bottom trawl surveys in the NeA and MedS.**

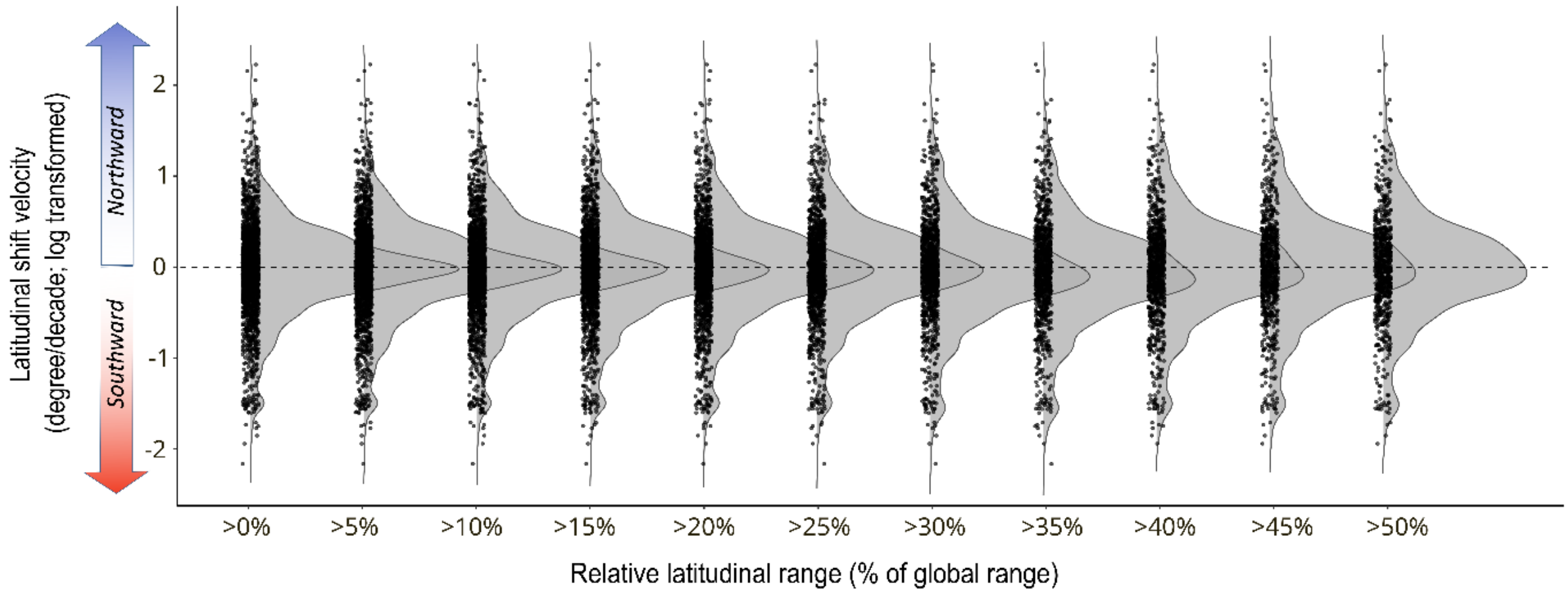

Supplementary Figure 11. **Distribution of latitudinal shift velocities of populations according to their relative latitudinal range (i.e., the latitudinal range of a population within a given survey time-series relative to its global-scale latitudinal range).** Each dot represents a population (i.e., a species within a specific survey time-series). Density polygons were generated using the *density* and *geom_ribbon* R functions from the *ggplot2* package[35].

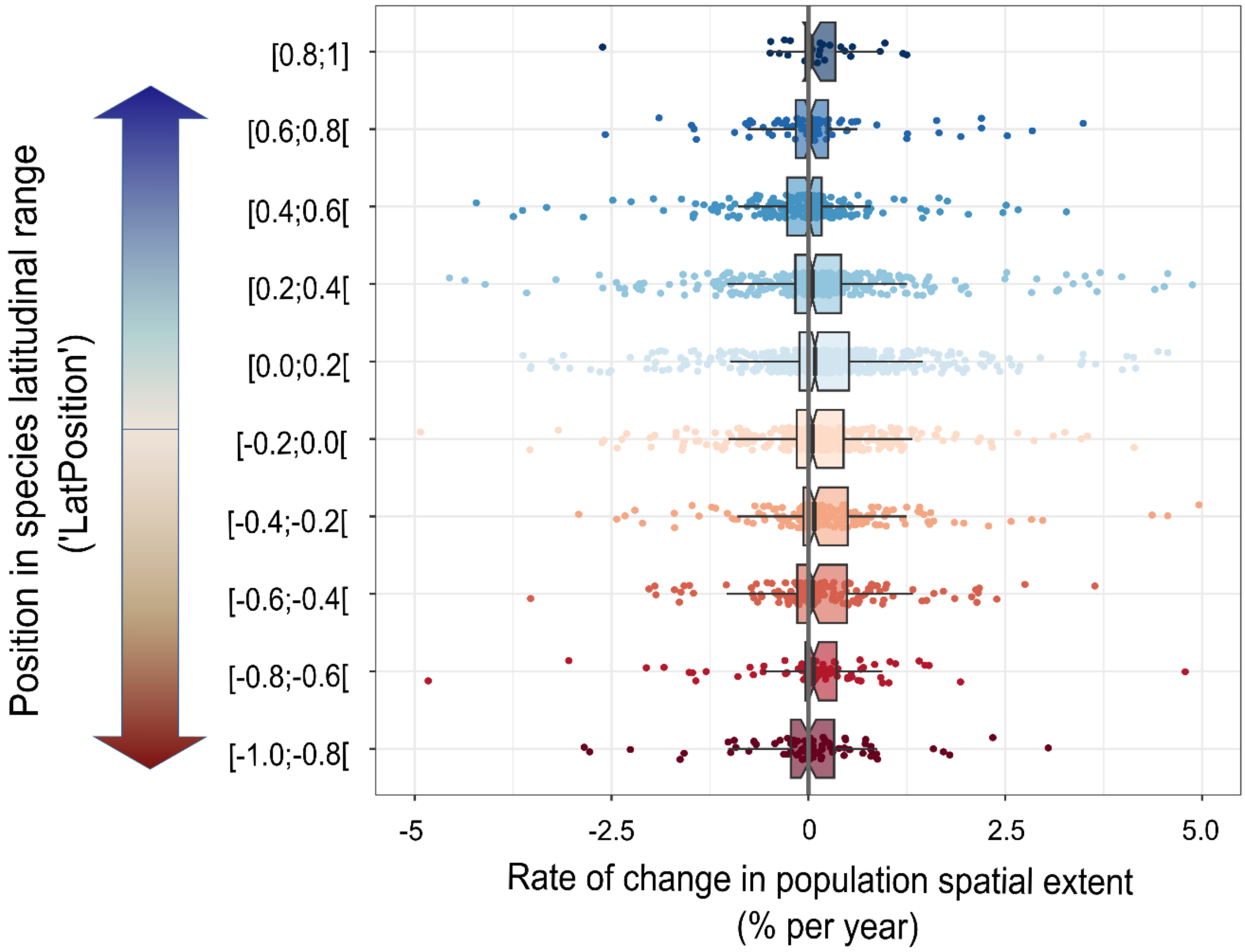

Supplementary Figure 12. **Temporal rate of change in population spatial extent (i.e. the percentage of grid cells occupied by the population relative to all grid cells covered by a given survey time-series) as a function of the survey's position within the species range.**

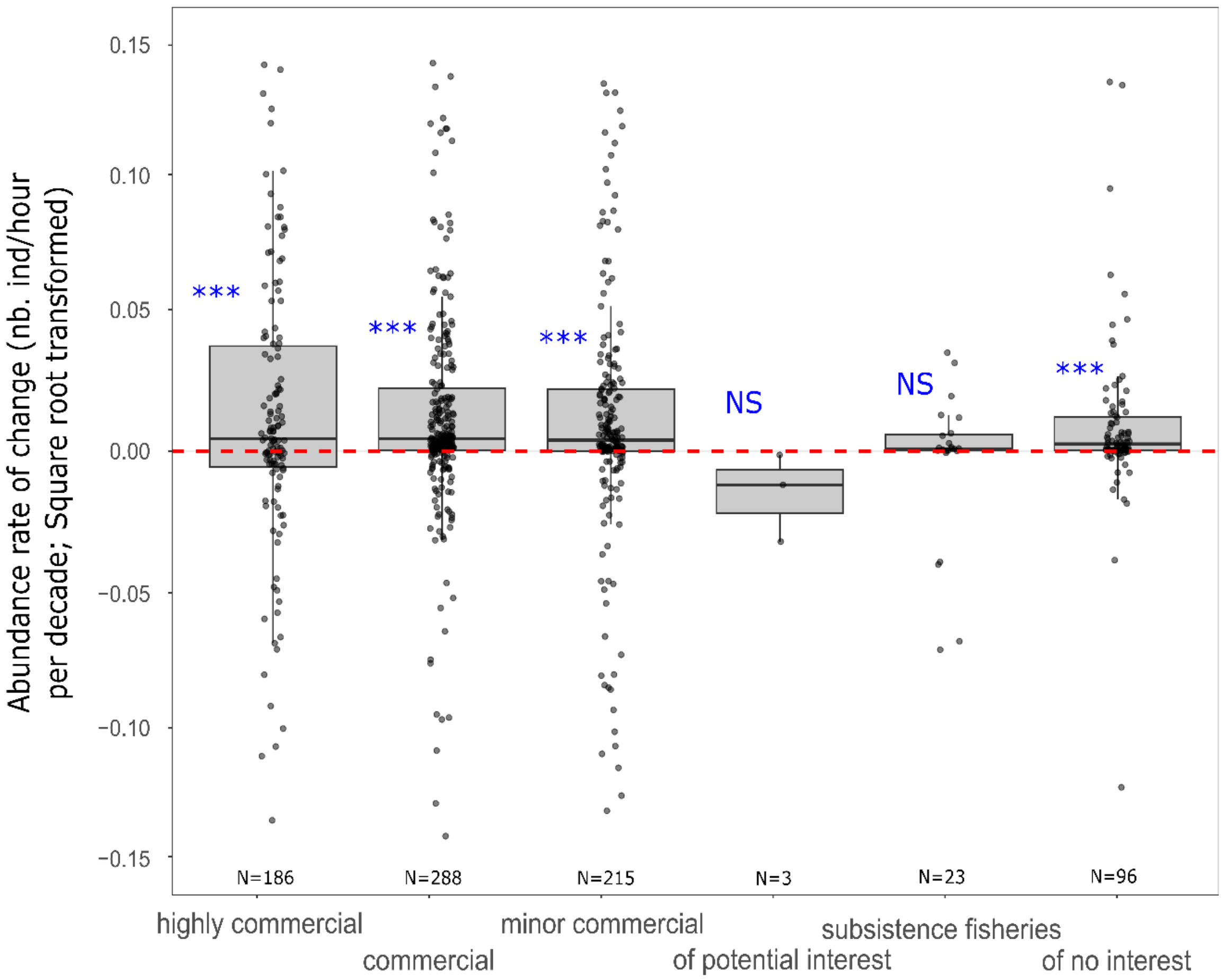

Supplementary Figure 13. **Rate of change in abundance across all commercial-status categories of fish populations during the last four decades in NeA and MedS.** Source data are provided with this paper. Stars represent the level of significance from two-sided Wilcoxon signed-rank tests devoted to compare the mean abundance rate of change with zero (i.e. no temporal trend): *: $0.01 < p < 0.05$; **: $0.001 < p < 0.01$; ***: $p < 0.001$; NS: not significant. Significance values : 'Highly commercial' (p = 4.9e-04), 'Commercial' (p = 1.6e-13), 'Minor commercial' (p = 2.14e-08), 'Of potential interest' (p = 0.25), 'Subsistence fisheries (p = 0.17), 'Of no interest' (p = 6.9e-07).

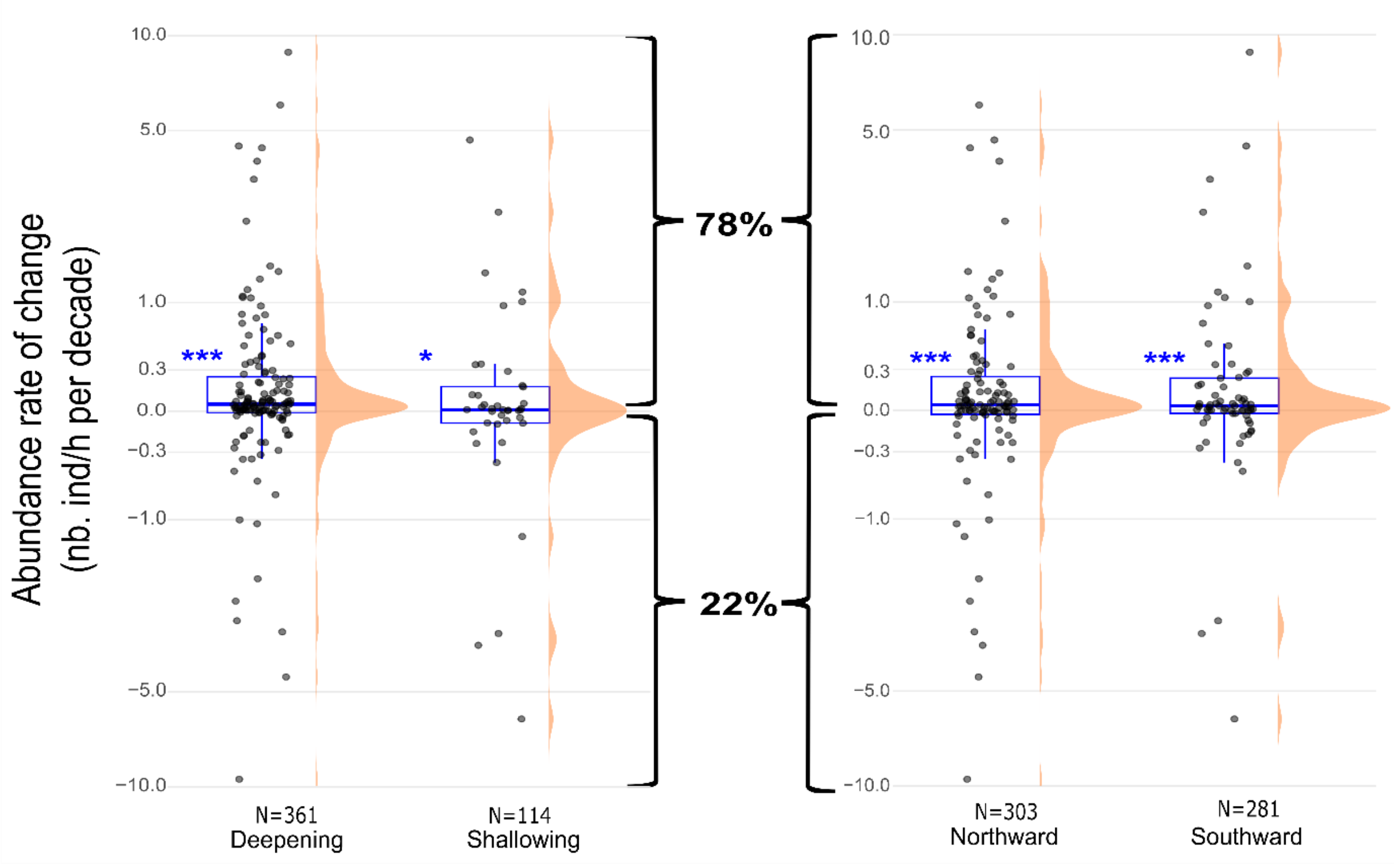

Supplementary Figure 14. **Box plots of the abundance rate of change among populations experiencing a significant deepening, shallowing, northward and southward shift. a.** Populations associated with significant depth shift and **b.** latitudinal shift. Only populations associated with a significant shift are considered. Stars represent the level of significance from two-sided Wilcoxon signed-rank tests devoted to compare the mean abundance rate of change with zero (i.e. no temporal trend): *: $0.01 < p < 0.05$; **: $0.001 < p < 0.01$; ***: $p < 0.001$; NS: not significant. Source data are provided with this paper. Significance values : 'Deepening' ($p = 8.7e-09$), 'Shallowing' ($p = 0.04$), 'Northward' ($p = 2.3e-06$), 'Southward' ($p = 1.9e-09$).

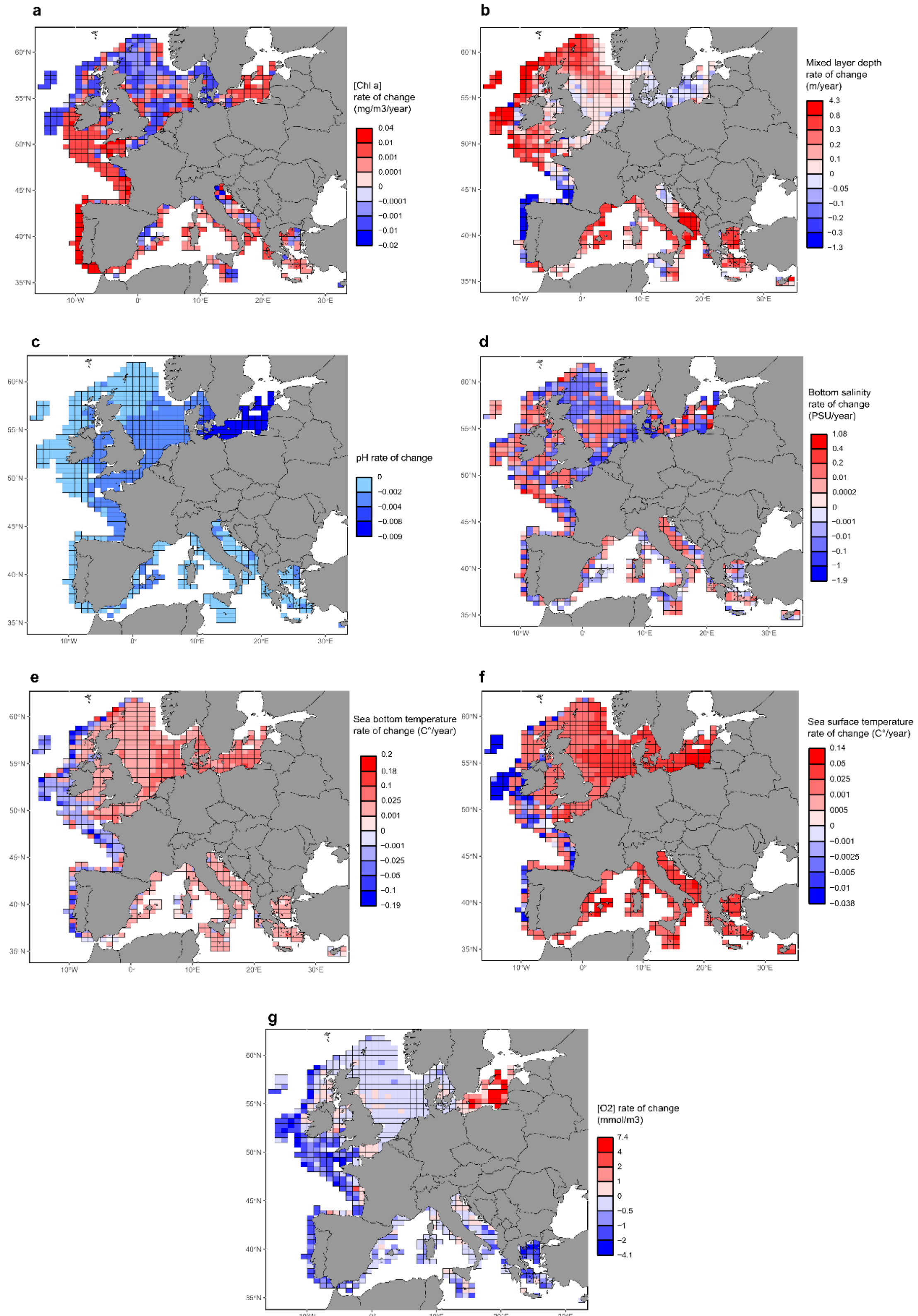

Supplementary Figure 15. **Maps showing the rate of change in environmental parameters from 1993 to 2019 for: a**. chlorophyll-a concentration, **b**. mixed layer depth, **c**. water acidity, **d**. sea bottom salinity, **e**. sea bottom temperature, **f**. sea surface temperature, and **g**. dissolved oxygen concentration. The slope of the linear relationship between each environmental parameter and time (in years) was used as a proxy for the temporal rate of change.

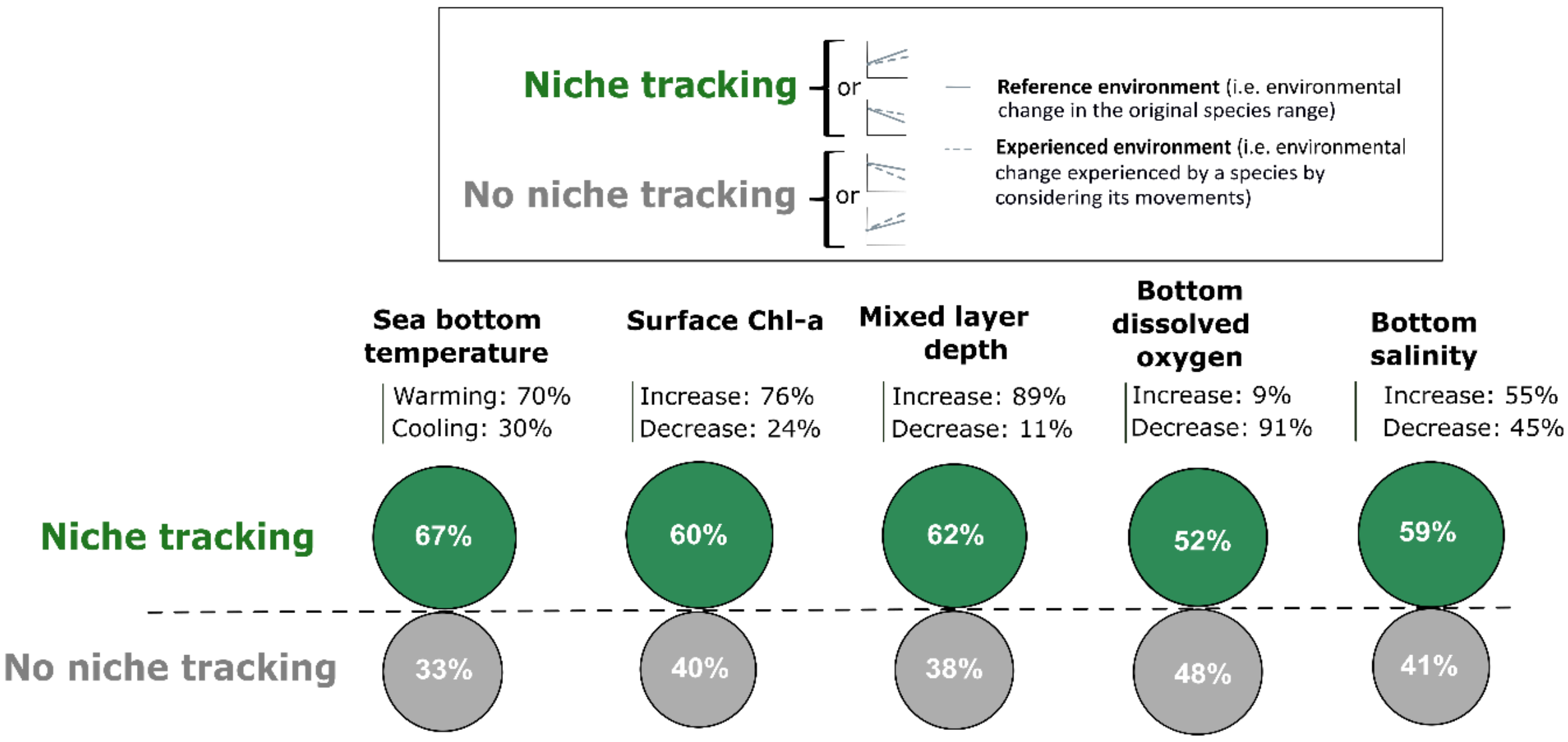

Supplementary Figure 16. **Percentages of populations exhibiting expected and unexpected species responses to environmental change (represented by green and gray sections, respectively).** Only species showing a significant change in their 'reference environment' are selected. Water acidity was not shown as 99% of populations indicated increased water acidity.

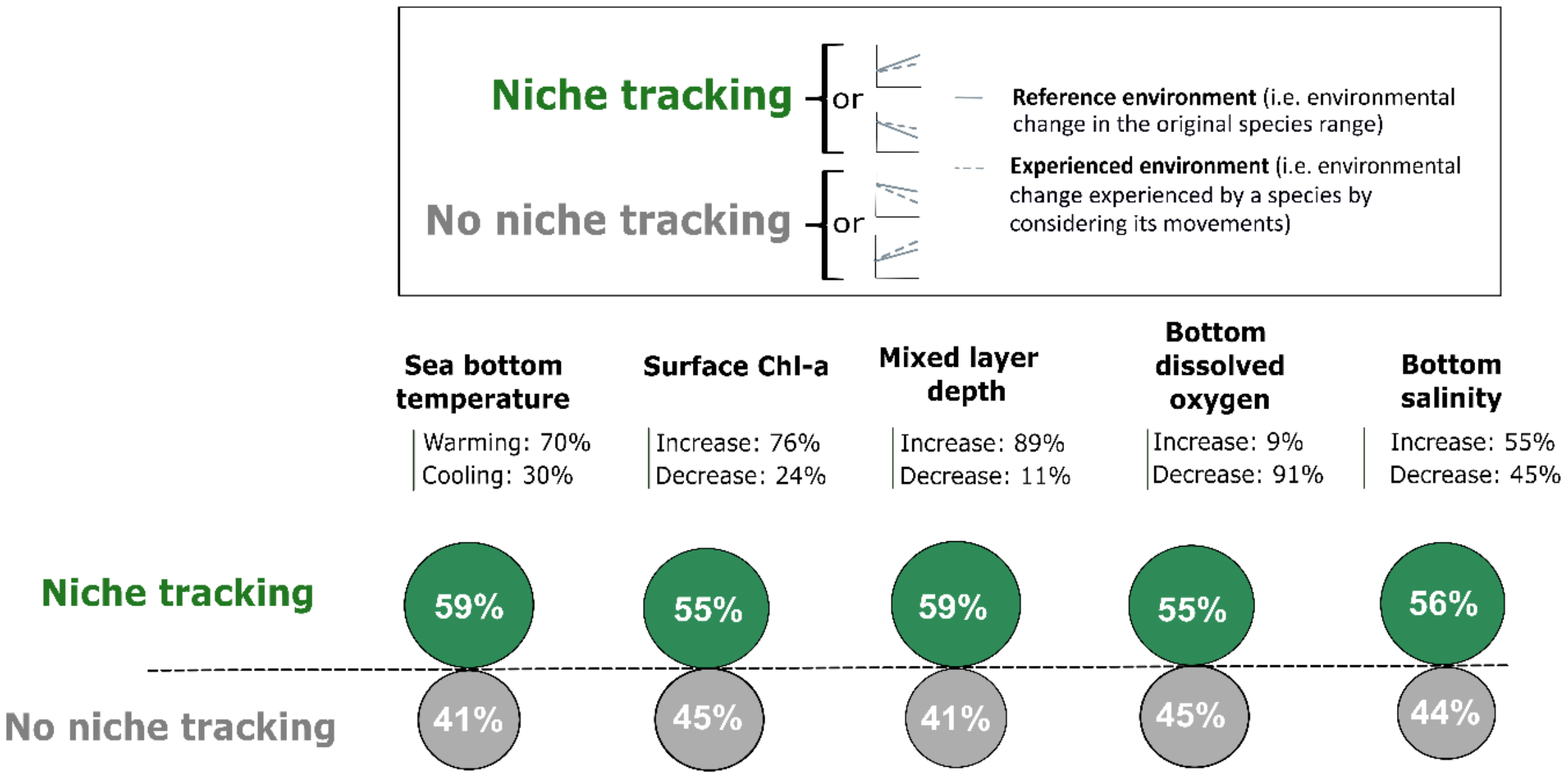

Supplementary Figure 17. **Percentages of population exhibiting expected and unexpected species responses to environmental change (represented by green and gray sections, respectively).** All populations are considered here (i.e. including cases where no significant and significant change in the 'reference environment' was detected).

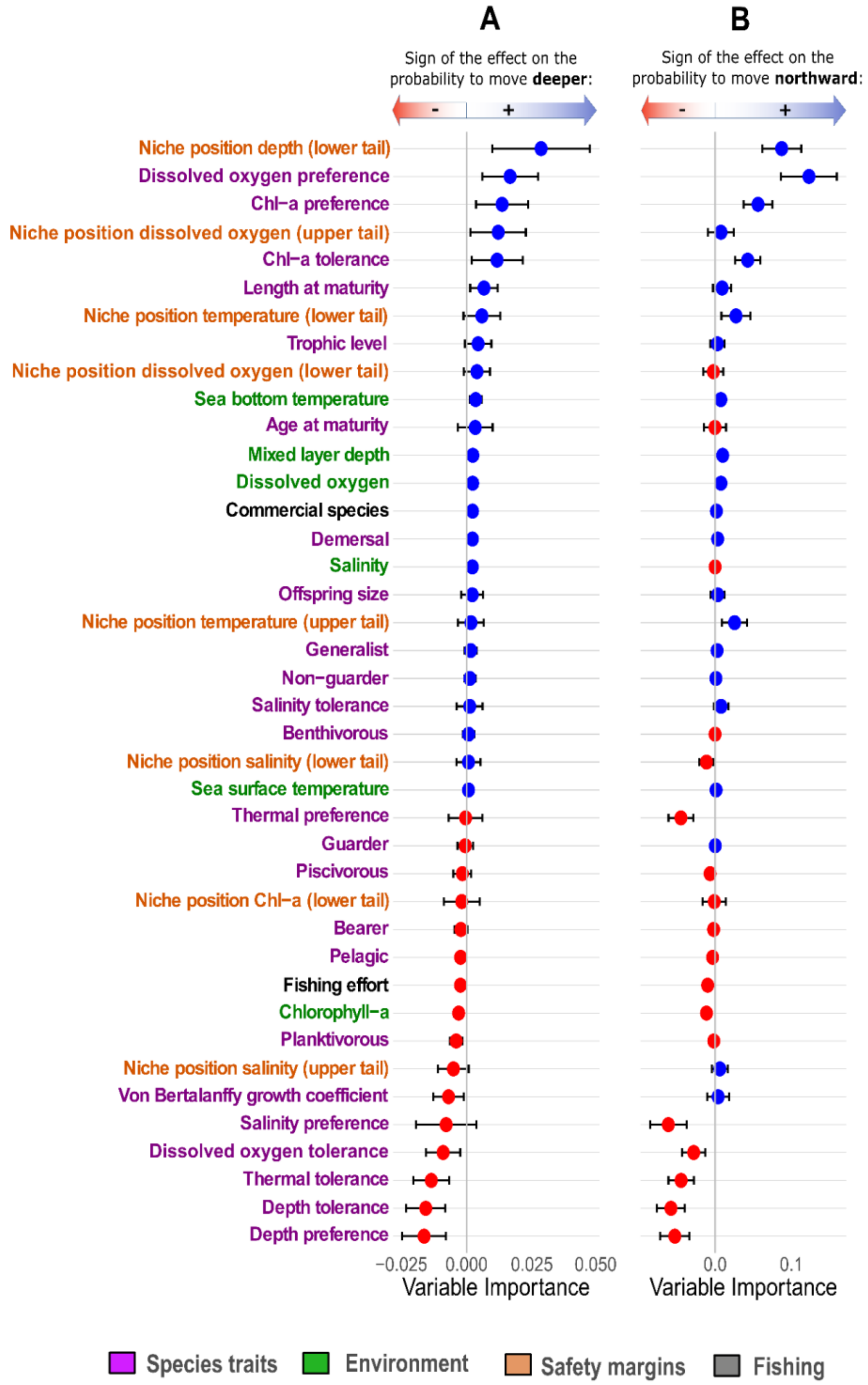

Supplementary Figure 18. **Results from Extreme Gradient Boosting models demonstrating the influence of safety margins and environmental changes on the probability of a species shifting deeper**. **a**. and northward, **b**. Horizontal bars represent standard deviations. Quarterly means of environmental drivers are considered. When predictors relative to 'Niche position' increase, it indicates that the species experienced environmental conditions near the limits of its niche, with 'lower tail' and 'upper tail' corresponding to the lower and upper part of their niche, respectively. Source data are provided with this paper. Horizontal bars represent standard deviations of variable importance derived from 5,000 null model runs (see Methods). For the bathymetric and latitudinal analyses, the total number of samples (i.e., a species within a survey) was 386 and 509, respectively. Each sample is characterized by the temporal trend in bathymetric (or latitudinal) shift, as well as by the set of model predictors (i.e., environmental trends, fishing effort trends, species traits, and safety margins).

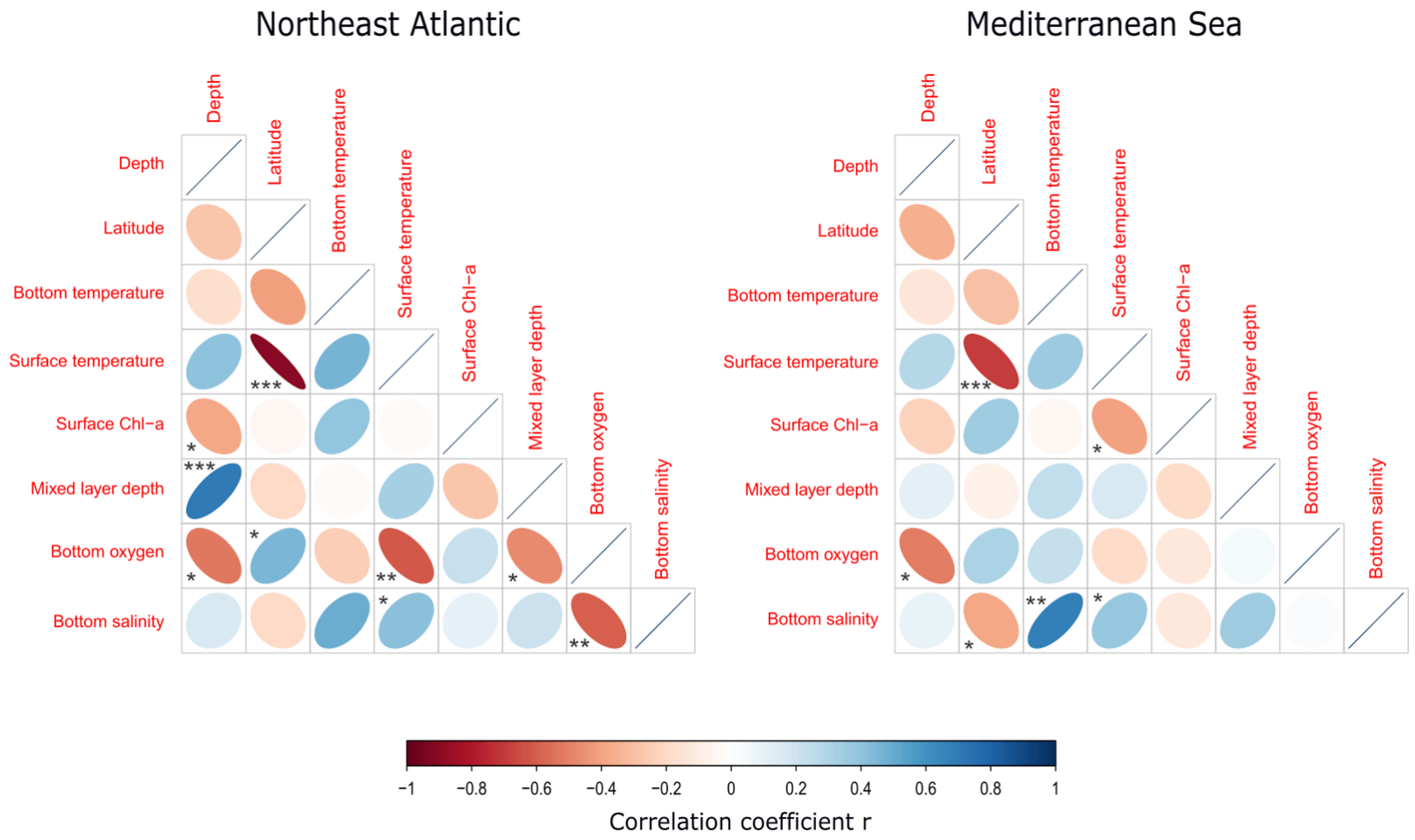

Supplementary Figure 19. **Correlations between environmental drivers in the NeA and MedS.**

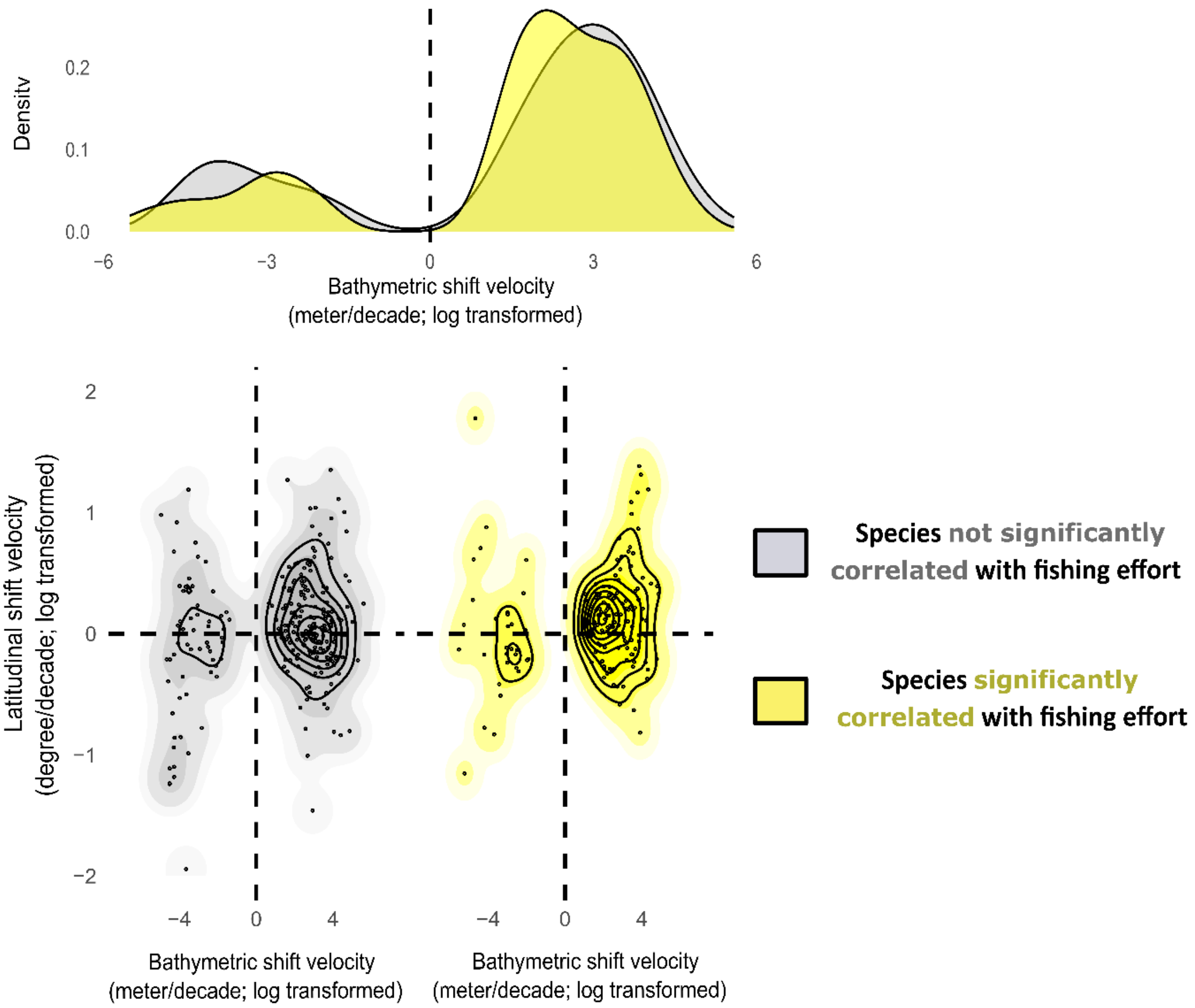

Supplementary Figure 20. **Effects of fishing activities on bathymetric shifts of European marine fish species.** Color gradients represent the density of dots (i.e. populations) along the bathymetric velocity axes. The density polygons were built using the *stat_density_2d* and *geom_density_2d* from the *ggplot2* R package. Only species exhibiting significant depth shift are displayed.

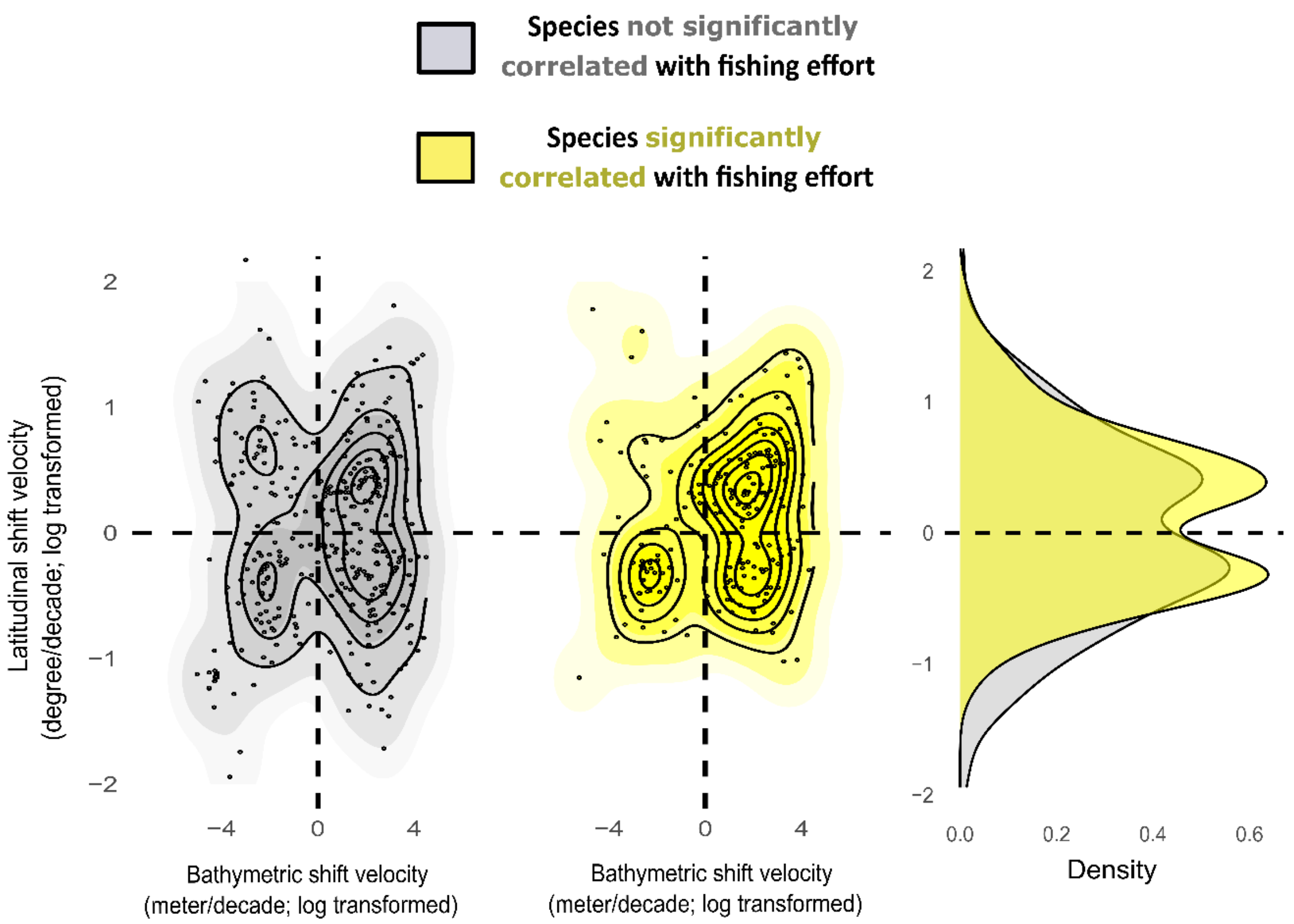

Supplementary Figure 21. **Effects of fishing activities on latitudinal shifts of European marine fish species.** Color gradients represent the density of dots (i.e. populations) along the latitudinal velocity axes. The density polygons were built using the *stat_density_2d* and *geom_density_2d* from the *ggplot2* R package. Only species exhibiting significant latitudinal shift are displayed.

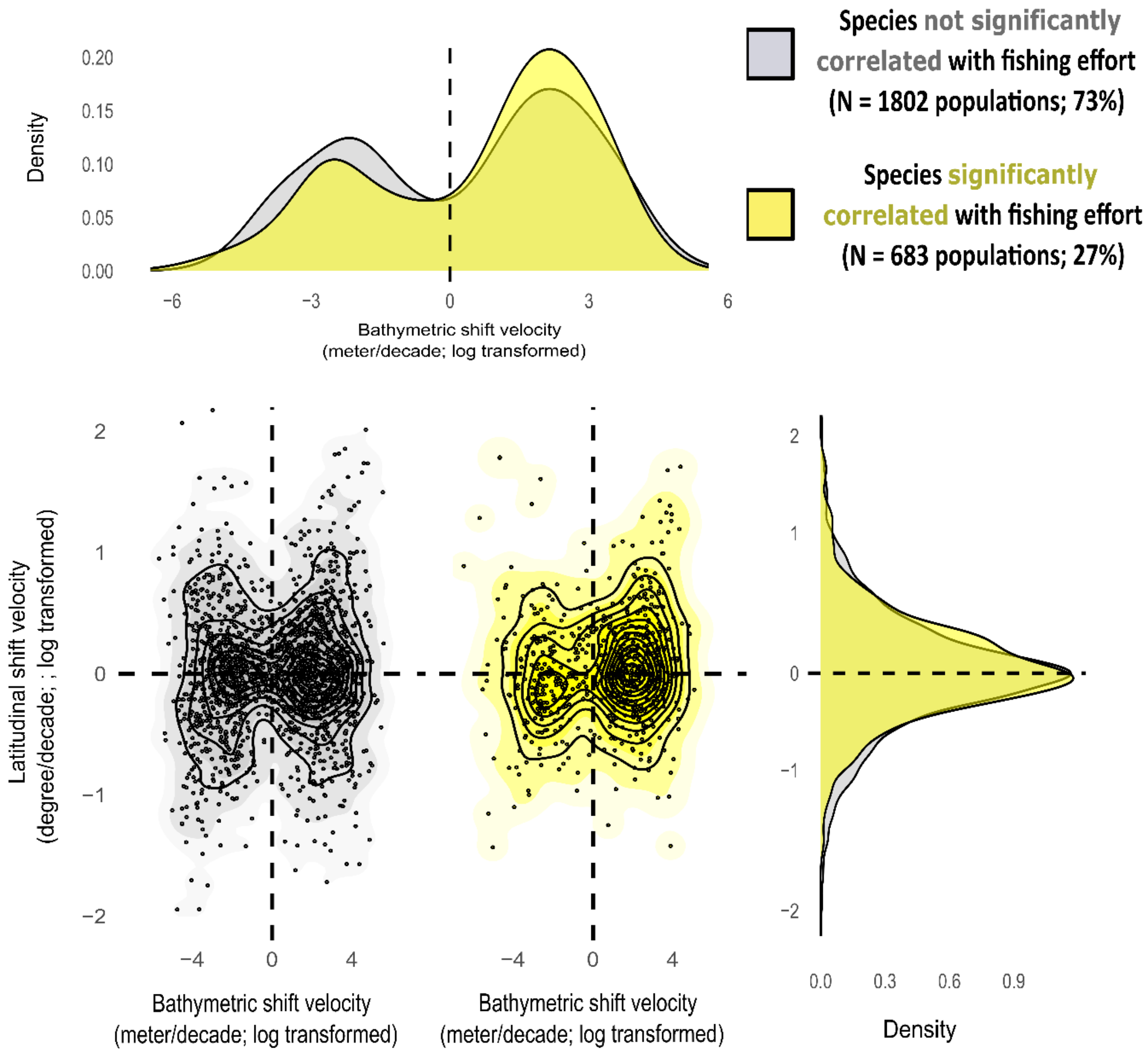

Supplementary Figure 22. **Effects of fishing activities on bathymetric and latitudinal shifts of European marine fish species**. Color gradients represent the density of dots (i.e. populations) along the bathymetric and latitudinal velocity axes. The density polygons were built using the *stat_density_2d* and *geom_density_2d* from the *ggplot2* R package. Both species exhibiting significant and non-significant depth and latitudinal shift are displayed.

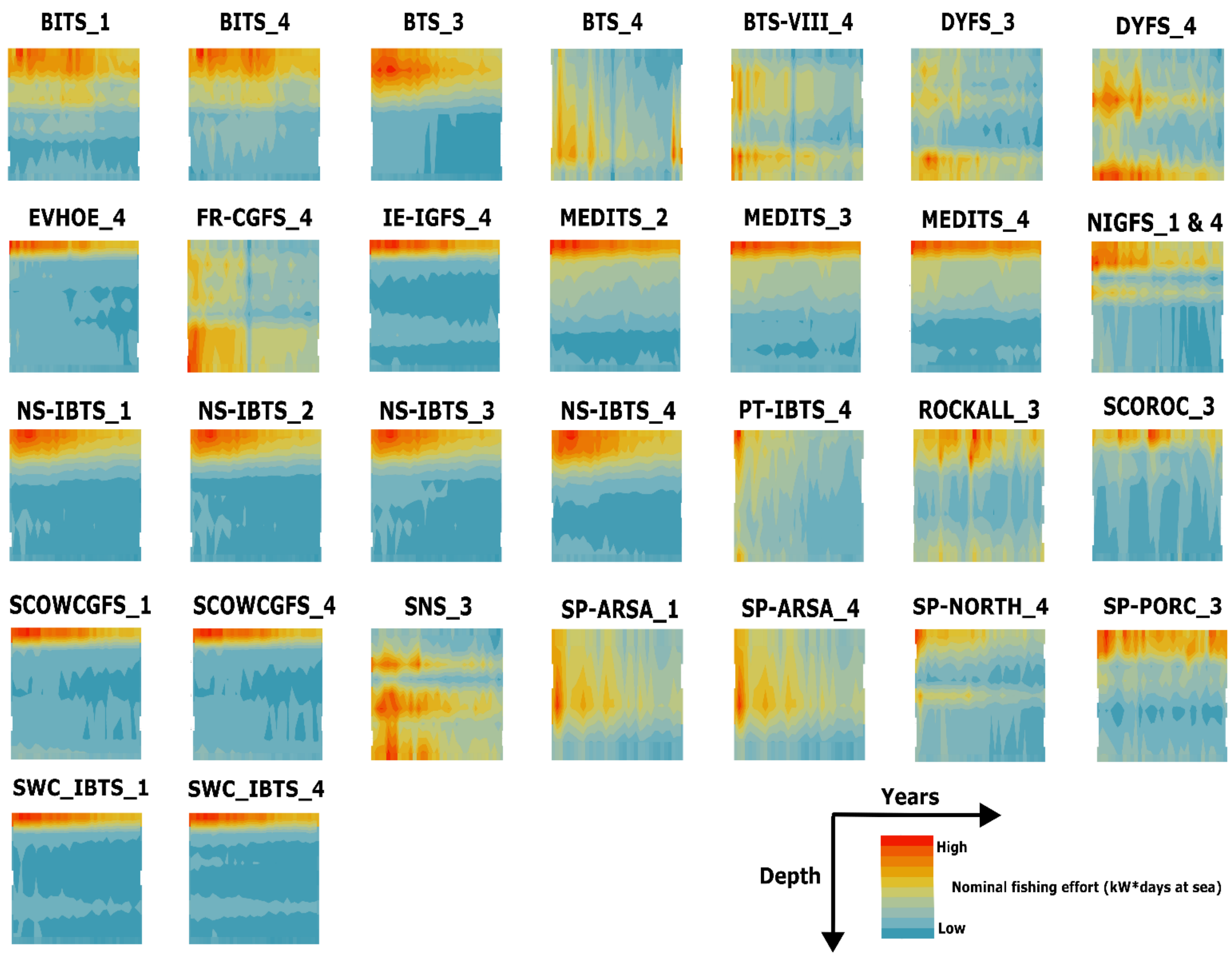

Supplementary Figure 23. **Temporal changes in the distribution of fishing effort along the bathymetric gradient within each bottom trawl survey time-series areas.** The number that follows the underscore corresponds to the quarter.

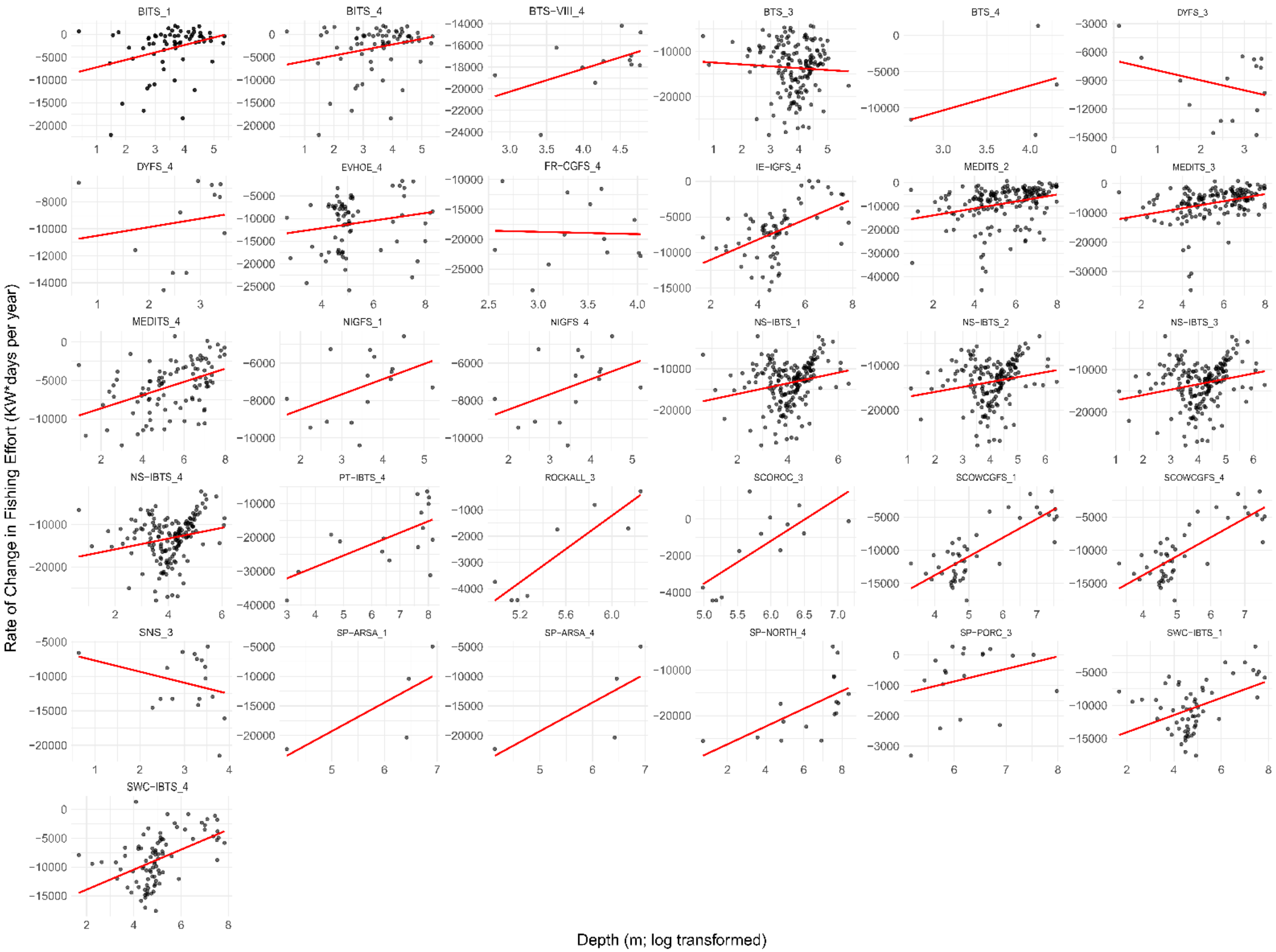

Supplementary Figure 24. **Relationship between the rate of change in fishing effort and depth within each area where bottom trawl programs were performed.**

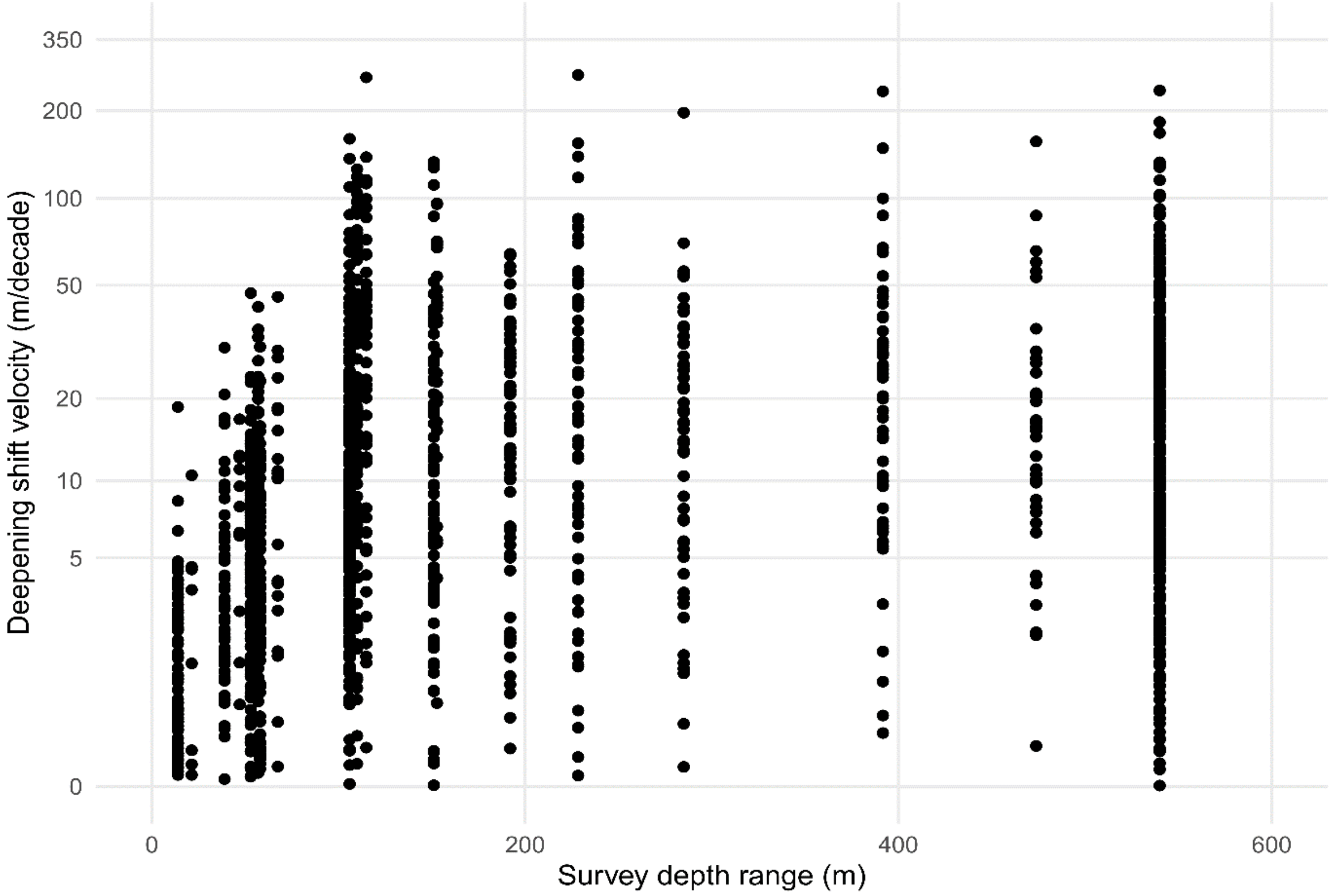

Supplementary Figure 25. **Relationship between bathymetric shift velocity and survey depth range.** Only populations exhibiting an increase in the centroid depth of their geographic distribution are displayed. Each dot represents one population.

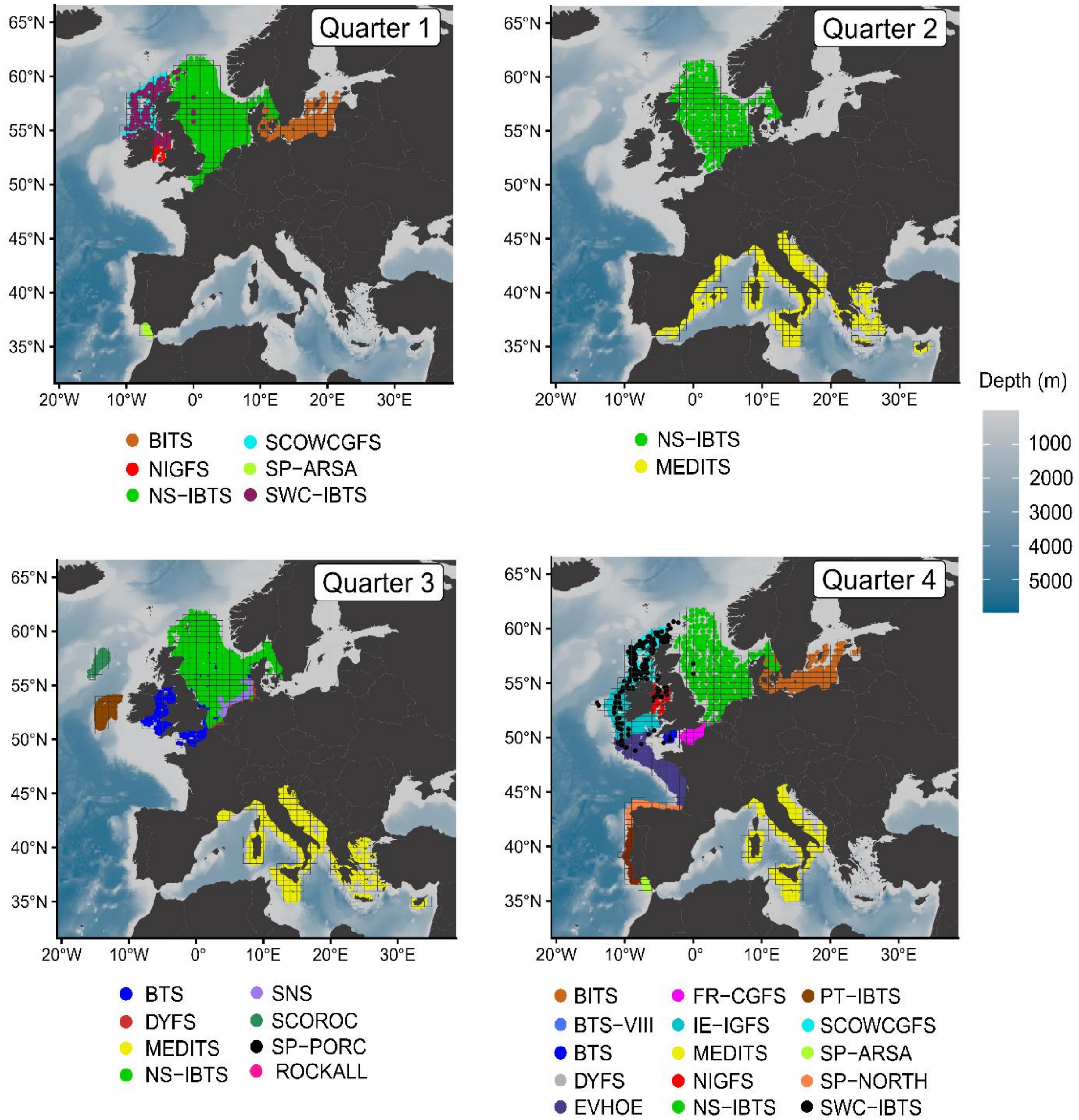

Supplementary Figure 26. **Spatial distribution of demersal bottom trawl programs by quarterly period between 1983 and 2019.** Quarter 1 corresponds to the period January to March, quarter 2 to the period April to June, quarter 3 to the period July to September, quarter 4 to the period October to December). Colors denote the program and the grid cells depict the spatial unit used in this study (1° longitude × 0.5° latitude). Details on different programs can be found in Supplementary Table 6.

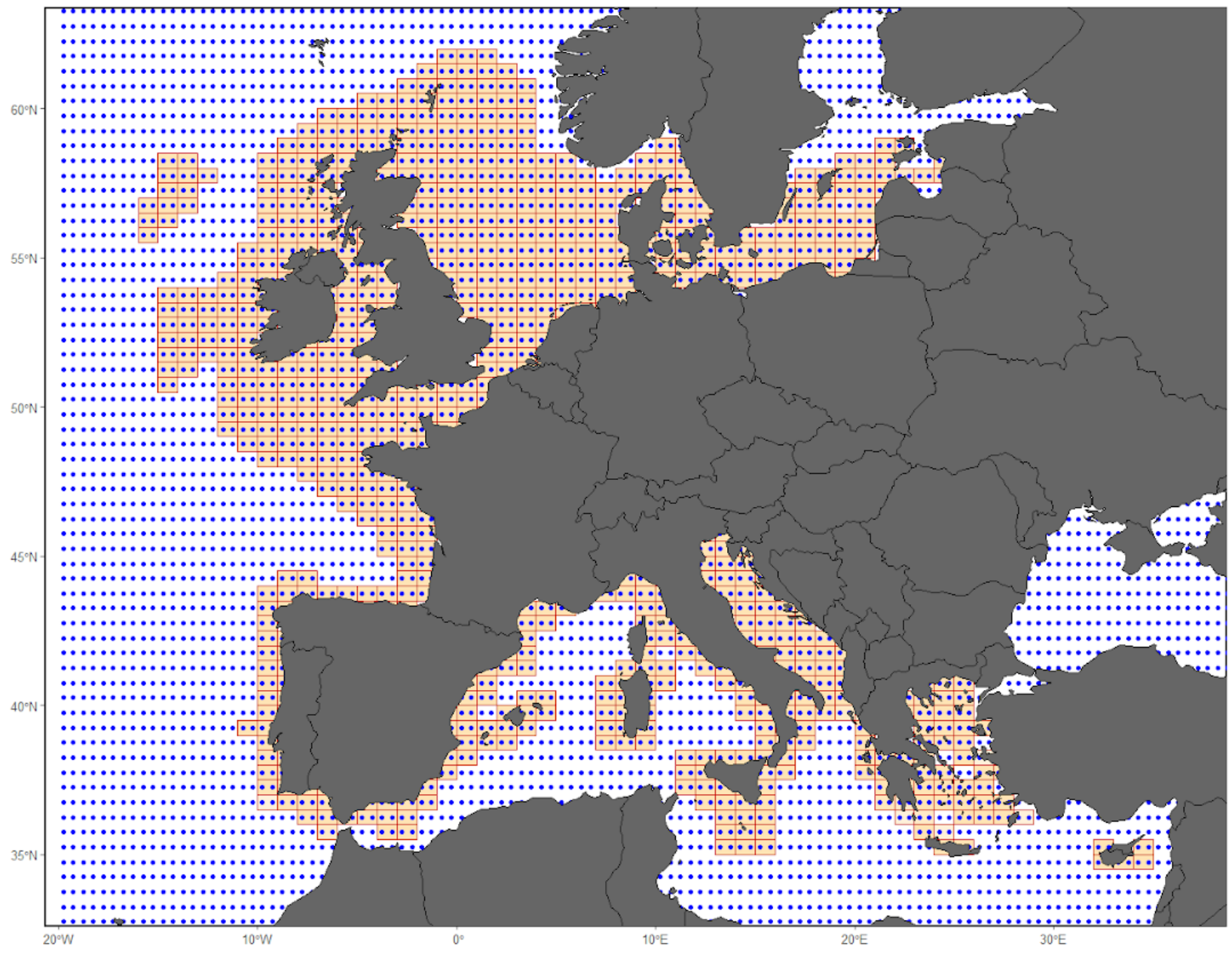

Supplementary Figure 27. **Spatial resolution of fishing effort data and grid cells covering all bottom trawl programs.** Blue dots represents sites associated with fishing data (nominal effort, in kW*day) and orange grid cells depicted by the red grid (1° longitude × 0.5° latitude) represent the spatial unit used for fishing and abundance-based calculations.

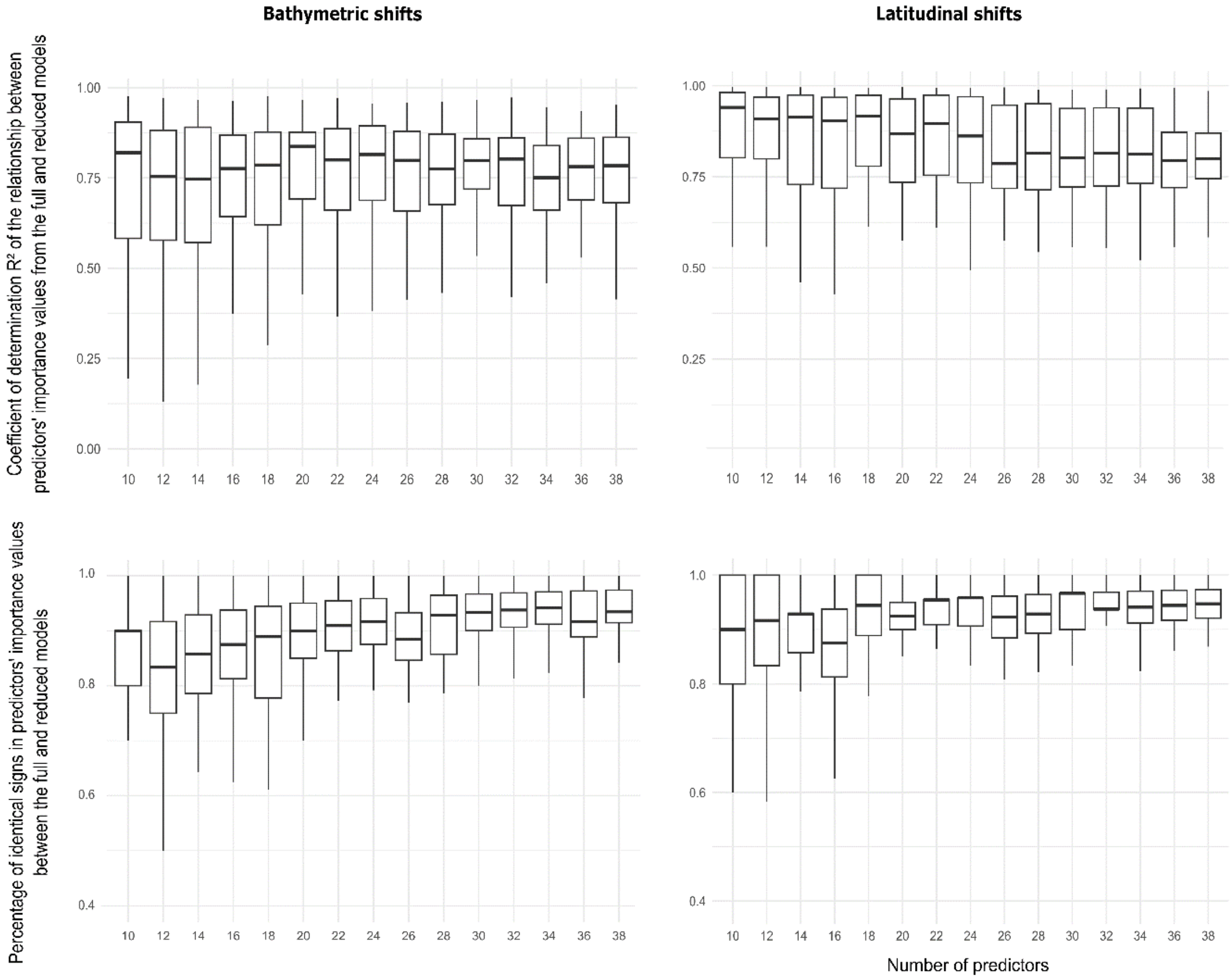

Supplementary Figure 28. **Sensitivity analysis to predictor removal in the XGBoost models developed for bathymetric and latitudinal shifts (left and right, respectively).** The upper panel shows the strength of the relationship between predictor importance values in the full and reduced models, while the lower panel shows the percentage of identical signs in predictor importance values between the full and reduced models.

Supplementary Table 1. **Absolute bathymetric shift velocities of the species exhibiting significant shifts.** Only the populations associated with the 100 most rapid deepening and shallowing shifts are displayed. Blue and red squares correspond to deepening and shallowing shifts, respectively.

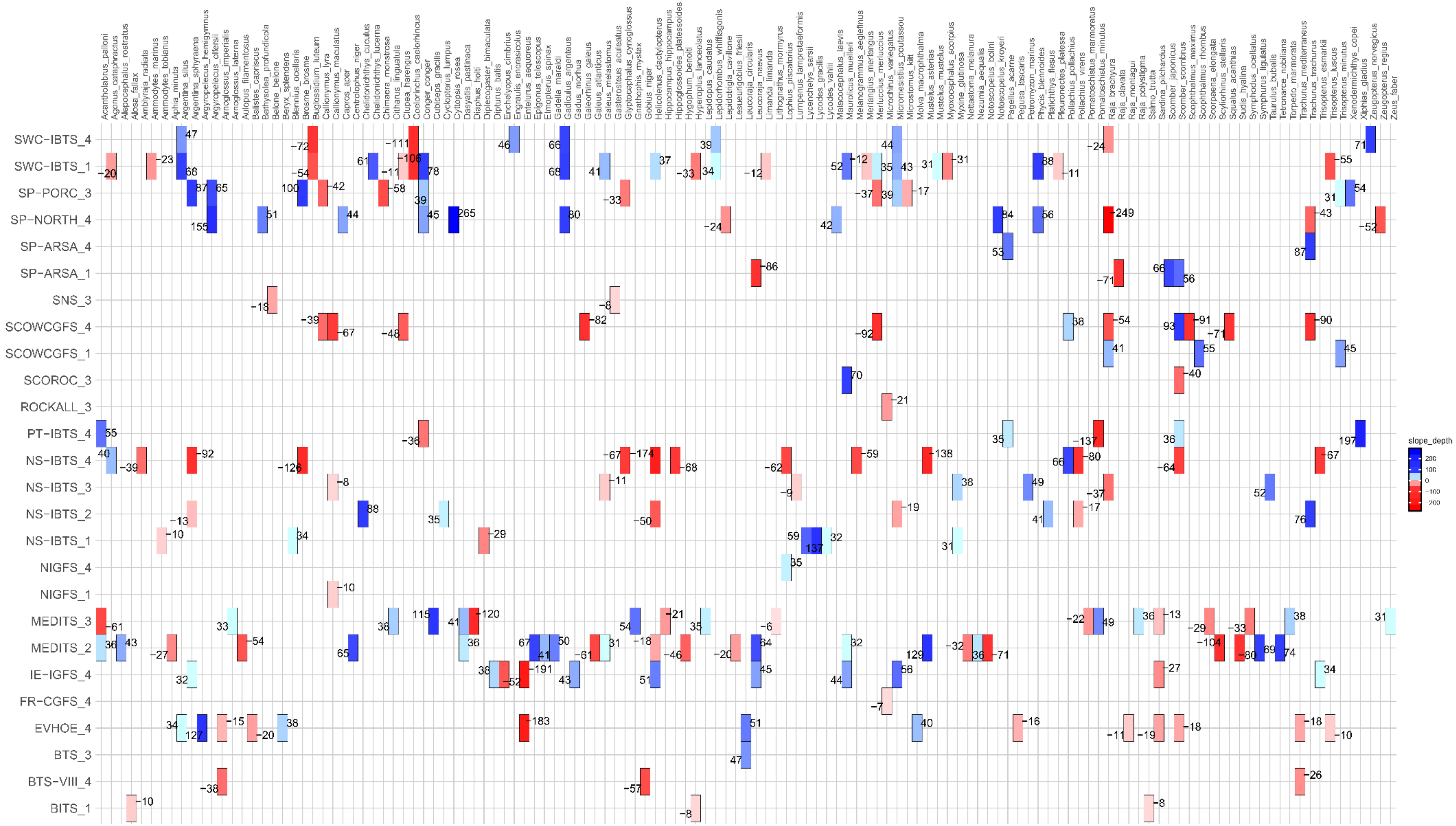

Supplementary Table 2. **Relative bathymetric shift velocity of fish populations (i.e., the percentage change per decade relative to their starting depth, defined as the depth of the population's centroid in the first survey year in which the population was recorded).** Only the populations associated with the 100 most rapid and significant deepening and shallowing shifts are displayed. Blue and red squares correspond to deepening and shallowing shifts, respectively.

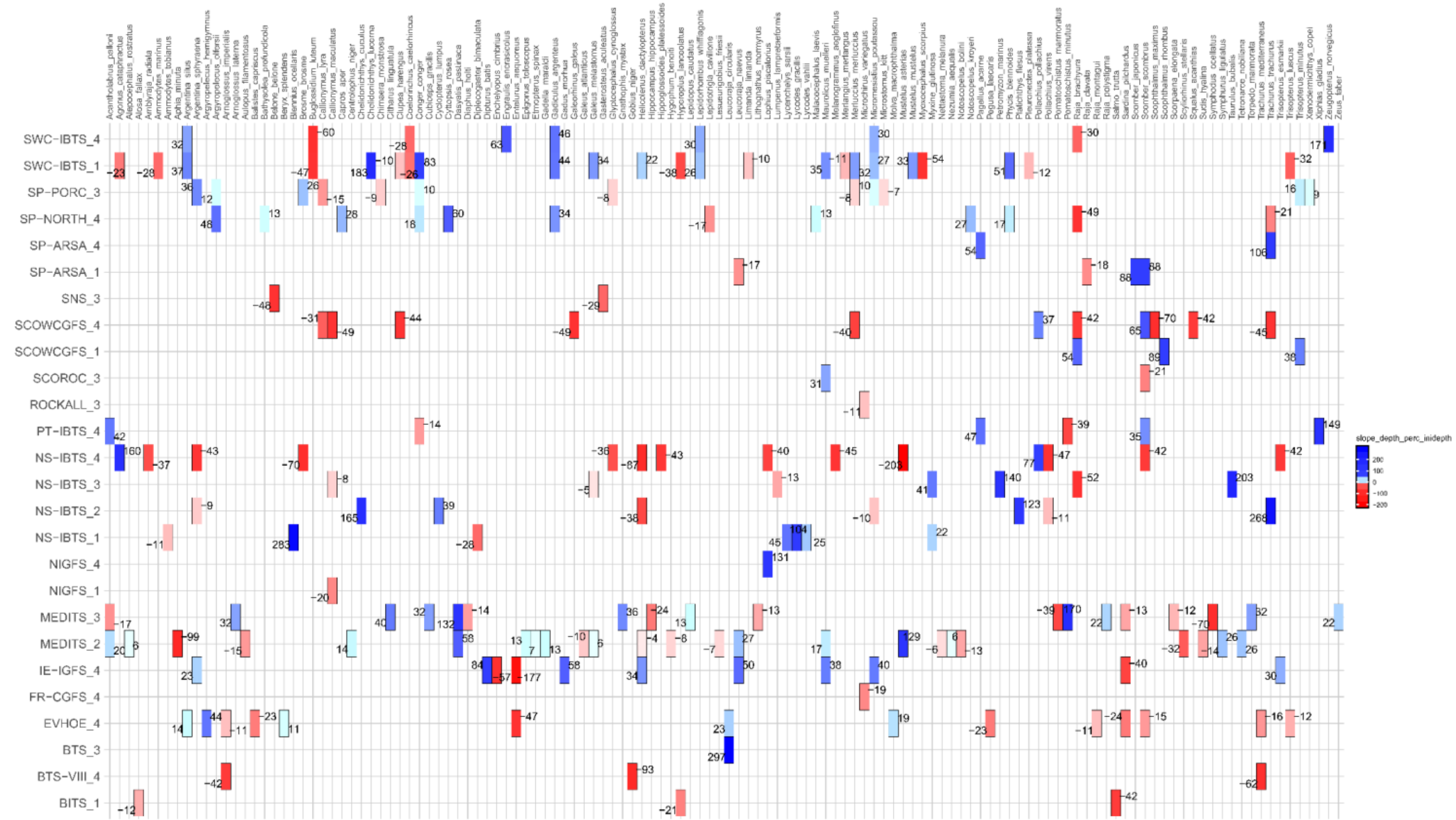

Supplementary Table 3. **Relative bathymetric shift velocity of fish populations (i.e., the percentage change per decade relative to their starting depth range, defined as the difference between the minimum and maximum depth of the population during the three first years in which the population was recorded).** Only the populations associated with the 100 most rapid and significant deepening and shallowing shifts are displayed. Blue and red squares correspond to deepening and shallowing shifts, respectively.

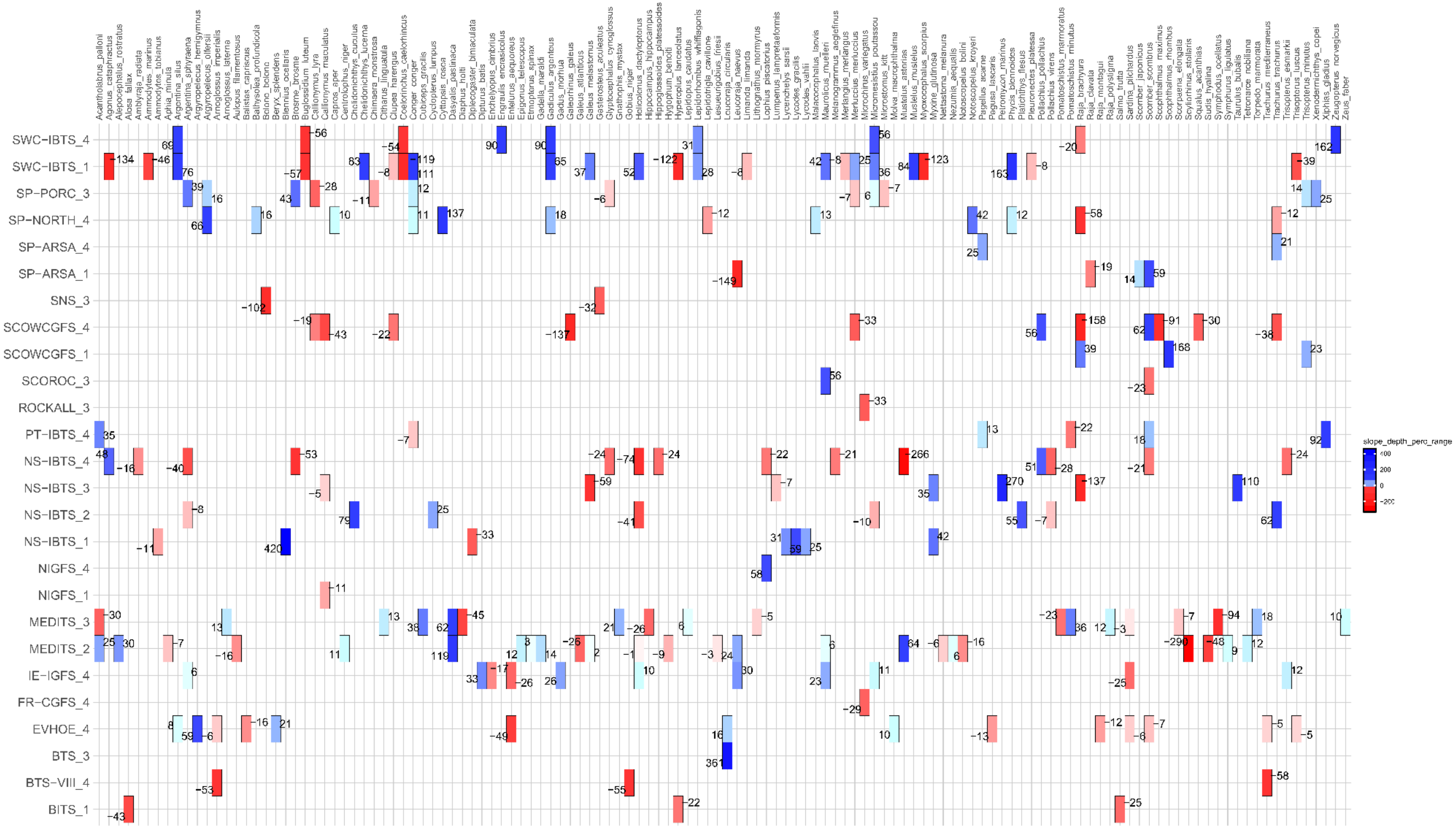

Supplementary Table 4. **Latitudinal shift velocities of the species exhibiting significant shifts.** Only the populations associated with the 100 most rapid northward and southward shifts are displayed.

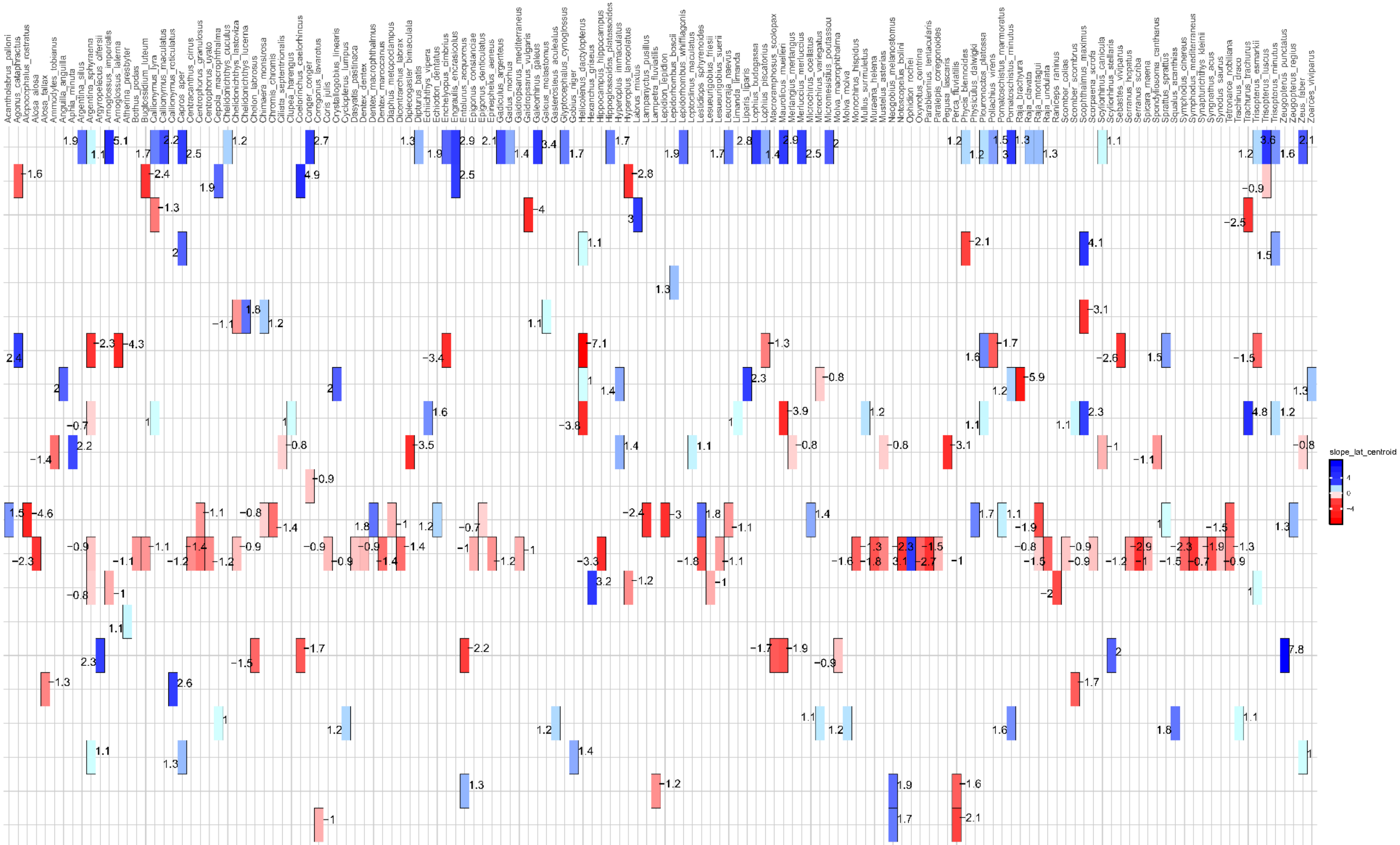

Supplementary Table 5. **Similarity between yearly and quarterly-based environmental data on XGBoost model results.** The first similarity metric (Spearman Footrule Distance) is calculated from the ranks of model predictors (from the higher negative to the higher positive effect) and the second metric (1-Hamming distance) is calculated based on the sign of the effect of model predictors.

	Dependent variable:			
	Bathymetric shift (deepening or shallowing)		Latitudinal shift (northward or southward)	
	ranks	effect sign	ranks	effect sign
Independent variables	75.5%	85%	73.5%	72.5%

Supplementary Table 6. **Bottom trawl programs description.**

Program code	Program name	Area	Period	Quarter	Gear name	Gear type	Latitudinal range (°; [1st decile-last decile])	Bathymetric range (m; [1st decile-last decile])	Reference
BITS	Baltic International Trawl Survey	Baltic Sea	1991-2019	Q1, Q4	CAM; CHP; DT; EGY; ESB; EXP; FOT; GOV; GRT; H20; HAK; LBT; LPT; P20; PEL; SON; TVL; TVS	Bottom trawl	[54.53-57.23]	[20-78]	4
BTS	Beam Trawl Survey	Celtic Sea; English Channel; North Sea	1987-2019	Q3, Q4	BT4A; BT4AI; BT4P; BT4S; BT7; BT8	Beam trawl	[50.28-56.22]	[14-67]	7
BTS-VIII	Beam Trawl Survey – Bay of Biscay	Bay of Biscay	2011-2019	Q4	BT4A	Beam trawl	[45.04-47.60]	[18-86]	7
DYFS	Inshore Beam Trawl Survey	Southern North Sea	2002-2019	Q3, Q4	BT3; BT6	Beam trawl	[51.42-54.13]	[1-15]	7
EVHOE	French Southern Atlantic Bottom trawl Survey	Bay of Biscay and Celtic Sea	1997-2019	Q4	GOV 36/47	Bottom trawl	[44.74-50.90]	[65-216]	1
FR-CGFS	French Channel ground Survey	English Channel	1988-2019	Q4	GOV 36/47	Bottom trawl	[49.45-50.86]	[11-50]	2
IE-IGFS	Irish Groundfish Survey	Ireland Shelf Sea	2003-2019	Q4	GOV 36/47	Bottom trawl	[50.75-55.80]	[56-248]	2
MEDITS	International bottom trawl survey in the Mediterranean	Mediterranean Sea	1994-2018	Q2, Q3, Q4	GOC73	Bottom trawl	[36.70-43.42]	[42-582]	8
NIGFS	Northern Ireland Groundfish Survey	Irish Sea	2009-2019	Q1, Q4	RockHopper ROT	Bottom trawl	[52.72-54.38]	[27-84]	2
NS-IBTS	North Sea International Bottom Trawl Survey	North Sea	1983-2019	Q1, Q2, Q3, Q4	GOV 36/47	Bottom trawl	[53.63-59.73]	[27-133]	3
PT-IBTS	Portuguese International Bottom Trawl Survey	Portugal Shelf Sea	2002-2017	Q4	CAR & NCT	Bottom trawl	[36.90-41.15]	[59-344]	2
ROCKALL	Scottish Rockall Survey (until 2010)	Rockall plateau	1999-2009	Q3	GOV 36/47	Bottom trawl	[56.60-57.75]	[148-195]	2
SCOROC	Scottish Rockall Survey (from 2011)	Scottish plateau	2011-2019	Q3	GOV	Bottom trawl	[56.46-57.81]	[147-300]	2
SCOWCGFS	Scottish West Coast Groundfish Survey	Scottish west coast	2011-2019	Q1, Q4	GOV	Bottom trawl	[55.26-59.38]	[69-184]	2
SNS	Sole Net Survey	Southern North Sea	2002-2018	Q3	BT6	Beam trawl	[52.50-55.37]	[15-36]	7
SP-ARSA	Spanish Gulf of Cadiz Bottom Trawl Survey	Spain	1996-2019	Q1, Q4	BAKa trawl 44/60	Bottom trawl	[36.24-36.95]	[29-502]	6
SP-NORTH	Spanish North Bottom Trawl Survey	North of Spain	2001-2019	Q4	BAKa trawl 44/60	Bottom trawl	[42.50-43.93]	[97-325]	2
SP-PORC	Spanish Porcupine Bottom Trawl Survey	Irish Sea	2001-2019	Q3	PORB	Bottom trawl	[51.54-53.69]	[255-647]	5
SWC-IBTS	Scottish West Coast International Bottom Trawl Survey	Scotland Shelf Sea	1985-2010	Q1, Q4	GOV 36/47	Bottom trawl	[54.08-59.03]	[57-167]	2

BAK: Baka trawl; BT4A: Four m Beam trawl; BT4AI: Four m Beam trawl; BT4P: Four m Beam Trawl; BT4S: Four m Beam Trawl; BT6: Beam trawl 6 meters; BT7: Seven m Beam trawl; BT8: Eight m Beam trawl; CAM: Unspecified Gear; CAR: Bottom trawl FGAV019; CHP: Cod Hopper; DT: Russian bottom trawl; EGY: Unspecified Gear; ESB: Estonian small bottom trawl; EXP: EXPO trawl - winged midwater trawl; FOT: Fotomidwater trawl, GOV: Grande Ouverture Verticale Trawl; GOC73: Grande ouverture et côtés; GRT: Granton trawl; H20: Herring ground trawl (HG20/25); HAK: Hake- 4M; JDT: Jackson Trawl; LBT: Latvian Bottom Trawl; PORB: Porcupine Baka; SON: Sonderborg trawl; TVL: Large TV trawl; TVS: Small TV trawl.

1. ICES. The EVHOE survey (France). ICES Documents. (1997). Available at: https://archimer.ifremer.fr/doc/00036/14707/12013.pdf
2. ICES. Manual of the IBTS North Eastern Atlantic Surveys. Series of ICES Survey Protocols SISP 15 (2017). doi:10.17895/ices.pub.3519
3. ICES. Manual for the International Bottom Trawl Surveys Revision VIII. Series of ICES Survey Protocols SISP 10 - IBTS IX. (2015).
4. https://ices-library.figshare.com/articles/report/SISP_7_-_Manual_for_the_Baltic_International_Trawl_Surveys_BITS_/19050986
5. https://gis.ices.dk/geonetwork/srv/api/records/ce94a257-c8b3-44f7-9fd0-6bd7449ce073
6. http://ices.dk/sites/pub/CM%20Doccuments/2002/D/D0302A.pdf
7. https://ices-library.figshare.com/articles/report/SISP_14_-_Manual_for_the_Offshore_Beam_Trawl_Surveys_WGBEAM_/19051328
8. https://archimer.ifremer.fr/doc/00117/22783/20585.pdf

Supplementary Table 7. **Data Selection rules for the European-scale analysis.**

		Quarter 1	Quarter 2	Quarter 3	Quarter 4
**Northeast Atlantic**	**Bathymetry**	[13 to 166m]	Insufficient data	[7 to 275m] and without the period [1985-1990]	[3 to 214m] and without the period [1985-1990]
	**Latitude**	[51.25°N to 61.25°N]	Insufficient data	[49.25°N to 61.75°N] and without the period [1985-1990]	[43.75°N to 59.75°N] and without the period [1985-1995]
**Mediterranean Sea**	**Bathymetry**	no data	[13 to 692m]	[15 to 650m]	Insufficient data
	**Latitude**	no data	[35.25°N to 44.25°N]	[35.25°N to 45.75°N] and without the period [1990-1995]	Insufficient data

Supplementary Table 8. **Species traits used in the study and commercial status of species, along with the reasoning and references for choosing each trait.**

Species trait	Category	Type	Modalities	unit	Reasoning	References
Length at maturity	Life history	numeric	-	cm	Influences growth rate, metabolism, feeding rate, mobility, position in food web.	Brown *et al.* , 2004; Fisher *et al* ., 2010; Kleiber, 1947; Petchey *et al* ., 2008
Age at maturity	Life history	numeric	-	years	Influences growth rate, speed of maturation and reproduction, population turnover.	Crozier and Hutchings, 2014; King and McFarlane, 2003; Mims and Olden, 2012; Pankhurst and Munday, 2011
Spawning type	Life history	categorical	Bearer/Guarder/Non-guarder	-	Spawning type reflects the amount of parental care, which determines offspring survival and dispersal.	Beukhof *et al* ., 2019; Gross and Sargent, 2015; Smith and Wootton, 1995; Winemiller and Rose, 1992.
Offspring size	Life history	numeric	-	mm	Determines offspring survival and dispersal.	Adams, 1980; Pianka, 1970; Sirot *et al.* , 2015; Ware, 1975
Von Bertalanffy growth coefficient	Life history	numeric	-	year-1	Describes how fast an individual reaches its asymptotic size.	Beukhof *et al* ., 2019
Trophic level	Trophic ecology	numeric	-	-	Influences position within food web, impacts on carbon and nutrient fluxes.	Gravel *et al* ., 2016; Hempson *et al* ., 2018; Huxel and McCann, 1998; Schneider *et al* ., 2016
Feeding mode	Trophic ecology	categorical	Herbivorous, Benthivorous, Planktivorous, Piscivorous, Generalist	-	Type of food consumed and reflects species diet	Beukhof *et al* ., 2019
Habitat type	Habitat use	categorical	Bathydemersal (recoded as 'demersal'), Benthopelagic (recoded as 'pelagic'), Demersal, Mesopelagic (recoded as 'pelagic'), Pelagic, Reef-associated (removed from data set by lack of data)	-	Influences distribution, dispersal, mobility.	Costello *et al.* , 2015; Dencker *et al* ., 2017; Stuart-Smith *et al* ., 2013
Commercial status	-	categorical	Commercial, Minor commercial (recoded as 'commercial'), Of no interest (recoded as 'not commercial'), Highly commercial (recoded as 'commercial'), of potential interest (recoded as 'not commercial'), subsistence fisheries (recoded as 'commercial')	-	A binary proxy of species targeting by fishing	Fishbase (Froese and Pauly, 2000)

Supplementary Table 9. **Description of the environmental layers used to estimate the environmental niches of fish species.**

Environmental parameter	units	primary type	dataset code	primary source	start year	end year	used for	version	layer url
Depth of the seafloor	meters	Satellite (SRTM)	MARSPEC	SRTM30_PLUS V6.0 reference: Becker et al. 2009 URL: http://topex.ucsd.edu/WWW_html/srtm30_plus.html	2009	2009	all species	10	https://www.lifewatch.be/sdmpredictors/MS_bathy_5m_lonlat.tif
Acidity in the ocean	unitless	in situ measurement	Bio-ORACLE	World Ocean Database (2009) Reference: (Boyer et al. 2009) URL: http://www.nodc.noaa.gov/	1910	2007	all species	22	https://bio-oracle.org/data/2.2/Present.Surface.pH.BOv2_2.tif.zip
Mean mass concentration of chlorophyll in sea water at maximum bottom depth	mg/m^3	Model	Bio-ORACLE	Global Ocean Biogeochemistry NON ASSIMILATIVE Hindcast (PISCES) URL: http://marine.copernicus.eu/	2000	2014	demersal species	22	https://bio-oracle.org/data/2.0/Present.Benthic.Max.Depth.Chlorophyll.Mean.tif.zip
Mean mass concentration of chlorophyll in sea water at mean bottom depth	mg/m^3	Model	Bio-ORACLE	Global Ocean Biogeochemistry NON ASSIMILATIVE Hindcast (PISCES) URL: http://marine.copernicus.eu/	2000	2014	pelagic species	22	https://bio-oracle.org/data/2.0/Present.Benthic.Mean.Depth.Chlorophyll.Mean.tif.zip
Mean mole concentration of dissolved molecular oxygen in sea water at maximum bottom depth	micromol/m^3	Model	Bio-ORACLE	Global Ocean Biogeochemistry NON ASSIMILATIVE Hindcast (PISCES) URL: http://marine.copernicus.eu/	2000	2014	demersal species	22	https://bio-oracle.org/data/2.0/Present.Benthic.Max.Depth.Dissolved.oxygen.Mean.tif.zip
Mean mole concentration of dissolved molecular oxygen in sea water at mean bottom depth	micromol/m^3	Model	Bio-ORACLE	Global Ocean Biogeochemistry NON ASSIMILATIVE Hindcast (PISCES) URL: http://marine.copernicus.eu/	2000	2014	pelagic species	22	https://bio-oracle.org/data/2.0/Present.Benthic.Mean.Depth.Dissolved.oxygen.Mean.tif.zip
Mean sea water salinity at the bottom at maximum bottom depth	PSS	Model	Bio-ORACLE	Global Ocean Physics Reanalysis ECMWF ORAP5.0 (1979-2013) URL: http://marine.copernicus.eu/	2000	2014	demersal species	22	https://bio-oracle.org/data/2.0/Present.Benthic.Max.Depth.Salinity.Mean.tif.zip
Mean sea water salinity at the bottom at mean bottom depth	PSS	Model	Bio-ORACLE	Global Ocean Physics Reanalysis ECMWF ORAP5.0 (1979-2013) URL: http://marine.copernicus.eu/	2000	2014	pelagic species	22	https://bio-oracle.org/data/2.0/Present.Benthic.Mean.Depth.Salinity.Mean.tif.zip
Mean sea water temperature at the bottom at maximum bottom depth	degrees Celcius	Model	Bio-ORACLE	Global Ocean Physics Reanalysis ECMWF ORAP5.0 (1979-2013) URL: http://marine.copernicus.eu/	2000	2014	demersal species	22	https://bio-oracle.org/data/2.0/Present.Benthic.Max.Depth.Temperature.Mean.tif.zip
Mean sea water temperature at the bottom at mean bottom depth	degrees Celcius	Model	Bio-ORACLE	Global Ocean Physics Reanalysis ECMWF ORAP5.0 (1979-2013) URL: http://marine.copernicus.eu/	2000	2014	pelagic species	22	https://bio-oracle.org/data/2.0/Present.Benthic.Mean.Depth.Temperature.Mean.tif.zip
