# Supplementary material for: Marine fish ranges are expanding and shifting deeper across the European continental shelf
Source: Nat Commun. 2026 Jul 15;17:8702. doi: 10.1038/s41467-026-74371-8 (PMC13490448; doi:10.1038/s41467-026-74371-8)
Supplement: Supplementary file 2 [file 41467_2026_74371_MOESM5_ESM.pdf]

nature portfolio

Corresponding author(s): Arnaud Auber

Last updated by author(s): Apr 09, 2026

Reporting Summary

Nature Portfolio wishes to improve the reproducibility of the work that we publish. This form provides structure for consistency and transparency in reporting. For further information on Nature Portfolio policies, see our Editorial Policies and the Editorial Policy Checklist.

Statistics

For all statistical analyses, confirm that the following items are present in the figure legend, table legend, main text, or Methods section.

n/a	Confirmed	
☐	☒	The exact sample size (*n*) for each experimental group/condition, given as a discrete number and unit of measurement
☐	☒	A statement on whether measurements were taken from distinct samples or whether the same sample was measured repeatedly
☐	☒	The statistical test(s) used AND whether they are one- or two-sided<br>*Only common tests should be described solely by name; describe more complex techniques in the Methods section.*
☐	☒	A description of all covariates tested
☐	☒	A description of any assumptions or corrections, such as tests of normality and adjustment for multiple comparisons
☐	☒	A full description of the statistical parameters including central tendency (e.g. means) or other basic estimates (e.g. regression coefficient) AND variation (e.g. standard deviation) or associated estimates of uncertainty (e.g. confidence intervals)
☐	☒	For null hypothesis testing, the test statistic (e.g. *F*, *t*, *r*) with confidence intervals, effect sizes, degrees of freedom and *P* value noted<br>*Give P values as exact values whenever suitable.*
☒	☐	For Bayesian analysis, information on the choice of priors and Markov chain Monte Carlo settings
☒	☐	For hierarchical and complex designs, identification of the appropriate level for tests and full reporting of outcomes
☐	☒	Estimates of effect sizes (e.g. Cohen's *d*, Pearson's *r*), indicating how they were calculated

Our web collection on statistics for biologists contains articles on many of the points above.

Software and code

Policy information about availability of computer code

Data collection	No software was used to collect the data
Data analysis	All statistical analyses were performed under the R environment (R Core Team, 2024)

For manuscripts utilizing custom algorithms or software that are central to the research but not yet described in published literature, software must be made available to editors and reviewers. We strongly encourage code deposition in a community repository (e.g. GitHub). See the Nature Portfolio guidelines for submitting code & software for further information.

Data

Policy information about availability of data

All manuscripts must include a data availability statement. This statement should provide the following information, where applicable:

- Accession codes, unique identifiers, or web links for publicly available datasets
- A description of any restrictions on data availability
- For clinical datasets or third party data, please ensure that the statement adheres to our policy

Abundance data are publicly available for the North East Atlantic (DATRAS database; https://datras.ices.dk/Data_products/Download/Download_Data_public.aspx). Mediterranean data (MEDITS database) are available upon request to Maritime Affairs and Fisheries (MARE DATACOLLECTIONFRAMEWORK : https://dcf.ec.europa.eu/guidelines_en). Some of the datasets used here are also available in the SEANOE public repository : French NS-IBTSQ1: doi.org/10.17882/100023, EVHOE: doi.org/10.17882/80041, and French MEDITS: doi.org/10.17882/99052). Species traits were collected by extracting traits values from Coulon et al. (2023) openly available on https://doi.org/10.6084/m9.figshare.19833304.v1. It is a combination of the publicly available PANGAEA database (https://

doi.pangaea.de/10.1594/PANGAEA.900866), Fishbase information (https://www.fishbase.se/search.php), and inference based on the FISHLIFE project (https://esajournals.onlinelibrary.wiley.com/doi/10.1002/eap.1606). Commercial status of fish species were extracted from Fishbase (https://www.fishbase.se/search.php). Global occurrence data were extracted occurrence data from the OBIS (https://obis.org/) and GBIF platforms (https://www.gbif.org/), thanks to the occurrence function of the robis R package and the occ_download function of the rgbif R package, respectively. Fishing effort data were obtained from Rousseau et al., 2024: https://metadata.imas.utas.edu.au/geonetwork/srv/eng/catalog.search#/metadata/1241a51d-c8c2-4432-aa68-3d2bae142794; doi:10.25959/MNGY-0Q43). Environmental data are all publicly available on Copernicus website (https://data.marine.copernicus.eu/products). Depth values are available from the General Bathymetric Chart of the Oceans database at the trawl station scale (GEBCO 2022: https://www.bodc.ac.uk/data/published_data_library/catalogue/10.5285/e0f0bb80-ab44-2739-e053-6c86abc0289c). All R codes and data used to produce all the figures and tables of the present study are available at https://doi.org/10.6084/m9.figshare.28081469.

Research involving human participants, their data, or biological material

Policy information about studies with human participants or human data. See also policy information about sex, gender (identity/presentation), and sexual orientation and race, ethnicity and racism.

Reporting on sex and gender	No sex- or gender-related data were used in this study.
Reporting on race, ethnicity, or other socially relevant groupings	No race, ethnicity, socially relevant groupings data were used in this study.
Population characteristics	No human-related data were used in this study.
Recruitment	No participant were needed for this study.
Ethics oversight	International Council for the Exploration of the Sea and MEDITS working group

Note that full information on the approval of the study protocol must also be provided in the manuscript.

Field-specific reporting

Please select the one below that is the best fit for your research. If you are not sure, read the appropriate sections before making your selection.

☐ Life sciences ☐ Behavioural & social sciences ☒ Ecological, evolutionary & environmental sciences

For a reference copy of the document with all sections, see nature.com/documents/nr-reporting-summary-flat.pdf

Ecological, evolutionary & environmental sciences study design

All studies must disclose on these points even when the disclosure is negative.

Study description	This study quantifies the extent to which marine fish populations have relocated along bathymetric and latitudinal gradients, and examines how these relocations are driven by the interplay between environmental factors, fishing pressure, safety margins, and species traits in the Northeast Atlantic and the Mediterranean Sea. To this end, we use a comprehensive dataset comprising 92,961 bottom trawl samples collected every year across the European continental shelf, covering 556 fish species over the past four decades. We show that deepening is the predominant response, with 60% of fish populations experiencing an increase in their mean depth. Contrary to expectations, latitudinal shifts are balanced, with 49% of populations shifting northward and 51% southward.
Research sample	Each of the 92,961 bottom trawl samples corresponds to a catch of several fish species. For each species and each haul, fish individuals were counted on vessels in sea across the European continental shelf over the past four decades. The total data set contains 556 fish species. Abundance data for are publicly available for the North East Atlantic (DATRAS database; https://www.ices.dk/data/data-portals/Pages/DATRAS.aspx). Mediterranean data (MEDITS database) are available upon request to Maritime Affairs and Fisheries (MARE DATACOLLECTIONFRAMEWORK : https://dcf.ec.europa.eu/guidelines_en).
Sampling strategy	Survey locations in the Northeast Atlantic, monitored annually, are organized into a grid of 1° longitude by 0.5° latitude, following the International Council for the Exploration of the Sea (ICES) standards. In the Mediterranean Sea, annual hauls follow a depth-stratified sampling scheme, with positions randomly selected within each stratum. The number of sampling sites per stratum is proportional to its total area. Unless obstructed by technical issues or previous gear damage, hauls are conducted at the same coordinates each year. To ensure consistent spatial resolution across the entire study area, the Atlantic ICES grid was extended to include the Mediterranean. Each bottom trawl sample was performed during daylight hours at an average speed of 3 knots and an average duration of 34 minutes.
Data collection	Abundance data are formatted by survey leaders and then uploaded to the data repository (managed by the International Council for the Exploration of the Sea) following a standardized format (Northeast Atlantic : https://www.ices.dk/data/data-portals/Pages/DATRAS_format_description.aspx and Mediterranean Sea: https://www.sibm.it/MEDITS%202011/new/RoME_April_2019/RoME%201.4%20User%20Manual%20-%202019.pdf).
Timing and spatial scale	Survey start dates vary depending on the specific survey (see Supplementary Table 8). The combined dataset spans from 1983 to 2019 in the Northeast Atlantic and Mediterranean Sea (Shelf waters). Surveys are conducted annually to assess fish stocks, providing the data necessary to define catch quotas for the following year. The spatial resolution follows a 1° longitude by 0.5° latitude grid, in accordance with International Council for the Exploration of the Sea (ICES) standards.
Data exclusions	First, survey time series for which the total number of hauls over the entire period did not exceed 300 were excluded to prevent

	wrong assessment of range shifts. At the survey time-series scale only, to minimize potential biases from undersampled species, we included only those present in the abundance dataset for at least five years. For this European scale analysis, because not all survey time-series were conducted every year, we aggregated years into periods of five years (i.e. 1980-1985, 1985-1990, etc.) and further excluded species that were not present in at least four periods to compute reliable shift velocity values. Within each of the two regions (Northeast Atlantic and Mediterranean Sea), species occurring in fewer than 50 hauls were excluded. Finally, we excluded data in grid cells located in extremely deep, shallow, north and south areas that were not sampled during most of the periods (see selection rules in Supplementary Table 9).
Reproducibility	R codes were computed in a way to be reproducible by users. Explanations are within the provided code. All attemps to reproduce results are successful.
Randomization	No randomization. Fish catches were directly attributed to their respective hauls.
Blinding	No blinding in this study because not necessary for these survey protocols.

Did the study involve field work? ☒ Yes ☐ No

Field work, collection and transport

Field conditions	Sampling by bottom traw survey in varying weather conditions
Location	Sampling in the Northeast Atlantic and Mediterranean Sea (continental shelf).
Access & import/export	For each survey, every year, a trawling permit was obtained from local authorities to collect the samples.These permits are standard requests made by most European countries whenever their vessels need to operate in foreign waters, and they are submitted regularly each year to each of the embassies. The scientific surveys conducted here are coordinated by the International Council for the Exploration of the Sea ICES : info@ices.dk, and the Mediterranean International Trawl Survey MEDITS.
Disturbance	No disturbance

Reporting for specific materials, systems and methods

We require information from authors about some types of materials, experimental systems and methods used in many studies. Here, indicate whether each material, system or method listed is relevant to your study. If you are not sure if a list item applies to your research, read the appropriate section before selecting a response.

Materials & experimental systems

n/a	Involved in the study
☒	☐ Antibodies
☒	☐ Eukaryotic cell lines
☒	☐ Palaeontology and archaeology
☐	☒ Animals and other organisms
☒	☐ Clinical data
☒	☐ Dual use research of concern
☒	☐ Plants

Methods

n/a	Involved in the study
☒	☐ ChIP-seq
☒	☐ Flow cytometry
☒	☐ MRI-based neuroimaging

Animals and other research organisms

Policy information about studies involving animals; ARRIVE guidelines recommended for reporting animal research, and Sex and Gender in Research

Laboratory animals	This study didn't involve laboratory animal
Wild animals	The data were collected via bottom trawl hauls during annual scientific surveys. These European programs are designed to assess the biomass of exploited fish stocks, providing the scientific basis for setting annual catch quotas. All trawling operations are conducted under specific research permits, which are requested and obtained annually by each participating nation from the relevant local authorities.These permits are standard requests made by most European countries whenever their vessels need to operate in foreign waters, and they are submitted regularly each year to each of the embassies. The scientific surveys conducted here are coordinated by the International Council for the Exploration of the Sea ICES : info@ices.dk, and the Mediterranean International Trawl Survey MEDITS.
Reporting on sex	Sex wasn't considered in this study
Field-collected samples	No laboratory work

Ethics oversight	Guidance on the study protocol is provided by the International Council for the Exploration of the Sea (ICES) and MEDITS working group. Trawling authorizations are obtained annually by each participating nation from the relevant local authorities. These permits are standard requests made by most European countries whenever their vessels need to operate in foreign waters, and they are submitted regularly each year to each of the embassies. The scientific surveys conducted here are coordinated by the International Council for the Exploration of the Sea ICES : info@ices.dk, and the Mediterranean International Trawl Survey MEDITS.

Note that full information on the approval of the study protocol must also be provided in the manuscript.

Plants

Seed stocks	No seed stocks were used in this study.
Novel plant genotypes	No novel plant varieties were involved in this research.
Authentication	No seed stocks or novel plant species were involved in this study. Therefore, no authentication was required.
