# Supplementary material for: Marine fish ranges are expanding and shifting deeper across the European continental shelf
Source: Nat Commun. 2026 Jul 15;17:8702. doi: 10.1038/s41467-026-74371-8 (PMC13490448; doi:10.1038/s41467-026-74371-8)
Supplement: Supplementary file 3 [file 41467_2026_74371_MOESM6_ESM.pdf]

nature portfolio

Peer Review File

Marine fish ranges are expanding and shifting deeper across the European continental shelf

Corresponding Author: Dr Arnaud Auber

This file contains all reviewer reports in order by version, followed by all author rebuttals in order by version.

Version 0:

Reviewer comments:

Reviewer #1

(Remarks to the Author)
This paper presents a comprehensive analysis investigating depth and latitudinal shifts in fish populations across Europe. A majority of species display deepening ranges over time, with a 50-50 split of latitudinal shifts (species seem equally likely to move north or south). These shifts are then explored in the context of environmental change, species specific environmental niche and biological traits.

The analyses are advanced and appropriate to deal with complex and disparate data sources. Showing clear and sometimes unexpected trends in fish populations.

I have some general points as well as some specific comments which I will outline below.

Throughout the manuscript surveys are referenced, which I know is the accepted convention. However, I think it may be clearer (especially in some parts of the methods e.g. line 545) to refer to survey time-series or programs which then lets you refer to individual surveys (i.e. a single instance within a survey time-series).

Throughout the paper, mean and median shift speeds are presented which is very helpful, but I wonder whether % change may also have been useful to give a feel for how big these shifts are in comparison to the starting depth.

The overall mean speed of depth shift is quite high (19 +/- 27 m.decade). Whilst I'm sure it is correct I wonder if it is masking regional differences. For example, a 19 m/decade shift would result in almost 80 m of deepening over the 40 years of data analysed. As we're dealing with largely demersal species (or at least the bottom gears limit the sampling depth to the maximum available) an 80 m deepening should presumably result in species being found on sea floor ~ 80 m deeper. However, this would not be possible in an environment like the North sea, with an average depth of 100 m. So far as I can see, regional (NeA & MedS) mean & median speeds are not presented, just the corresponding supplementary figures. But as a lot of the surveys in the NeA are on the shelf edge, this may also mask the pattern in the North Sea. This large environmental 'barrier' to deepening is not discussed, although there is good opportunity to do so alongside the discussion around limitations of latitudinal shifts due to the strait of Gibraltar (Lines 261 – 263).

There is no discussion or methods text devoted to the species included in the analyses. There is some explanation of exclusions based on number of observations (line 547), but it is unclear whether this refers to all analyses (i.e. the survey scale) or just the seasonal scale approach. There are likely to be varying gear selectivity issues around many of the species analysed. It is clear from some of the supplementary figures and explanations of the species traits that some species were pelagic for example (e.g. Clupea harengus, Scomer scombrus). These are likely to be poorly sampled as the bottom gear only fishes optimally when being towed on the sea bed. This means that observations and abundances of pelagic species are likely to be sporadic, but also mor importantly, that depth information cannot be assigned to them as they have potentially been captured as gear is being hauled, rather than the underlying depth influencing their habitat/capture location. Similarly, depending on the gear type, mesh size etc. some small (or large) species may be very poorly/sporadically sampled.

Line-by-line comments:

Line 12: I know that the range here is representative of all metapopulations that display a shift (60%) and the % of all significant shifts that displayed a deepening trend (75%). These are two different concepts and it's unclear to the reader why a range is presented. I would just pick one or the other to present.

Line 39: 562 fish species mentioned in text, but 613 present in your species trait dataset

Line 74: change or to of

Line 101: 'associated with movements toward greater depths rather than toward the surface' This terminology seems misleading - this is an analysis of bottom/bean trawl survey data, demersal species changing depth distribution would likely reflect a geographic shift along with depth, but pelagic species could shift within the water column (although I don't think thew would be adequately sampled to draw conclusions).

Line 103-104 & lines 232-234: You mention here that the majority of metapopulations (69%) were characterized by an increase in abundance – was this also true for commercially exploited species? From your other analyses I think it should be easy to isolate these and may add nicely to your discussion around fishing pressure if it shows abundance of commercial species has displayed no increase. Or indeed if they have increased, potentially as a result of common fisheries policy.

Lines 234 – 238:These two sentences seem to say opposing things. Firstly that the higher fishing pressure in shallower areas may have led to the shift in centroids (presumably deeper as this is the general trend reported). But the following sentence then says that the common fisheries policy has reduced fish pressure which has also led to deepening. Surely a reduction in the pressure outlined in the first sentence would result in shallowing as these populations re-colonise historical habitat as pressure is reduced (not accounting for environmental conditions).

Line 250 (and discussion generally): lots of the discussion text is in reference to improved fishery management, but surely this would only apply to those species impacted by fisheries (either as target or bycatch species)? From the supplementary figures 13 & 14 it looks like more than half the species are not correlated with fishing effort.

Line 257: 'effective fishery management is highly likely to restore exploited stocks, promoting their ability to fill these niches.' As above, if fishing activity has resulted in deepening for some species, this would result in shallowing?

Line 261-263: see comment above about depth constraints across which is likely as big (or bogger) of a factor as depth shifts seem to be more prevalent.

Lines 270 – 274: I completely agree, something like a food-web modelling approach would compliment this study very well.

Line 521: what are the max, min, mean values for speed and time?

Line 545: individual surveys or survey time series with fewer than 300 hauls over multiple years in a single quarter?

Lines 554 – 555: You removed species not present in < 50 hauls, was this done for survey-level analyses as well?

Supplementary material:
Figure 6: closing square brackets are facing the wrong way on the y-axis.

Table 3 & 4: I can't imagine that some of these species are particularly common in standard surveys.
Is it possible that some of these large shifts are driven by low sampling and that the capture of many of these species is dependent on gear selectivity which is likely not optimized for rare/small/oddly shaped species. Pelagic species are likely sporadic in catches (e.g. engraulids, myctophids) if bottom trawl time-series were used.
Some species also have both deepening and shallowing trends within the same geographical area (e.g. Coelorinchus caelorhincus). Scomber scombrus (pelagic - likely not sampled well with these approaches.). But I can't see this discussed anywhere. It possibly suggests that sampling biases over-ride any environmental/biological signals.
Grouping the NeA into one category likely smooths over lots of differences between shelf and shelf-edge habitats. Do common species between these habitats show the same trends?

(Remarks on code availability)

Reviewer #2

(Remarks to the Author)
Summary
This paper presents a large-scale analysis of fish distribution shifts across European seas using decades of standardized trawl survey data. The study finds that deepening is the dominant response, while latitudinal shifts appear more balanced. The work is interesting, well-written, and relevant for both ecology and fisheries management.

However, the novelty is somewhat limited as previous studies have already shown many of the patterns found here. For example: Chaikin et al. 2021 (https://doi.org/10.1111/geb.13414) demonstrated widespread deepening of fish populations in the Mediterranean. Gordo-Vilaseca et al. 2023 (https://peerj.com/articles/15801/) demonstrated some northward shifts in the

North, Barents, and Norwegian seas. Supplementary Tables 1 and 2 do cover such results, so it's not as though they're ignored here.

What's perhaps most novel in this paper is the analysis tying shifts to explanatory variables including environmental changes and fishing. Still, I'm not sure there's enough novelty here to justify publication in Nature Communications rather than a top quality fisheries journal - for example: Fish and Fisheries or ICES JMS.

Main suggestions:
1. I was wondering if the deepening pattern might partly come from benthic fish moving farther offshore, since depth and distance from the coast are usually linked. It could also just reflect relative declines in nearshore abundance (for example, from nearshore fishing pressure) rather than actual movement. In that case, the center of gravity would still shift deeper even if fish didn't really move. It might be worth the authors mentioning this possibility and suggesting some methods to test it as sensitivity analysis.
2. One potentially important omission is the role of geographic barriers in constraining latitudinal shifts. Even though the analysis separates the Northeast Atlantic and the Mediterranean, there are still obvious land barriers within those regions. These barriers likely restrict species from showing clear poleward movements, even under strong warming. This contrasts with studies from more continuous coastlines (like the U.S. east coast but see Gulf of Mexico patterns in Pinsky et al. 2013), where poleward shifts are more detectable because alongshore connectivity is higher
3. The whole idea of deepening and poleward shifts is usually based on the assumption that temperature is the key driver — with global warming pushing species deeper or toward the poles to stay within their preferred thermal range. But in this study, the models suggest that bottom temperature has almost no effect on deepening. That means the fundamental hypothesis driving "deepening" and "poleward" shifts seem doesn't really hold here, which I think the authors should acknowledge more clearly.
4. One limitation is the survey coverage. The standardized surveys were designed around the species' original distributions, so if species have shifted outside that coverage, the surveys may not capture them. This is a known issue for many highly mobile or high-seas species. I'm not sure how relevant it is here, but it might be worth mentioning.

Minor suggestions:
"meta-population" doesn't seem like the right term here as it already has a specific meaning (see Hanski and Gilpin) that is not what's meant here. How about "stock" or simply "population"?
Line 25–27: the logic feels inconsistent — if fisheries are "overlooked," how can most studies also be focused on commercially important species?
Supplementary Tables 1 and 2: not sure if the shading is supposed to be red or yellow. Also, what about the Gordo-Vilaseca et al. 2023 paper mentioned above?
Supplementary Figure 12: some of the y-axis labels fall outside the panel boundary.

(Remarks on code availability)

Reviewer #3

(Remarks to the Author)
I co-reviewed this manuscript with one of the reviewers who provided the listed reports. This is part of the Nature Communications initiative to facilitate training in peer review and to provide appropriate recognition for Early Career Researchers who co-review manuscripts.

(Remarks on code availability)

Reviewer #4

(Remarks to the Author)
Scientific findings
This study highlights that deepening is the most common response in fish populations in the Atlantic and the Mediterranean Sea. These finding challenges traditional expectations and previous research that prioritized latitudinal shifts.

The extensive database provides a comprehensive and detailed view of shift trends at the metapopulation level, something that few studies address on such a scale. It presents a multidimensional and multifactorial perspective, offering a realistic view of the phenomenon, which provides a more complete perspective on the effects of climate change.

However, caution is needed regarding the role of fishing and management. The authors state that the reduction in fishing pressure due to management policies has facilitated the spatial expansion of species, introducing a management dimension that is not always considered in other studies focused solely on ecological responses to climate change. However, it should be noted that more evidence is needed from other regions, as this could be misinterpreted and used as an argument for misuse or overexploitation of resources. I urge the authors to develop a sentence with the limitations and cautions of this statement.

While current measures have contributed to reducing pressure on fishery resources, the distribution of species toward

deeper areas is a significant finding.

Methods
Overall, the statistical methods used in this study are appropriate for addressing the research questions. The use of machine learning models such as XGBoost is appropriate given their ability to handle complex relationships and high-dimensional data. However, the use of these models requires addressing overfitting, an issue that should be described in the methodology.
On the other hand, classical statistical analyses (e.g., Wilcoxon tests) were also used to detect changes in displacement rates, a method suitable for nonparametric comparisons in time-trend data. The description of mean speeds and confidence intervals for displacements indicates an appropriate use of descriptive and inferential statistics.

Recommendations
1. Validation of machine learning models: Conduct sensitivity analyses to assess how changes in predictor variables affect the results, especially in displacement estimates.
2. Explore traditional complementary methods such as generalized linear models (GLMs) or mixed models to assess the consistency of the trends detected by machine learning, especially in temporal and spatial trend analyses.

Discussion
Although they are mentioned in the references section, developing a statement using three-dimensional species distribution models, given that depth movements are predominant, it is essential to include the vertical dimension (depth) in marine conservation prediction and planning models to better capture the spatial dynamics of species.

Explicitly mention or discuss that understanding changes in species distribution requires long-term, standardized, and large-scale data. It is crucial to maintain and expand these monitoring programs to detect emerging changes and evaluate the effectiveness of implemented policies.
Discuss the prioritization of refuge areas in environments with high habitat diversity. Bathymetric heterogeneity provides refuges for metapopulations, so protecting areas with diverse environments and depths can strengthen the resilience of marine communities to climate change.

Language
No errors were observed in the grammatical structure, only some punctuation details and the elimination of text containing redundancies or repeated ideas. Most of the redundancies are related to the deepening trend in species movements and the role of bathymetry. The reiteration that movements toward greater depths predominate, and that these movements also occur in northerly and southerly directions, appears several times, reinforcing the general trend, but which can be considered redundant.

Figures
The figures are appropriately related to the content of the text and the data presented. The figures effectively represent the study's findings.

References
The bibliographic references are adequately cited. According to the reference list, they include publications ranging from 2003 to 2024, indicating recent research. Some references from 2023 and 2024 reflect updated literature on climate change and marine species distribution.

Minor comments:
Line 21. Body of research by “Body of evidence”
Line 31. How does it increase the biodiversity metric?
Line 53: I suggest replacing “spatial pixels” (image element) with “grid cell” (discrete unit within a grid)
Line 58. Center of gravity or Centroid of geographic distribution?
Line 79 “Because the latitudinal range of each metapopulation within a given survey may therefore represent only a fraction of its full global distribution, our ability to detect latitudinal shifts could be limited.” The sentence is correct, although it sounds a bit long. You can improve the punctuation if you wish, but it's not mandatory.
Line 100: “More specifically, this spatial expansion of metapopulations has mainly been associated with movements toward greater depths rather than toward the surface, as well as toward the north or south". The mention of movements towards greater depths and in north and south directions is repeated in different parts of the analysis, reinforcing the trend without providing additional substantive information.
Line 122. Both species???
Line 140. “NS” is not necessary.
Line 161. I suggest: extreme gradient boosting (XGBoost)-based machine learning method
Line 208:“Using an extensive dataset from 21 bottom trawl surveys conducted across European continental shelf waters over the last four decades, we identify a strong discrepancy between depth and latitudinal shifts in marine fish species.”
In other sections, it is repeated that deep displacements are more frequent or significant, which can be considered a reiteration.
Line 217. Geographic barrier, like which one(s)?
Line 217: The idea of species moving deeper and its relationship to environmental change is repeated several times in the same paragraph and throughout the document, reinforcing the trend without providing any new information.
Line 219 “The mentions the “deepening” trend in several places and explains again the diversity of environments created by bathymetry, reiterating the same idea redundantly.

Line 257. Replace “geographical niche” by ecological niche.
Line 264. The idea that these environmental mosaics are “critical” is repeated in several parts of the text, highlighting that the diversity of environmental conditions and their direction are key requirements for understanding spatial movements.
Line 273. Use the concept of ‘ecological niche conservatism’ in this section.
Lines 287 to 290: Sentence repeated with paragraphs 273 to 276.
Line 289: Delete “more” and “future.” Mitigation is now!
Line 509: Incomplete citation: McFadden, D. (1973) Conditional Logit Analysis of Qualitative Choice Behavior. In: Zarembka, P., Ed., Frontiers in Econometrics, Academic Press, 105-142.
Line 563: References 7 and 31 do not mention center of gravity, only center of abundance. Please clarify.
Line 630: There is no information on variable correlation analysis (variable selection), but there is a figure in the supplementary.
Line 656: Replace bioecological with “ecological”.
Line 692: Bio-Oracle version?
Line 721: Extreme gradient boosting (XGBoost)-based machine learning method
Line 722: XGBoost algorithm, Line 726 XGBoost model, standardize method name
Line 730: Bottom O_2, or Dissolved Oxygen?
Fig 5. line 199: O2 tolerance or Dissolved Oxygen?

(Remarks on code availability)
The files and codes are complete and fully functional.

Version 1:

Reviewer comments:

Reviewer #1

(Remarks to the Author)
Firstly I would like to thank the authors for their detailed and thorough responses to all of the reviewer comments submitted at the last review.

All of my main concerns surrounding the manuscript and general approach have been addressed, often through additional and comprehensive statistical analyses.

I particularly like the approach taken to assess whether species having a distributional range larger than the survey areas impacted the ability to detect range shifts (lines 123 - 131). I was also glad to see that the removal of pelagic species did not cause large shifts in the results. One consideration is whether this needs to be in the main text, or the results for demersal species minus pelagics are presented, with the methods detailing that pelagics were removed. I think either way works, but that is an option to reduce some of the results text.

One further question is regarding the inclusion of some of the trawl data - on lines 392 - 394, you now show the spread of speed and duration for all hauls. Some of these seem both very slow (i.e. is the gear fishing properly < 1 kt?, depending on the gear, around 3 is ideal) and very fast (i.e. how did they manage to successfully deploy gear from a vessel moving 18 kts?). Equally, for short duration hauls - was this 5 minutes of bottom time? (I assume so) if not, this also may not have fished properly. It may be worth looking at the catch data for some of those outliers and removing them. You could even have some inclusion thresholds based on other trawl-focussed studies.

I have only a couple of minor edits, as follows:

Line 38: remove the second 'h' from shifting

Line 83: what are the SD/SE values for ave speed and age duration? I know more detailed values are provided in the methods, but some accounting of variability would be nice to have here as well.

Line 334: change 'limit' to 'limits'

(Remarks on code availability)
n/a

Reviewer #4

(Remarks to the Author)
Dear Authors,

I have completed my review of the manuscript. I am pleased to see that all of my previous observations have been fully addressed. Furthermore, the revisions made by the other reviewers have significantly enhanced the quality of this research. At this stage, I have no further comments.

(Remarks on code availability)
I have reviewed the supplementary material, finding the code and the results obtained in extenso.

Open Access This Peer Review File is licensed under a Creative Commons Attribution 4.0 International License, which permits use, sharing, adaptation, distribution and reproduction in any medium or format, as long as you give appropriate credit to the original author(s) and the source, provide a link to the Creative Commons license, and indicate if changes were made.
In cases where reviewers are anonymous, credit should be given to 'Anonymous Referee' and the source.
The images or other third party material in this Peer Review File are included in the article's Creative Commons license, unless indicated otherwise in a credit line to the material. If material is not included in the article's Creative Commons license and your intended use is not permitted by statutory regulation or exceeds the permitted use, you will need to obtain permission directly from the copyright holder.
To view a copy of this license, visit https://creativecommons.org/licenses/by/4.0/

Response to referees

Title : Pervasive deepening-driven range expansion of marine fish populations in a sea of change

We are grateful to the referees for their time and effort in evaluating our manuscript. Their comments greatly helped improve the clarity of the manuscript and prompted us to conduct additional analyses that further strengthen the presentation of our results and conclusions.

The revised manuscript is provided with tracked changes. All line numbers cited in the "Response to referees" document refer to the revised manuscript with revision marks hidden (these locations are also highlighted in yellow in the manuscript). Reviewers' comments are shown in **black**, and our responses are shown in **blue**.

Reviewer #1:

This paper presents a comprehensive analysis investigating depth and latitudinal shifts in fish populations across Europe. A majority of species display deepening ranges over time, with a 50-50 split of latitudinal shifts (species seem equally likely to move north or south). These shifts are then explored in the context of environmental change, species specific environmental niche and biological traits.

The analyses are advanced and appropriate to deal with complex and disparate data sources. Showing clear and sometimes unexpected trends in fish populations.
I have some general points as well as some specific comments which I will outline below.

We sincerely thank the reviewer for the time and effort dedicated to evaluating our study and for the constructive and insightful comments.

Throughout the manuscript surveys are referenced, which I know is the accepted convention. However, I think it may be clearer (especially in some parts of the methods e.g. line 545) to refer to survey time-series or programs which then lets you refer to individual surveys (i.e. a single instance within a survey time-series).

Yes, that is correct: "survey time-series" is more suitable than "survey". This has been corrected in the revised manuscript and supplementary materials.

Throughout the paper, mean and median shift speeds are presented which is very helpful, but I wonder whether % change may also have been useful to give a feel for how big these shifts are in comparison to the starting depth.

We thank the reviewer for this comment, which helps to assess the relative magnitude of depth shifts better. To address this point, we conducted two additional analyses.

The first analysis converted the absolute depth-shift velocity to a relative velocity, normalized by the starting depth, as suggested by the reviewer. We then generated a new supplementary table presenting the relative shift velocities for each species across survey time series (Supplementary Table 4). An additional figure (Supplementary Fig. 1) was produced to illustrate the

relative shift velocities across surveys. When considering only populations exhibiting significant increases in the mean depth over time, the median relative velocity was about 23% of the starting depth per decade across populations. By including non-significant increases in depth, this value was about 13% of the starting depth per decade.

The second analysis converted absolute velocities into percentages, but relative to the 'starting depth range' of the population (i.e., the difference between the maximum and minimum depth where the population was observed during the first 3 years of the survey time-series). Like the first analysis, this approach complements our assessment of the magnitude of individual population bathymetric shifts, but relative to the starting depth range. This is particularly meaningful to reflect on whether a change in centroid depth represents a large proportion of the initial population depth range (or conversely a rather marginal one). An additional figure (Supplementary Fig 2) was produced to illustrate these relative shift estimates, and a supplementary table (Supplementary Table 5) reports the percentage values by population (i.e. a species within a survey time series). When considering only populations exhibiting significant depth shifts, the median relative velocity was about 17% of the starting depth range per decade. By including non-significant increases in depth, this value was about 9%. All these new analyses are now referenced in the revised manuscript to better gauge the amplitude of depth shifts (see lines 93-97).

In addition, we have included figures (new Supplementary Fig. 3-5) that allow an assessment of absolute (new Supplementary Fig 3) and relative bathymetric shift velocities (new Supplementary Fig 4: velocity based on the 'starting depth', and new Supplementary Fig. 5: velocity based on the 'starting depth range'), separately for populations whose centers of gravity increased (deeper shifts) and those whose centers of gravity decreased (shallower shifts; see boxplots in new Supplementary Figs. 3-5). These new results are included in the revised manuscript through the supplementary figures and tables listed above.

The overall mean speed of depth shift is quite high (19 +/- 27 m.decade). Whilst I'm sure it is correct I wonder if it is masking regional differences. For example, a 19 m/decade shift would result in almost 80 m of deepening over the 40 years of data analysed. As we're dealing with largely demersal species (or at least the bottom gears limit the sampling depth to the maximum available) an 80 m deepening should presumably result in species being found on sea floor ~ 80 m deeper. However, this would not be possible in an environment like the North sea, with an average depth of 100 m. So far as I can see, regional (NeA & MedS) mean & median speeds are not presented, just the corresponding supplementary figures. But as a lot of the surveys in the NeA are on the shelf edge, this may also mask the pattern in the North Sea. This large environmental 'barrier' to deepening is not discussed, although there is good opportunity to do so alongside the discussion around limitations of latitudinal shifts due to the strait of Gibraltar (Lines 261 – 263).

We agree with the reviewer that regional differences require a more detailed description. Highlighting how depth constraints can limit deepening (in the same way that latitudinal shifts can be constrained by geographical constraints, i.e. coastlines - cf. discussion about the Mediterranean) is indeed crucial. We have included several additional analyses, and we acknowledged in the discussion section that some limitations still exist (lines 329-336).

The reviewer is correct that mean depth-shift velocities assessed across all survey time series may mask regional discrepancies. For instance, a narrow regional depth range may physically constrain population potential to shift deeper, unlike assessments completed using surveys that cover steeper depth gradients. To address this, we conducted several complementary analyses. First, we examined the relationship between the depth range of each survey time series and the species' bathymetric shift velocities. We found that the rate of species' bathymetric shifts is positively related to the survey depth range up to a threshold of around 100-120 m (see Figure below, now included in

Supplementary Information as Fig. 25). Beyond this threshold, however, the species' bathymetric-shift velocities appear to be independent of the survey depth range.

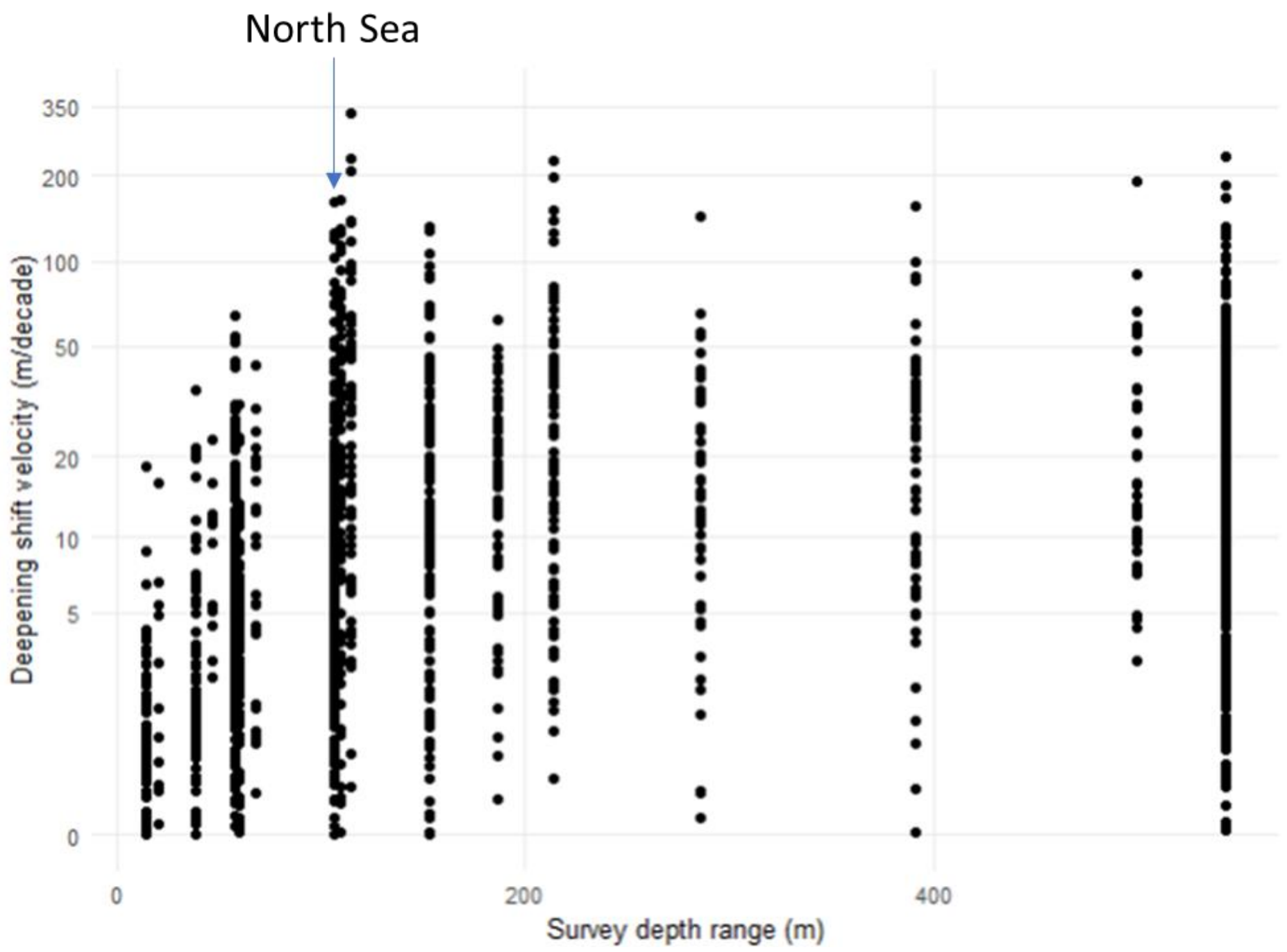

We can therefore confirm what the reviewer suspected: the overall mean value of depth shift of 19 ± 27 m per decade indeed masks the constrained depth changes in flattest surveys (i.e., < 100 m depth range), where our estimates of absolute depth shift velocities are lower potentially due to regional depth constraints. In the specific case of the North Sea survey (depth range: 23–133 m, see new Supplementary Table 8), species that significantly shifted deeper did so at a median rate of only 9 m per decade, representing less than 9% of the survey depth range. By considering all survey time-series, species shifted deeper at a median rate of 13 ± 12% of the survey depth range.

All these results therefore suggest that absolute depth shift velocities were only moderately constrained by survey depth ranges. However, we acknowledged that this constraint is still present in the flattest surveys conducted along the shelf edge of the Northeast Atlantic (i.e. BITS, BTS, BTS-VIII, DYFS, FR-CGFS, NIGFS and SNS; Supplementary Table 8). This is now acknowledged in the revised version of the manuscript (lines 333–336 and Supplementary Fig. 25).

There is no discussion or methods text devoted to the species included in the analyses. There is some explanation of exclusions based on number of observations (line 547), but it is unclear whether this refers to all analyses (i.e. the survey scale) or just the seasonal scale approach.

We thank the reviewer for their careful reading. The species selection described at l.547 ('*To avoid biases due to undersampled species, we only included in the calculations species present in at least 5 years in the abundance data set, resulting in a total of 419 species out of 562, which represents around 99.94% of total abundances*') was applied exclusively for the survey-scale analyses (i.e., 'survey time-series scale'). This point has been clarified in the revised version of the manuscript (see l. 419-421): '*At the survey time-series scale only, to minimize potential biases from undersampled species, we included only those present in the abundance dataset for at least five years. This selection resulted in a total of 419 species out of 562, representing approximately 99.94% of the total abundance*'.

There are likely to be varying gear selectivity issues around many of the species analysed. It is clear from some of the supplementary figures and explanations of the species traits that some species were pelagic for example (e.g. Clupea harengus, Scomber scombrus). These are likely to be poorly sampled as the bottom gear only fishes optimally when being towed on the sea bed. This means that observations and abundances of pelagic species are likely to be sporadic, but also more importantly, that depth information cannot be assigned to them as they have potentially been captured as gear is being hauled, rather than the underlying depth influencing their habitat/capture location. Similarly, depending on the gear type, mesh size etc. some small (or large) species may be very poorly/sporadically sampled.

We agree: this bias exists and represents a limitation of these survey-derived datasets that must be acknowledged. We particularly appreciate your point related to ineffective sampling of pelagic species with bottom trawl-fishing gear, so we have investigated the sensitivity of our results to the inclusion (or not) of these pelagic species in the analyses. This supplementary analysis (with/without pelagic taxa) reveals that the percentage of 'metapopulations' (hereafter referred to as 'populations', following another reviewer's suggestion) showing an increase in the depth of their center of gravity was relatively unchanged: from 60.6% to 60.4% when pelagic species were excluded from the analyses. Similarly, when considering only populations that exhibited a significant change in depth, the percentage of species showing an increase in the depth of their center of gravity changed from 75.4% to 76.6**%** after excluding pelagic species. The percentage of populations shifting northward remained virtually unchanged when pelagic species were excluded, decreasing only slightly from 50.4% to 49.3%. When considering only significant latitudinal shifts, this proportion also showed little variation, shifting from 53% to 51.9%. These additional results are now included in the revised version of the manuscript (see l. 120-123 in the *Results* section, as well as some of the following references).

The inclusion or exclusion of particular species in analyses, depending on their capture efficiency, poses a significant challenge, particularly because catchability can vary even among pelagic species (Heessen et al., 1997; Gislason et al., 2020). Similarly, we agree that assigning depth information to species most frequently caught during trawl deployment or retrieval (Trenkel et al., 2006) may compromise our ability to estimate potential bathymetric shifts accurately. We acknowledge that we did not previously include sufficient cautionary notes regarding these limitations and have now expanded our *Methods* (see l. 451-458) and *Discussion* sections (see l. 360-368) to address this issue more thoroughly.

As the reviewer points out, pelagic species, as well as very small or very large ones, are inevitably sampled less effectively than demersal, intermediate-sized species. Consequently, while the GOV trawl can be used to assess particular pelagic fish species, such data are inherently biased and should be interpreted with caution (Heessen et al., 1997). Catchability also depends on other factors, including behavioral traits such as the ability to avoid fishing gear (Arreguín-Sánchez, 1996; Cadrin et al., 2016), which may potentially change under different conditions or life stages. However, catchability can be estimated through experiments comparing catch per unit area swept to absolute estimates of local density derived from stock assessments, visual observations, depletion experiments, tagging studies, or modeling of herding and escapement (Gislason et al., 2020; Walsh, 1992; Somerton et al., 1999). Unfortunately, due to data limitations (e.g. lack of stock assessments) and the complexity of accounting for all parameters influencing catchability, such as weather conditions (ICES, 2025) or local habitat characteristics, direct estimates of catchability cannot be obtained for most species and survey areas (Gislason et al., 2020). Moreover, for the few species where such data exist, catchability shows substantial variability across regions (Gislason et al., 2020).

In parallel, the relatively high vertical opening of GOV trawls (5–6 m) nevertheless still enables the capture of some pelagic species, such as herring, which led to the adoption of the GOV trawl in 1977 to replace the former pelagic herring trawl (Heessen et al., 1997; ICES, 2012). Trawling for herring is preferably conducted during daylight hours, when the species moves closer to the seabed and thus becomes accessible to the GOV (Heessen et al., 1997). As a result, several bottom

trawl surveys, such as those conducted in the North Sea, have been reliably used in pelagic stock assessments (e.g., herring and sprat; ICES, 2007, 2022, 2025) and in community-scale analyses (e.g., Hiddink and ter Hofstede, 2008; Pecuchet et al., 2017).

All this said, we acknowledge that not all species are equally catchable. Consequently, we consider the bathymetric shifts observed for some species to be more robust and reliable than those observed for others. These points are now acknowledged in the revised version of the manuscript (see l. 360-368).

References:

Hiddink, J., and Ter Hofstede, R. (2008). Climate induced increases in species richness of marine fishes. Glob. Change Biol. 14, 453–460.

ICES, 2022. Herring Assessment Working Group for the Area South of 62° N (HAWG) report. https://ices-library.figshare.com/articles/report/Herring_Assessment_Working_Group_for_the_Area_South_of_62_N_HAWG_/19249010?file=38181423

ICES, 2007. Regional Abundance Surveys with input to the ICES Fisheries advice for the CFP, 21 pp.

Heessen HJL, Dalskov J, Cook RM (1997) The International Bottom Trawl Survey in the North Sea, the Skagerrak and Kattegat. ICES CM 1997/Y:31. International Council for the Exploration of the Sea, Copenhagen.

Trenkel V. M., Battaglia A. & Rochet M.-J. (2006). *Estimating end effects in trawl catches*. ICES Journal of Marine Science, 63(5), 956- 959. doi:10.1016/j.icesjms.2006.03.002

ICES. 2025. Herring Assessment Working Group for the Area South of 62° North (HAWG). ICES Scientific Reports. 7:20. xx pp. https://doi.org/10.17895/ices.pub.28389008

Arreguín-Sánchez, F. (1996). Catchability: a key parameter for fish stock assessment. *Reviews in Fish Biology and Fisheries*, 6, 221-242.

Cadrin, S. X., DeCelles, G. R., & Reid, D. (2016). Informing fishery assessment and management with field observations of selectivity and efficiency. *Fisheries Research*, 184, 9-17.

Walsh, S. (1992). Size-dependent selection at the footgear of a groundfish survey trawl. *North American Journal of Fisheries Management*, 12:3, 625-633.

Somerton, D., Ianelli, J., Walsh, S., Smith, S., Godø, O.R. and Ramm, D. (1999). Incorporating experimentally derived estimates of survey trawl efficiency into the stock assessment process: a discussion. *ICES Journal of Marine Science*, 56, 299-302.

ICES. 2012. Manual for the International Bottom Trawl Surveys. Series of ICES Survey Protocols. SISP 1-IBTS VIII. 68 pp.

Pecuchet, L. *et al.* From traits to life-history strategies: Deconstructing fish community composition across European seas: Pecuchet et al. *Glob. Ecol. Biogeogr.* 26, 812–822 (2017).

Gislason, H. *et al.* Species richness in North Atlantic fish: Process concealed by pattern. *Global Ecol Biogeogr* 29, 842–856 (2020).

Line-by-line comments:

Line 12: I know that the range here is representative of all metapopulations that display a shift (60%) and the % of all significant shifts that displayed a deepening trend (75%). These are two different concepts and it's unclear to the reader why a range is presented. I would just pick one or the other to present.

After several discussions about which of the two values should be included in the abstract, we had initially decided to report both. As suggested by the reviewer, we now include only one (i.e., 60%). In the main text, however, it is important to mention both values. This has now been corrected in the revised version of the manuscript.

Line 39: 562 fish species mentioned in text, but 613 present in your species trait dataset

We thank the reviewer for pointing out this mistake. It has now been corrected.

Before the first data-cleaning step (i.e., removing survey time series with fewer than 300 hauls), the total number of species was 612. Excluding these survey time series removed species occurring only in them, leaving 562 species in the final abundance dataset we used. We chose to retain the full trait dataset (612 species) in the data repository rather than the filtered dataset (562 species). This has now been clarified in the Methods section (l. 419-421), and '612' has replaced '562' wherever appropriate.

Line 74: change or to of

This is now corrected.

Line 101: 'associated with movements toward greater depths rather than toward the surface' This terminology seems misleading - this is an analysis of bottom/bean trawl survey data, demersal species changing depth distribution would likely reflect a geographic shift along with depth, but pelagic species could shift within the water column (although I don't think thew would be adequately sampled to draw conclusions).

We agree with the reviewer: the wording here can be misleading. We changed the sentence as follows: "*More specifically, the spatial expansion of populations was primarily associated with geographic shifts toward deeper rather than shallower areas, whereas northward and southward expansions were proportionally similar*". This should now prevent any reference to the idea of vertical movements. See l. 167–170.

Line 103-104 & lines 232-234: You mention here that the majority of metapopulations (69%) were characterized by an increase in abundance – was this also true for commercially exploited species? From your other analyses I think it should be easy to isolate these and may add nicely to your discussion around fishing pressure if it shows abundance of commercial species has displayed no increase. Or indeed if they have increased, potentially as a result of common fisheries policy.

Thank you for this valuable suggestion. We recalculated the percentages by excluding commercial species and, conversely, by considering only commercial species. The results remained consistent, changing only from 69% to 68% when commercial species were excluded and from 69% to 72% when non-commercial species were excluded. We have now included this information (lines 170-176).

In addition to this binary analysis (i.e. 'commercial' vs. 'non-commercial'), we examined all six commercial status categories in FishBase (see the new Supplementary Table 10 and the figure below). For the 'highly commercial', 'commercial', and 'minor commercial' categories, we found that most populations showed increasing abundance (58%, 71%, and 73%, respectively), with significantly positive median rates of change in abundance. In addition to the binary analysis, these results suggest that the progressive reduction in fishing effort in recent decades may have (potentially) contributed to a general increase in species abundance in EU waters. Interestingly, species 'of no

interest' significantly increased too, also suggesting a positive effect of the reduction in fishing effort. We have now included this information (now Supplementary Fig. 13) in the *Discussion* section (lines 301-311).

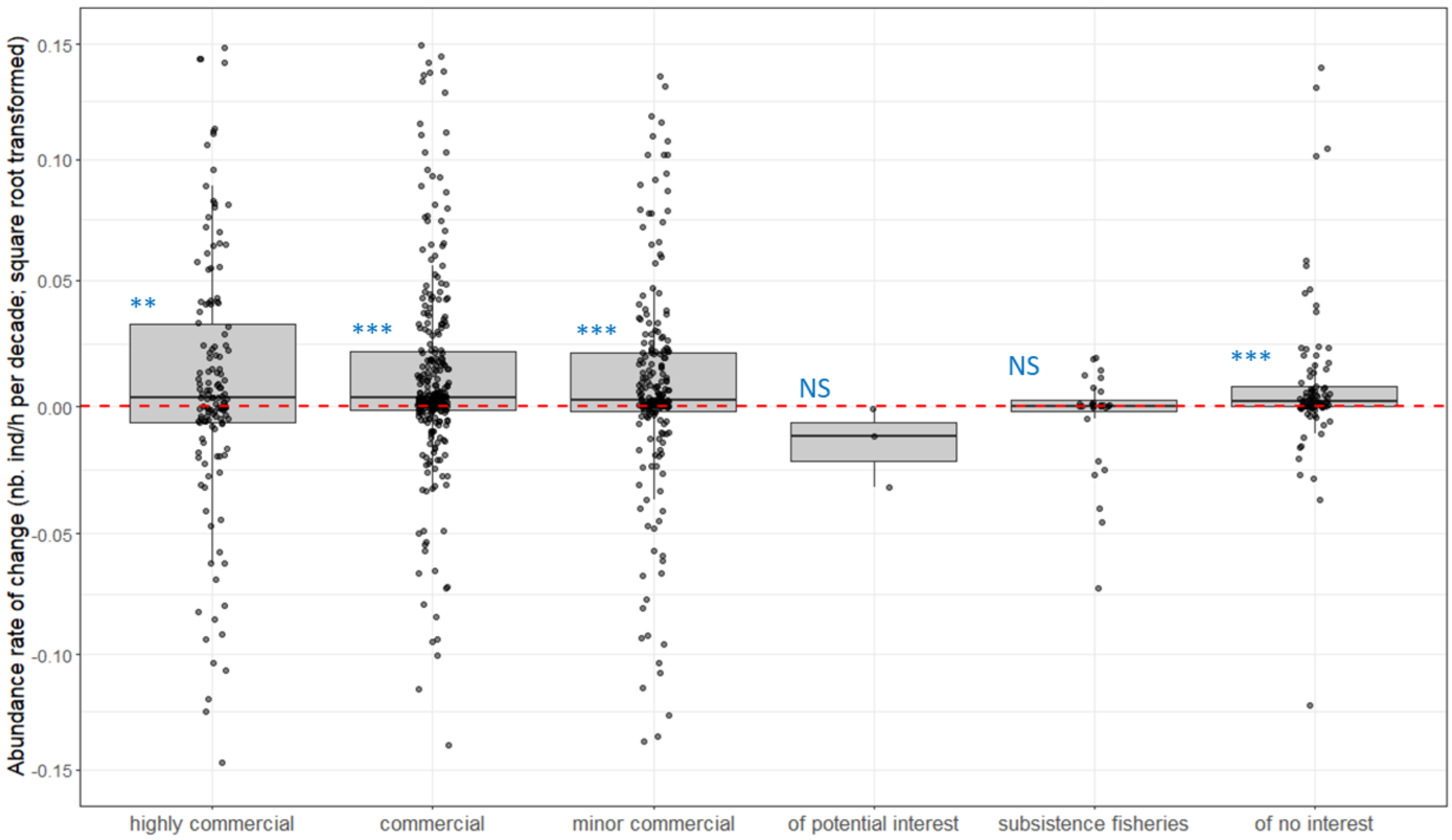

Lines 234 – 238: These two sentences seem to say opposing things. Firstly that the higher fishing pressure in shallower areas may have led to the shift in centroids (presumably deeper as this is the general trend reported). But the following sentence then says that the common fisheries policy has reduced fish pressure which has also led to deepening. Surely a reduction in the pressure outlined in the first sentence would result in shallowing as these populations re-colonise historical habitat as pressure is reduced (not accounting for environmental conditions).

The reviewer is correct, and this prompted us to conduct additional analyses. The confusion stems from the fact that our second sentence was not clearly written or appropriately placed within the paragraph, which may mislead readers. The sentence n°1 (i.e., "*In parallel, the fact that fishing efforts are generally stronger in shallower waters, particularly from artisanal and recreational fisheries, may have contributed to the shift in population centroids through the uneven capture of individuals*") indicates that the bathymetric distribution of fishing effort may have contributed to species' centroids shifts toward deeper areas, which is correct. The sentence n°2 (i.e., "*Building on all these findings, we emphasize that the relocation of fish species, particularly their shift towards deeper waters, was also likely influenced by the gradual reduction in fishing pressure associated with the Common Fisheries Policy, one of the most comprehensive frameworks for sustainably managing European fishing fleets and conserving fish stocks*") is misleading as it refers more generally to species relocation, suggesting that reduced fishing pressure may have contributed, at least in part, to spatial expansion and overall increases in abundance. In that sentence, we mistakenly added the phrase '*particularly their shift toward deeper waters*,' which may lead readers to focus on deepening specifically. We have now removed this emphasis, rephrased sentence 2, and repositioned it within the paragraph.

That said, the reviewer is correct in noting that a decrease in fishing effort could lead to shallower rather than deeper distributions. This is indeed a plausible outcome. If absolute fishing effort were lower in shallow waters than in deeper waters, one could reasonably expect species'

distribution centroids to shift upward. However, in the regions we studied, although overall fishing pressure has generally declined, absolute fishing effort has consistently remained higher in shallow waters than in deeper areas (new Supplementary Fig. 23). Under these conditions, it is reasonable to hypothesize that fishing may have contributed, at least in part, to the observed deepening process.

The reviewer's comment, therefore, prompted us to examine how fishing pressure has evolved along the bathymetric gradient (Fig. 24). We conducted this analysis for each survey time series and found that the rate of decrease in fishing effort was greater in shallow waters in 29 out of 33 survey time series. This supports the reviewer's intuition. However, this differential rate of decline was insufficient to reverse the bathymetric gradient in absolute fishing effort. Consequently, although fishing effort has decreased more rapidly in shallow areas, the absolute fishing effort remained higher there than in deeper waters, and thus may have contributed more to deepening than shallowing shifts.

We thank the reviewer for pointing out this confusion between the two sentences. This is now clarified with reference to the supplementary results (l. 286-311).

Line 250 (and discussion generally): lots of the discussion text is in reference to improved fishery management, but surely this would only apply to those species impacted by fisheries (either as target or bycatch species)? From the supplementary figures 13 & 14 it looks like more than half the species are not correlated with fishing effort.

Our message was that reduced fishing pressure may have contributed positively to the generalized increase in abundance and spatial expansion of all populations (including commercial and non-commercial species). However, we agree with the reviewer that the positive effects should mostly apply to species impacted by fisheries (especially target species), but in that sentence, we also aimed to acknowledge that management measures may potentially have generated positive effects also on non-commercial species, including those that are not necessarily correlated with fishing effort, such as bycatch species and species not caught by bottom trawls. Indeed, improved fisheries management should lead to reduced fishing effort and, consequently, less physical disturbance of benthic habitats. In turn, these reduced habitat impacts should lower incidental mortality, including cryptic mortality of species not caught by bottom trawls.

The initial sentence "*Fisheries are largely documented as a driver of stock abundance decline, or range contraction, but our findings suggest that observed abundance increase could be due to improved fishery management practices in the EU over the past decades*" is now removed. We also removed the sentence '*Given the widespread patterns across most fish species, we can hypothesize that most range shifts relate to observed increases in both abundance and range extent of local metapopulations, which possibly result from successful fisheries management in EU waters*' to delete the repetition. Finally, the fact that fisheries management policies may also have potentially contributed to the observed increase of some non-commercial species (e.g. 'of no interest' species in Supplementary Fig. 13) is now included in the revised version, although these effects appear to be very limited.

Line 257: 'effective fishery management is highly likely to restore exploited stocks, promoting their ability to fill these niches.' As above, if fishing activity has resulted in deepening for some species, this would result in shallowing?

In relation to a previous reviewer's comment, we have examined how fishing pressure has evolved over time along the bathymetric gradient. We observed that the rate of decrease in fishing effort was greater in shallow waters in 29 out of 33 survey time series. However, this differential rate of decline was not sufficient to reverse the bathymetric gradient of absolute fishing effort (new Supplementary Fig. 23). Hence, although fishing effort has decreased more rapidly in shallow areas, the absolute fishing effort remained higher there than in deeper waters, and thus may have contributed more to

deepening than shallowing shifts.

Line 261-263: see comment above about depth constraints across which is likely as big (or bogger) of a factor as depth shifts seem to be more prevalent.

Following a comment from a previous reviewer, we have now placed greater emphasis on this depth constraint in the Discussion section (see l.329-336).

Lines 270 – 274: I completely agree, something like a food-web modelling approach would compliment this study very well.

Thank you for this suggestion; this is now included in the sentence (l. 344-346).

Line 521: what are the max, min, mean values for speed and time?

In addition to the minimum, mean, and maximum, we have now also added the 1st and 9th deciles, as well as the median, to better characterize the distributions of vessel speed data and trawling durations. The initial sentence is now reworded, both in the Methods section and Fig.1 caption (see l. 392-394 and lines 82-84).

Line 545: individual surveys or survey time series with fewer than 300 hauls over multiple years in a single quarter?

This is for the entire survey time series. We have now clarified this point in the Methods section (l. 419-421).

Lines 554 – 555: You removed species not present in < 50 hauls, was this done for survey-level analyses as well?

This filtering step was applied only at the European scale. Applying it at the survey time-series scale would have removed many species for which we can reliably assess bathymetric and latitudinal shifts. Conversely, not applying this filter at the European scale would have resulted in poorly estimated shifts, particularly for spatially restricted species. This is now clarified in the Methods section (l. 430-431).

Supplementary material:
Figure 6: closing square brackets are facing the wrong way on the y-axis.

The square brackets are intentionally used this way to avoid assigning a value (i.e. value used as one of the two limits of an interval) to two intervals. For example, the value 0.8 belongs only to the interval [-1.0; -0.8] and not to the next interval]-0.8; -0.6].

Table 3 & 4: I can't imagine that some of these species are particularly common in standard surveys. Is it possible that some of these large shifts are driven by low sampling and that the capture of many of these species is dependent on gear selectivity which is likely not optimized for rare/small/oddly shaped species. Pelagic species are likely sporadic in catches (e.g. engraulids, myctophids) if bottom trawl time-series were used.

Some species also have both deepening and shallowing trends within the same geographical area (e.g. Coelorinchus caelorhincus). Scomber scombrus (pelagic - likely not sampled well with these approaches.). But I can't see this discussed anywhere. It possibly suggests that sampling biases over-ride any environmental/biological signals.
Grouping the NeA into one category likely smooths over lots of differences between shelf and shelf-edge habitats. Do common species between these habitats show the same trends?

In line with our previous response regarding catchability, we fully acknowledge that we did not previously provide sufficient cautionary notes concerning these limitations and have now expanded our discussion of this issue, including additional results (l. 102-108), in both the Methods (see l. 451-458) and Discussion sections (see l. 360-368).

The reviewer's point about species exhibiting opposite trends within the same region (e.g., Coelorinchus caelorhincus) prompted us to revise the former Supplementary Tables 3 and 4. In the previous version, we chose to display only the 50 species with the most rapid deepening or shallowing shifts (the same for latitudinal shifts), regardless of the statistical significance of these trends (for example, one of the two opposite trends for *Coelorinchus caelorhincus* was not significant). We have therefore revised the former Supplementary Tables 3 and 4 to include only species exhibiting significant trends and the highest shift velocities, which we believe is more informative than simply listing the 50 fastest shifts. Although these analyses cannot fully resolve differences in catchability across species, we believe it is still essential to present these trends for poorly sampled species. However, these trends should be interpreted with greater caution than those of demersal species.

Reviewer #2:

Summary
This paper presents a large-scale analysis of fish distribution shifts across European seas using decades of standardized trawl survey data. The study finds that deepening is the dominant response, while latitudinal shifts appear more balanced. The work is interesting, well-written, and relevant for both ecology and fisheries management.

Thank you for this positive comment and, more broadly, for your valuable suggestions, which have greatly enhanced the clarity and overall quality of our manuscript.

However, the novelty is somewhat limited as previous studies have already shown many of the patterns found here. For example: Chaikin et al. 2021 (https://doi.org/10.1111/geb.13414) demonstrated widespread deepening of fish populations in the Mediterranean. Gordo-Vilaseca et al. 2023 (https://peerj.com/articles/15801/) demonstrated some northward shifts in the North, Barents, and Norwegian seas. Supplementary Tables 1 and 2 do cover such results, so it's not as though they're ignored here. What's perhaps most novel in this paper is the analysis tying shifts to explanatory variables including environmental changes and fishing. Still, I'm not sure there's enough novelty here to justify publication in Nature Communications rather than a top quality fisheries journal - for example: Fish and Fisheries or ICES JMS.

Thank you for the reference to Gordo-Vilaseca et al. (2023), which we had previously missed. It is now included in the supplementary table listing all studies investigating latitudinal and bathymetric shifts.

We agree that a major novelty of our work lies in the in-depth examination of the mechanisms driving latitudinal and bathymetric shifts, achieved by integrating a wide range of potential drivers, including environmental changes, species traits, safety-margin traits, and fishing pressure. We have revised the manuscript to place greater emphasis on this aspect and on the associated limitations, particularly in the Discussion section (l. 283–311; l. 325–341). We also rephrased a key sentence in the abstract as follows: *"Here, we quantify how populations relocations are driven by the interplay between environmental factors and species traits as species track their optimal ecological niches".* In addition, we slightly reduced the emphasis on the deepening–northward mismatch by removing unnecessary redundancies in the Discussion.

At the same time, an essential contribution of our study lies in the pronounced mismatch we reveal between deepening and northward shifts, based on a large dataset (>105,943 hauls) collected over four decades and encompassing a substantial number of fish species (>400). Compared with previous studies, ours covers a broader spatial scale, includes more species, and relies on a larger number of sampling stations, providing important new insights and a more comprehensive and integrative view of past dynamics at the European scale. Moreover, most previous studies have focused on only one of these two types of shifts and typically at a local scale. Consequently, documenting such a clear mismatch between bathymetric and latitudinal shifts using such an extensive dataset represents another key strength of our study.

In all cases, we agree with the reviewer and have revised the manuscript to place greater emphasis on the fact that our study primarily contributes to understanding the drivers underlying these shifts.

Main suggestions:
1. I was wondering if the deepening pattern might partly come from benthic fish moving farther offshore, since depth and distance from the coast are usually linked. It could also just reflect relative declines in nearshore abundance (for example, from nearshore fishing pressure) rather than actual movement. In that case, the center of gravity would still shift deeper even if fish didn't really move. It

might be worth the authors mentioning this possibility and suggesting some methods to test it as sensitivity analysis.

We thank the reviewer for this important point. A few years ago, we developed a numerical approach to decompose the temporal dynamics of a weighted-mean type index, using the concrete example of the tropicalization process of communities in response to environmental change (McLean et al., 2021 and then re-used in Chust et al., 2024). The goal was to determine whether the increase in the community's mean thermal preference index was primarily driven by a rise in the abundance of warm-affinity species or by a decline in the abundance of cold-affinity species. It would therefore be relevant to apply a similar methodology here to determine whether the observed bathymetric shift in population centroids primarily results from an increase in abundance at greater depths or from a decline in shallower areas (possibly linked to fishing pressure in coastal waters). In relation to the reviewer's comment, as bathymetry and distance to coast are linked, it would also be relevant to examine whether the centroids, when considering both latitude and longitude, tend to move offshore, and to assess whether this offshore shift is mainly driven by an increase in offshore abundances, by a decrease near the coast, or both. These analytical suggestions have now been incorporated into the discussion section (see l. 374-379).

Similarly, to distinguish spatial changes in abundance from actual movement, one could compare estimated shifts derived from abundance data with those based on presence-absence data. This point has now also been incorporated into the discussion section (see l. 316-318).

References:

McLean, M. *et al.* Disentangling tropicalization and deborealization in marine ecosystems under climate change. *Curr. Biol.* **31**, 4817-4823.e5 (2021).

Chust, G. *et al.* Cross-basin and cross-taxa patterns of marine community tropicalization and deborealization in warming European seas. *Nat. Commun.* **15**, 2126 (2024).

2. One potentially important omission is the role of geographic barriers in constraining latitudinal shifts. Even though the analysis separates the Northeast Atlantic and the Mediterranean, there are still obvious land barriers within those regions. These barriers likely restrict species from showing clear poleward movements, even under strong warming. This contrasts with studies from more continuous coastlines (like the U.S. east coast but see Gulf of Mexico patterns in Pinsky et al. 2013), where poleward shifts are more detectable because alongshore connectivity is higher.

We agree with the reviewer that geographic barriers are likely to restrict species movements along the latitudinal axis. Similarly, such limitations may also apply to bathymetric barriers, for example, the physical constraint preventing a species from moving deeper than the maximum depth of its habitat. Emphasizing the constraints imposed by both bathymetric and latitudinal barriers on species distributional shifts is therefore essential. These points are now addressed in the Discussion section with more emphasis on land barriers (lines 325-341):

"Species range shifts may result from an intricate interplay of mechanisms, increasing the risk of drawing erroneous conclusions. In this context, the three-dimensional topology of the seafloor and land mass pattern may constrain species responses locally. For example, species in the northern MedS could theoretically shift poleward but are unable to move directly northward due to landmass and the narrow strait of Gibraltar to the West, which blocks access to the Atlantic. Analogous to the limitations imposed on latitudinal shifts by land barriers (e.g., southern coasts of England), inherent bathymetric constraints likely limited our ability to fully assess bathymetric shifts. Specifically, species that tend to deepen may have been physically restricted by the maximum depth of the study area (the 'seafloor barrier'). While our results show relatively low bathymetric shift velocities (13 ± 12% of the

survey depth range per decade), we must acknowledge that this seafloor barrier likely limit the actual deepening potential of certain species, particularly within surveys exhibiting low depth range (Supplementary Fig. 25). Likewise, incorporating the distribution of land barriers is essential for accurately assessing and interpreting changes in species distributions. Hence, accounting for physical constraints that create environmental mosaics (e.g., directional dependence of conditions) therefore appears to be a key step to properly understand the drivers of spatial movement patterns. For example, bed shear stress, light availability, seafloor morphology, proximity to upwelling areas, and sediment type are among the numerous factors that may influence species distribution shifts".

3. The whole idea of deepening and poleward shifts is usually based on the assumption that temperature is the key driver — with global warming pushing species deeper or toward the poles to stay within their preferred thermal range. But in this study, the models suggest that bottom temperature has almost no effect on deepening. That means the fundamental hypothesis driving "deepening" and "poleward" shifts seem doesn't really hold here, which I think the authors should acknowledge more clearly.

While we previously mentioned that species responses appeared to result from a "broad range of environmental changes" (second paragraph of the Discussion), we agree that the role of temperature, which appears to be relatively minor (from XGBOOST results and the 'niche tracking approach'), was not sufficiently emphasized. We concur that this is a critical point to highlight. The sentence has now been revised to explicitly reflect the comparatively weak influence of temperature (see l. 283-286): "*Our findings highlight that, contrary to common assumptions, sea temperature was not the primary driver of distribution shifts. Instead, these shifts were largely determined by species traits and environmental safety margins in response to a broad range of environmental changes, while fishing pressure emerged as a minor contributor*".

4. One limitation is the survey coverage. The standardized surveys were designed around the species' original distributions, so if species have shifted outside that coverage, the surveys may not capture them. This is a known issue for many highly mobile or high-seas species. I'm not sure how relevant it is here, but it might be worth mentioning.

While the supplementary analyses are quite reassuring (Suppl. Fig. 5 of the initial version, Fig. 11 of the revised version), we have now highlighted this critical point in the Discussion section (see l. 368-374), in addition to the Methods (l. 459-464) and Results (123-131).

Methods section: *'Because the latitudinal range of each population within a given survey time-series may represent only a fraction of its full global distribution (Supplementary Fig. 10), our ability to detect latitudinal shifts may be limited. To assess this limitation, we conducted a complementary analysis to determine whether the balance between northward and southward shifts remained consistent across populations pools stratified by their relative latitudinal range—that is, the proportion of a species' global latitudinal distribution encompassed by the population's range within a given survey time-series.'*

Results section : 'Because the latitudinal range of each population may represent only a fraction of its overall global distribution (median = 33%; Supplementary Fig. 10), our ability to detect latitudinal shifts may be limited. To assess this limitation, we conducted a complementary analysis to determine whether the observed balance between northward and southward shifts remained consistent across species pools stratified by their relative latitudinal range—that is, the proportion of a species' global latitudinal distribution encompassed by the population's range within a given survey time-series. This analysis confirmed that the balanced pattern of northward and southward shifts persisted even when excluding populations whose survey-scale latitudinal range was small relative to their global-scale range (Supplementary Fig. 11).

Discussion section: "*We should also note that, although the survey time-series scale offers numerous advantages, the limited spatial coverage of these surveys may not have fully captured latitudinal or bathymetric shifts for some species, particularly for highly mobile species".*

Minor suggestions:
"meta-population" doesn't seem like the right term here as it already has a specific meaning (see Hanski and Gilpin) that is not what's meant here. How about "stock" or simply "population"?

We have now replaced 'metapopulation' by 'population' throughout.

Line 25–27: the logic feels inconsistent — if fisheries are "overlooked," how can most studies also be focused on commercially important species?

The reviewer is correct: the wording was inconsistent. We intended to say that only a few of the exploited species have been examined in the literature. We have now rephrased the sentence to clarify this point: "*Counterintuitively, human-induced pressures, particularly those related to fisheries, are often overlooked, even in historically exploited regions like the Northeast Atlantic (NeA) and the Mediterranean Sea (MedS). In these areas, research has primarily concentrated on the effects of rapid ocean warming, while the few studies addressing commercial species have focused only on a narrow range of taxa*" (see l. 50-54).

Supplementary Tables 1 and 2: not sure if the shading is supposed to be red or yellow. Also, what about the Gordo-Vilaseca et al. 2023 paper mentioned above?

The color has been corrected to yellow, and Gordo-Vilaseca et al. (2023) has now been included in the table.

Supplementary Figure 12: some of the y-axis labels fall outside the panel boundary.

Thank you for your careful reading. This has now been corrected.

Reviewer #3:

I co-reviewed this manuscript with one of the reviewers who provided the listed reports. This is part of the Nature Communications initiative to facilitate training in peer review and to provide appropriate recognition for Early Career Researchers who co-review manuscripts.

Thank you for taking the time to review our work. We are grateful for your suggestions, which have helped improve the clarity of our manuscript.

Reviewer #4 :

I have completed the review of the paper entitled "Pervasive Deepening-Driven Expansion of Fish Metapopulations in a Sea of Change." This study looks at how fish populations are moving in European seas over the last four decades, focusing on shifts in depth and latitude.

We sincerely thank the reviewer for the time and effort dedicated to evaluating our study and for the constructive and insightful comments.

The main strength of this study is its integrative approach using a large amount of data (>105,943 trawl shifts) over four decades, covering 562 fish species. It integrates analyses of movements in depth and latitude, along with assessments of species traits, environmental conditions, and fishing pressure. This allows for an integrated and reliable understanding of complex patterns of redistribution of marine species in response to climate change and human activities, providing findings relevant to fisheries conservation and management.

Thank you for this positive remark. We compiled the most comprehensive dataset available for this region to minimize the effects of heterogeneity in sampling schemes (e.g. different times of the year, varying temporal windows, etc.) and in the spatial distribution of sampling effort. In addition to this strength related to data quantity, we also applied a series of methodological approaches to reduce these sources of variability further, although they cannot be completely eliminated.

The study presents potential biases that could affect the interpretation of its results. One of these is the limitation in detecting latitudinal shifts, since the latitudinal range of metapopulations at the sampling scales may represent only a fraction of the species' global range, which could bias the perception of the direction and magnitude of poleward or equatorward shifts.

We agree that this limitation in latitudinal range represents the main constraint in detecting potential latitudinal shifts. We did not express sufficient caution about this critical point in the Discussion, despite having applied a series of methodological choices to maximize our ability to detect genuine northward or southward shifts.

Initially, to prevent this type of bias, we chose to analyse all survey data (i.e. what we referred to as the 'European scale' in the manuscript). However, we later realized that considering only the European scale also has its advantages and limitations (please see below the original slide we prepared on this topic). Consequently, we decided to perform analyses at two scales to provide

the most integrative approach.

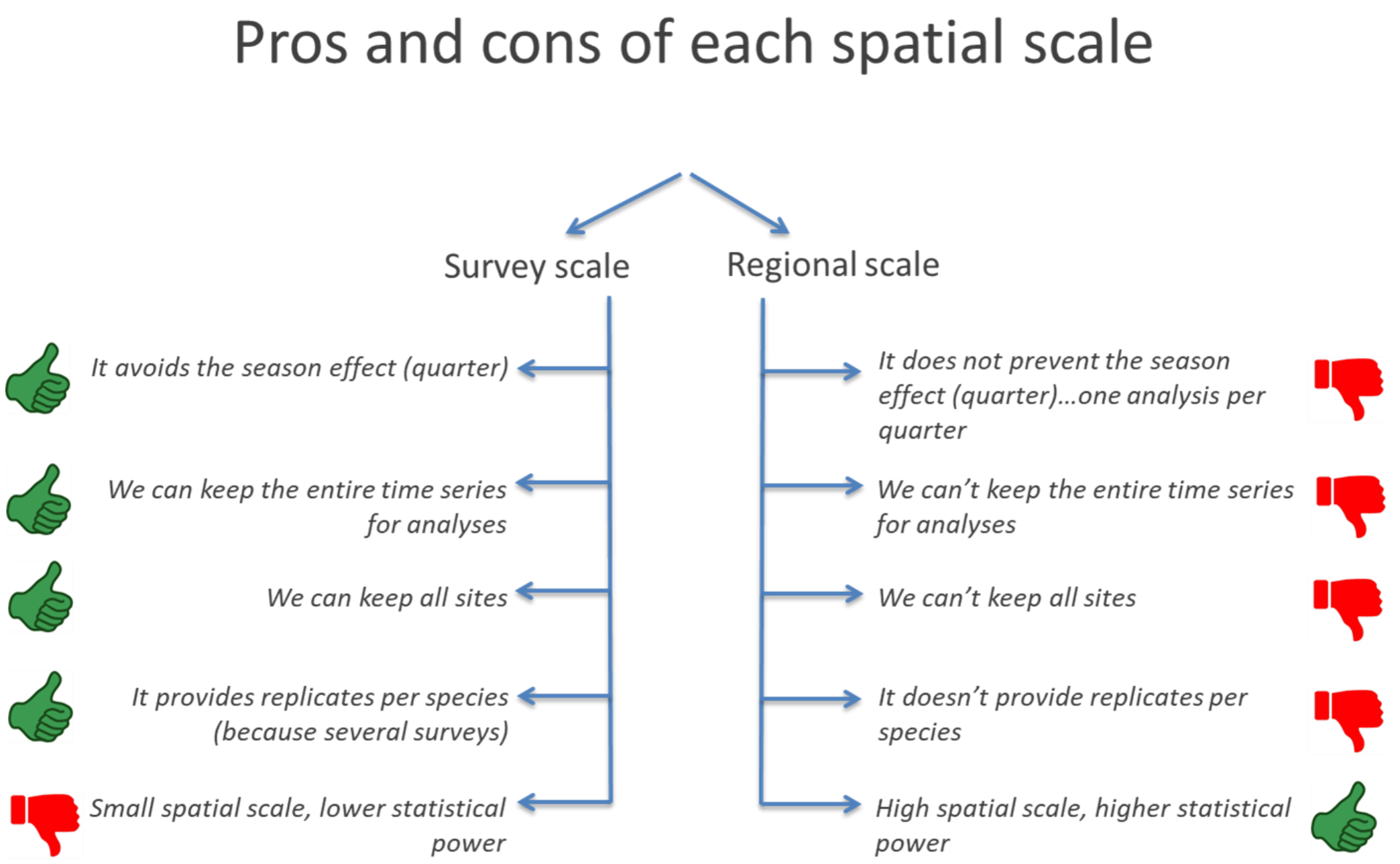

In the *Results* section (l. 123-131), we have now included a new supplementary result illustrating the distribution of percentages in global latitudinal ranges represented by the latitudinal ranges of populations at the survey scale (median=33% of the global scale range; new Supplementary Fig. 10), in addition to the new Supplementary Figure 11 in the following sentences: "*Because the latitudinal range of each population within a given survey time-series may represent only a fraction of its full global distribution (Supplementary Fig. 10), our ability to detect latitudinal shifts could be limited. To assess this potential limitation, we conducted a complementary analysis to determine whether the observed balance between northward and southward shifts remained consistent across species pools stratified by their relative latitudinal range—that is, the proportion of a species' global latitudinal distribution encompassed by the population's range within a given survey time-series. This analysis confirmed that the balanced pattern of northward and southward shifts persisted even when excluding populations whose survey-scale latitudinal range was small relative to their global-scale range (see Supplementary Fig. 11)*".

Although the dataset is substantial (33 time series; >105,943 hauls) and the supplementary analyses are reassuring, we have also highlighted this important point in the Discussion section (see l. 368-374) and in the Methods section (l. 459-464).

Methods section: "*Conducting analyses at the survey time-series scale offers several advantages: (1) because survey time-series are season-specific, they do not capture seasonal variability in abundances and distributions; (2) consistency in gear and survey design across the entire time series and all sampling stations means that all data can be retained for analyses; (3) estimates of species range shift patterns are replicated thanks to the multiplicity of survey time-series; and (4) each species in each survey time-series can be considered as an independent data point, as they are often demographically independent across different survey time-series. In contrast, the spatial extent of the survey time-series is smaller than that of the European scale. Consequently,if a species with a very localized range, particularly a highly mobile one, has shifted beyond the survey area, its latitudinal or bathymetric shift may not be fully captured by the survey-scale analysis. We therefore performed analyses at both scales for comparison.*"

Discussion section: "*We should also note that, although the survey time-series scale offers numerous advantages, the limited spatial coverage of these surveys may not have fully captured latitudinal or bathymetric shifts for some species, particularly for highly mobile species (median=33% of the global scale range; Supplementary Fig. 10)*".

Furthermore, although the analysis covers a large number of species and long-term data, heterogeneity in sampling methods, capture efforts, or local environmental conditions could introduce biases or variability that cannot always be controlled for in the models.

Yes, this is true. This leads to the conclusion that monitoring programs would benefit greatly from harmonizing their protocols, a process that is indeed underway, but unfortunately progressing slowly (now included in the *Discussion* section, l. 371-374). In this study, although controlling for such variability remains challenging, we sought to minimize the undesired effects arising from heterogeneity in sampling schemes and efforts by applying a series of filters designed to reduce these biases (e.g. removing rare species, excluding latitudes not sampled throughout the entire time series, omitting surveys with few years or few hauls, applying square-root transformations of abundances, and separating surveys by season). Furthermore, to avoid biased estimates of gravity centers caused by uneven sampling effort, we randomly resampled the data 100 times to ensure a consistent number of hauls along both the latitudinal and bathymetric axes for each species in each survey time-series.

The potential influence of the presence of species with very localized ranges versus widely distributed species can also bias the results. Supplementary material representing this information is needed, for example, descriptive statistics of distribution ranges.

In relation to a previous reviewer's comment, we have now highlighted this point in the revised version of the manuscript (see l. 123-131). In addition to the sensitivity analysis based on relative latitudinal ranges (i.e. new Supplementary Figure 11), we have now included an additional supplementary figure illustrating the distribution of global latitudinal ranges in percentages represented by the latitudinal ranges at the survey scale (median = 33% of the global scale range; new Supplementary Fig. 10).

Scientific findings

This study highlights that deepening is the most common response in fish populations in the Atlantic and the Mediterranean Sea. These finding challenges traditional expectations and previous research that prioritized latitudinal shifts.

The extensive database provides a comprehensive and detailed view of shift trends at the metapopulation level, something that few studies address on such a scale. It presents a multidimensional and multifactorial perspective, offering a realistic view of the phenomenon, which provides a more complete perspective on the effects of climate change.

Thank you for this positive comment.

However, caution is needed regarding the role of fishing and management. The authors state that the reduction in fishing pressure due to management policies has facilitated the spatial expansion of species, introducing a management dimension that is not always considered in other studies focused solely on ecological responses to climate change. However, it should be noted that more evidence is needed from other regions, as this could be misinterpreted and used as an argument for missuse or overexploitation of resources. I urge the authors to develop a sentence with the limitations and cautions of this statement.

We fully agree and thank the reviewer for highlighting the importance of exercising caution when discussing the effects of fishing and management in this study. We have now reviewed all statements relating to fishing, in particular the gradual reduction in fishing effort under the Common Fisheries Policy. We now adopt a more precautionary tone throughout the manuscript, particularly in the *Discussion* section (l. 306-309). In addition, as suggested by the reviewer, we have added a sentence to eliminate any remaining ambiguity: "*These relocations could be consistent with an overall positive trend in population dynamics that may in part be influenced by the progressive reduction in fishing effort under the Common Fisheries Policy, one of the main frameworks for sustainably managing European fisheries. This was supported by the significant temporal trends in abundance among populations, most of those corresponding to exploited species exhibited increasing trends (highly commercial: 58%; commercial: 71%; minor commercial: 73%), with significant positive median values in abundance rate of change (Supplementary Fig. 13). However, these patterns do not allow us to attribute causality. A substantial percentage of populations (38% to 51%; Supplementary Fig. 16) responded to environmental changes in ways that did not align with their expected ecological niches. This pattern is consistent with, but does not demonstrate, the potential influences of fisheries management. Further work, including targeted analyses and broader spatial replications, is required before drawing firm conclusions about the role of fishing reduction in shaping these trends*". (l. 297-311).

We also deleted the word 'positive' before 'effect of fishery…' in that sentence: '*While negative impacts of fishing on exploited stocks are well-documented (Edgar et al., 2024), effects of fisheries management on population abundance and subsequent range expansion deserve dedicated future work, as this poorly characterised mechanism is likely to mitigate or interact with climate-driven redistribution of marine life*' (see l. 318-321).

We also reworded the end of *Abstract*: "*Here, we quantify how populations relocations are driven by the interplay between environmental factors and species traits as species track their optimal ecological niches. We also highlight that the recent reduction in fishing pressure has likely facilitated spatial expansions and increases in populations abundance*". (see l. 38-41).

While current measures have contributed to reducing pressure on fishery resources, the distribution of species toward deeper areas is a significant finding.

We agree. For information, in response to a comment from another reviewer, we conducted an additional analysis to examine how fishing effort has changed over time along the bathymetric gradient (new Supplementary Fig. 24). We performed this analysis for each survey time series and found that the rate of decline in fishing effort was greater in shallow waters in 29 out of the 33 series. At first glance, such a decline can result in shallower rather than deeper distributions. However, this differential rate of decrease was insufficient to invert the bathymetric gradient in absolute fishing effort. In other words, although fishing effort has declined more rapidly in shallow areas, absolute fishing effort has remained higher there than in deeper waters, and thus may have contributed more to deepening than to shallowing shifts. This additional result is now added in the *Discussion* section (l. 288-290).

Methods

Overall, the statistical methods used in this study are appropriate for addressing the research questions. The use of machine learning models such as XGBoost is appropriate given their ability to handle complex relationships and high-dimensional data. However, the use of these models requires addressing overfitting, an issue that should be described in the methodology.

Thank you very much for this important reminder. We have now included more information in the Methods section. Below is the more detailed reasoning we followed:

In this study, we apply an integrative approach by incorporating all predictors (i.e. all species traits, environmental safety margins, environmental trends, and fishing). In addition, our primary aim was to take a qualitative perspective by estimating the direction of each predictor's effect on the probability that a species would shift to deeper waters or move northward. To provide the most accurate assessment of the influence of all our predictors, we required a method capable of addressing several challenges, particularly handling missing data and avoiding biased coefficient estimates due to over-parameterization. However, as the reviewer pointed out, machine-learning models are generally more prone to overfitting. Therefore, we have now included the overfit estimation in the revised Methods section (see l. 674-676). To quantify overfitting, the AUC was computed separately for the training data and the test data. The relative difference between these two AUC values was used as a diagnostic metric to assess how much performance deteriorated when the model was applied to the test data. Our analyses indicated a moderate degree of overfitting, with the mean relative difference in AUC of approximately 9 ± 10% for the bathymetric model and 7 ± 7% for the latitudinal model.

Because our objective was purely correlative rather than causal, and because we do not aim to predict the bathymetric and latitudinal shifts in other areas (i.e. unseen data for *generalization*), overfitting was less problematic, provided it remained moderate. We therefore conducted additional sensitivity analyses to assess how sensitive the importance values of all predictors (i.e., the values shown in Fig. 5) were to variable deletion and to variation across iterations (i.e. the 5,000 random subsamples of 75% of the observations). To do this, we relied on the reviewer's subsequent comment ("Conduct sensitivity analyses to assess how changes in predictor variables affect the results") and the sensitivity analysis it suggested. These analyses consisted of fitting models for bathymetric and latitudinal shifts using different subsets of predictors. Specifically, we computed the coefficient of determination between predictor importance values from the full model (i.e. those shown in Fig. 5) and those from reduced models. These analyses showed that predictor importance values, including the direction of their effects, were highly stable across models, with median R^2 values of approximately 0.92 ± 0.16 for the bathymetric model and 0.92 ± 0.20 for the latitudinal model. All these results are now included in the revised Methods section (l. 671-685).

This information is now included in the *Methods* section. Please also note that Figure 5 has been updated as we reran the XGBoost analyses to minimize overfitting and better assess the strength of the predictors' effects. We decided to apply the Fukami et al. approach not only to evaluate the direction of the effects (i.e. what we did in the previous version) but also their magnitude. Consequently, Figure S12 (now Supplementary Fig. 18) and Table S5 (now Supplementary Table 7) were also updated.

Reference:

Fukami, T., Martijn Bezemer, T., Mortimer, S. R. & Putten, W. H. Species divergence and trait convergence in experimental plant community assembly. *Ecol. Lett.* **8**, 1283–1290 (2005).

On the other hand, classical statistical analyses (e.g., Wilcoxon tests) were also used to detect changes in displacement rates, a method suitable for nonparametric comparisons in time-trend data. The description of mean speedsOn the other hand, classical statistical analyses (e.g., Wilcoxon tests) were also used to detect changes in displacement rates, a and confidence intervals for displacements indicates an appropriate use of descriptive and inferential statistics.

Recommendations
1. Validation of machine learning models: Conduct sensitivity analyses to assess how changes in predictor variables affect the results, especially in displacement estimates.

We conducted several sensitivity analyses by selecting different subsets of predictors to model bathymetric and latitudinal shifts. Two metrics were used to quantify the models' sensitivity to predictor removal. First, we calculated the coefficient of determination between predictor importance values from the complete model and those from the reduced models. Second, we measured the proportion of predictors for which the sign of the estimated effect was preserved across models. Both analyses indicated a low sensitivity to predictor omission, particularly with respect to the direction of each predictor's effect on the probability of shifting deeper or northward, as well as the relative importance of predictors (see graphs below). These analyses showed that predictor importance values were relatively stable regardless of the number of selected predictors, with a median R^2 of approximately 0.79 ± 0.15 for the bathymetric model and 0.87 ± 0.10 for the latitudinal model. These new results have now been incorporated into the revised *Methods* section (see l. 676-685 and Supplementary Fig. 28).

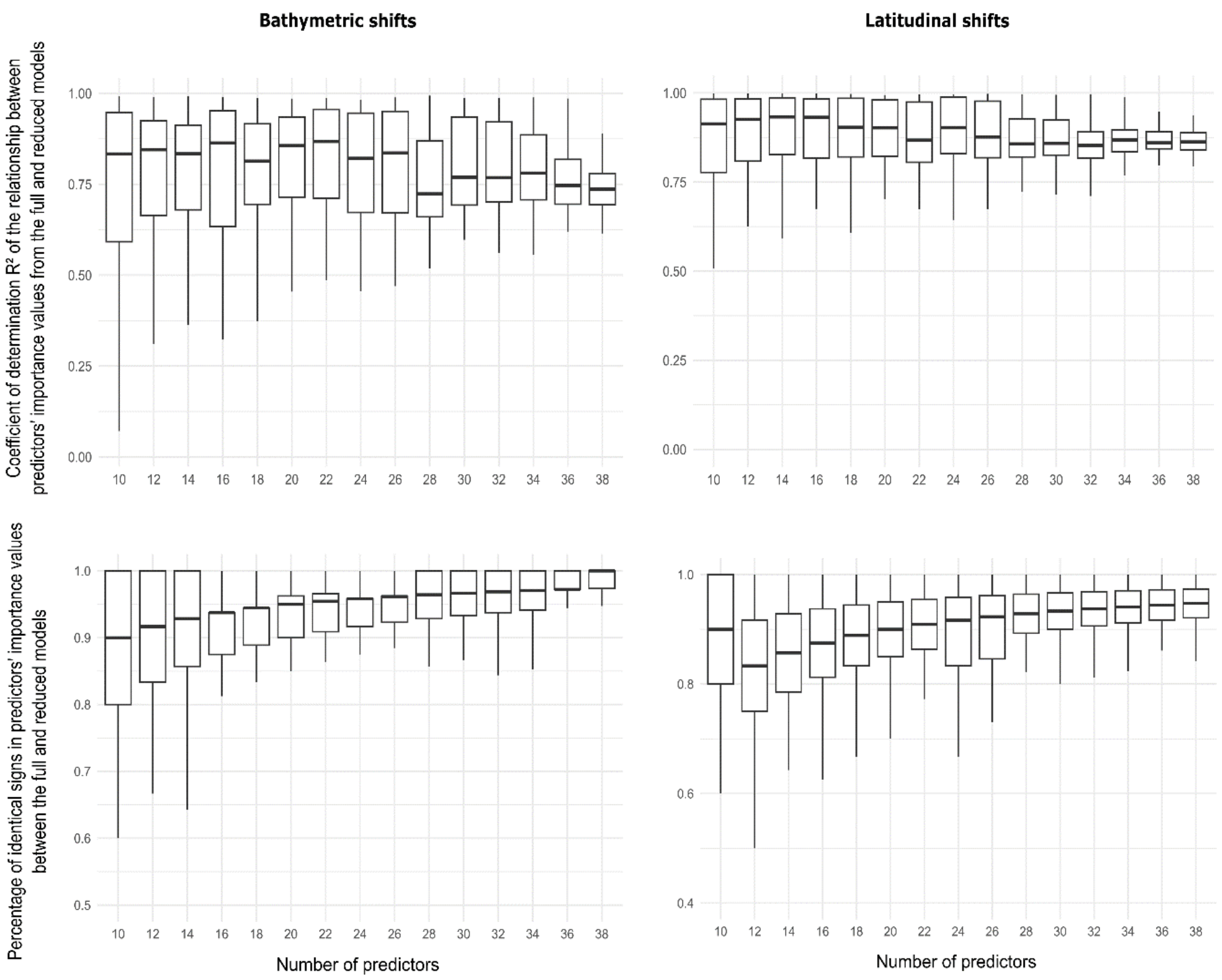

2. Explore traditional complementary methods such as generalized linear models (GLMs) or mixed models to assess the consistency of the trends detected by machine learning, especially in temporal and spatial trend analyses.

At the start of our project, we initially deployed a GLM for our modeling framework; however, the high number of predictors inevitably required reducing the model size. In this study, it was important for us to adopt an integrative approach by incorporating all predictors (i.e. species traits, environmental safety margins, environmental trends, and fishing). Additionally, we aimed to follow a qualitative approach by estimating the direction of each predictor's effect on the probability of a species shifting deeper or northward. To assess this direction, we applied the approach of Fukami et al. (2005), which compares model predictions to null models. Hence, to provide the most accurate assessment of the effect of 100% of the predictors, we needed a method that handles several aspects, particularly missing data and overparametrization-induced bias in coefficient estimates.

Among other reasons, this requirement (i.e. keeping all predictors to provide information rather than excluding them due to model reduction) was the main reason we did not use GLMs.

In addition, GLMs are more sensitive to outliers, which may lead to biased estimated coefficients. Our dataset also contained missing values (e.g. trait values unavailable for particular species; in our case, ~8% of the dataset was NA). XGBoost automatically learns the optimal path for observations with missing data within each tree, eliminating the need for prior imputation or the complex creation of missing indicators required by GLMs, which can lead to unstable coefficient estimates. Therefore, we had to choose a more sophisticated modeling approach that does not require discarding observations or imputing missing data.

Overparameterization is another issue for GLMs: as parametric models, they require the estimation of every coefficient, leading to high variance and overfitting as the number of predictors grows. In contrast, although overparameterization can occur with XGBoost (and often does), it is not so problematic because XGBoost is non-parametric and automatically selects informative variables at each split, using only a subset of features. As a result, the importance assigned to each predictor remains unchanged, even when all predictors are included.

In summary, while a more straightforward approach, such as a GLM may be informative, given our primary aim to consider all predictors and the reasons listed above, we prioritized the use of a machine learning method.

Discussion
Although they are mentioned in the references section, developing a statement using three-dimensional species distribution models, given that depth movements are predominant, it is essential to include the vertical dimension (depth) in marine conservation prediction and planning models to better capture the spatial dynamics of species.

In addition to the sentence "*This diversity, in turn, provides potential refuges for metapopulations experiencing changing conditions, underscoring the importance of prioritizing three-dimensional species distribution models when projecting the distribution of marine fishes under climate change scenarios. Consequently, including the bathymetric dimension in marine conservation prediction and planning models appears essential for more accurately capturing the spatial dynamics of species (Kitchel et al., 2022; Owens et al., 2025).*", we have now explicitly added that incorporating the bathymetric dimension into marine conservation prediction and planning models will be a key step to better capture the spatial dynamics of species (see l. 278-280). In this context, we also now refer to two recent papers :

Owens, H. L. & Rahbek, C. Depth Matters for Marine Biodiversity. *J. Biogeogr.* e70080 (2025) doi:10.1111/jbi.70080.

Kitchel, Z. J., Conrad, H. M., Selden, R. L. & Pinsky, M. L. The role of continental shelf bathymetry in shaping marine range shifts in the face of climate change. *Glob. Change Biol.* 28, 5185–5199 (2022).

Explicitly mention or discuss that understanding changes in species distribution requires long-term, standardized, and large-scale data. It is crucial to maintain and expand these monitoring programs to detect emerging changes and evaluate the effectiveness of implemented policies.

We thank the reviewer for this relevant point. In relation with a previous comment, we have now explicitly mentioned this in the discussion section (see l. 371-374) : "*Harmonizing scientific survey protocols and extending their temporal coverage then appear to be key steps in minimizing biases from protocol heterogeneity, particularly when aiming to detect emerging changes and assess the effectiveness of implemented policies at both local and large spatial scales.*"

Discuss the prioritization of refuge areas in environments with high habitat diversity. Bathymetric heterogeneity provides refuges for metapopulations, so protecting areas with diverse environments and depths can strengthen the resilience of marine communities to climate change.

We have now mentioned this in the discussion section (l. 280-282): "*Furthermore, because bathymetric heterogeneity may offer refuges for fish populations (Koivunen et al., 2025), protecting areas with diverse environments and depths can enhance the resilience of marine communities to climate change.*"

Language

No errors were observed in the grammatical structure, only some punctuation details and the elimination of text containing redundancies or repeated ideas. Most of the redundancies are related to the deepening trend in species movements and the role of bathymetry. The reiteration that movements toward greater depths predominate, and that these movements also occur in northerly and southerly directions, appears several times, reinforcing the general trend, but which can be considered redundant.

We agree that some of these redundancies are unnecessary. We've now identified and removed them.

Figures

The figures are appropriately related to the content of the text and the data presented. The figures effectively represent the study's findings.

We thank the reviewer for this positive feedback.

References

The bibliographic references are adequately cited. According to the reference list, they include publications ranging from 2003 to 2024, indicating recent research. Some references from 2023 and 2024 reflect updated literature on climate change and marine species distribution.

We thank the reviewer for this positive feedback.

Minor comments:

Line 21. Body of research by "Body of evidence"

This is now corrected.

Line 31. How does it increase the biodiversity metric?

We have now included information indicating that poleward shifts can increase certain biodiversity metrics (e.g. species richness and beta diversity; Gordó-Vilaseca et al., 2023).

Line 53: I suggest replacing "spatial pixels" (image element) with "grid cell" (discrete unit within a grid).

This is now corrected.

Line 58. Center of gravity or Centroid of geographic distribution?

Thank you for this suggestion. "Centroid of geographic distribution" is more appropriate, and we have now corrected it accordingly.

Line 79

"Because the latitudinal range of each metapopulation within a given survey may therefore represent only a fraction of its full global distribution, our ability to detect latitudinal shifts could be limited." The sentence is correct, although it sounds a bit long. You can improve the punctuation if you wish, but it's not mandatory.

We have now improved and shortened the sentence as follows:

"*Because the latitudinal range of each population may represent only a fraction of its overall global distribution (median = 33%; Supplementary Fig. 10), our ability to detect latitudinal shifts may be limited*". (see l. 459-461).

Line 100: "More specifically, this spatial expansion of metapopulations has mainly been associated with movements toward greater depths rather than toward the surface, as well as toward the north or south". The mention of movements towards greater depths and in north and south directions is repeated in different parts of the analysis, reinforcing the trend without providing additional substantive information.

We thank the reviewer for this comment, which prompted us to correct the sentence. This key statement explains that, among expanding species, a higher percentage shifted to deeper rather than shallower areas, while northward and southward shifts occurred in similar proportions (Fig. 4b). Together with a comment from another reviewer, we have now revised the sentence as follows: *"More specifically, the spatial expansion of populations was primarily associated with geographic shifts toward deeper rather than shallower areas, whereas northward and southward expansions were proportionally similar*" (see l. 167-170).

Line 122. Both species???

We were referring to species with significant temporal trends and those with non-significant trends. The sentence has now been clarified as follows: *"All species are shown, whether they exhibit a significant or a non-significant temporal change in depth and latitudinal position of their center of gravity"* (l. 153-154).

Line 140. "NS" is not necessary.

This is now removed.

Line 161. I suggest: extreme gradient boosting (XGBoost)-based machine learning method.

This is now corrected.

Line 208:“Using an extensive dataset from 21 bottom trawl surveys conducted across European continental shelf waters over the last four decades, we identify a strong discrepancy between depth and latitudinal shifts in marine fish species.” In other sections, it is repeated that deep displacements are more frequent or significant, which can be considered a reiteration.

In the previous version of the manuscript, the section title ‘Discussion’ was not displayed. As this sentence is the first of the Discussion section, it simply summarizes the main findings of our work. We have now included the ‘Results’ and ‘Discussion’ section titles to avoid repetition.

Line 217. Geographic barrier, like which one(s)?

We have now included examples from the south coast of England, while the northern Mediterranean Sea example is presented in the following sentence (l.271 and 330).

Line 217: The idea of species moving deeper and its relationship to environmental change is repeated several times in the same paragraph and throughout the document, reinforcing the trend without providing any new information.

In relation to the earlier comment about repetition, we have now added the section title “Discussion,” which makes such repetitions more acceptable. Additionally, to reduce redundancy, we have rephrased the sentence (originally on line 217). The repetition is now mitigated by removing the clause “*...adapting by moving to greater depths or…*”

Line 219 “The mentions the “deepening” trend in several places and explains again the diversity of environments created by bathymetry, reiterating the same idea redundantly.

In relation to a previous comment, the section title ‘Discussion’ was not displayed. As this sentence is the first of the Discussion section, it simply summarizes the main findings of our work. We have now included the ‘Results’ and ‘Discussion’ section titles to avoid repetition.

Line 257. Replace “geographical niche” by ecological niche.

This is now corrected.

Line 264. The idea that these environmental mosaics are “critical” is repeated in several parts of the text, highlighting that the diversity of environmental conditions and their direction are key requirements for understanding spatial movements.

This repetition is now deleted by replacing ‘critical’ by ‘key’ (l. 337-339).

Line 273. Use the concept of ‘ecological niche conservatism’ in this section.

Thank you again. This has now been included (l. 346-347).

Lines 287 to 290: Sentence repeated with paragraphs 273 to 276.

We thank you for pointing this out, which helped us to clarify the text. The sentence of the last paragraph is now removed and its homologue sentence of the previous paragraph (i.e. l.273-273 of the initial version) is now reworded as follows: "*Therefore, further development of advanced numerical approaches that incorporate local hydrography, natural climate oscillations, and biotic interactions should be a priority for this field of research.*" (see l. 350-352). We have also reworded the final paragraph to highlight the utility of safety margin traits better.

Line 289: Delete "more" and "future." Mitigation is now!

This is now removed.

Line 509: Incomplete citation: McFadden, D. (1973) Conditional Logit Analysis of Qualitative Choice Behavior. In: Zarembka, P., Ed., Frontiers in Econometrics, Academic Press, 105-142.

Thank you very much. This is now corrected.

Line 563: References 7 and 31 do not mention center of gravity, only center of abundance. Please clarify.

In relation to a previous comment, we have now replaced the centre of gravity by "Centroid of geographic distribution" (CGD).

Line 630: There is no information on variable correlation analysis (variable selection), but there is a figure in the supplementary.

In this study, we aimed to follow an integrative approach by incorporating all predictors, which explains why this supplementary figure is provided solely to illustrate the relationships among environmental variables.

Line 656: Replace bioecological with "ecological".

This is now corrected.

Line 692: Bio-Oracle version?

Version 22 was downloaded for all environmental parameters, except for depth, which corresponds to version 10. This has now been included in the revised manuscript (new Supplementary Table 11).

Line 721: Extreme gradient boosting (XGBoost)-based machine learning method

This has now been corrected.

Line 722: XGBoost algorithm, Line 726 XGBoost model, standardize method name

This has now been standardized with 'XGBoost model'.

Line 730: Bottom O_2, or Dissolved Oxygen?

Thank you for catching that. It refers to dissolved oxygen. This has now been corrected.

Fig 5. line 199: O2 tolerance or Dissolved Oxygen?
This is dissolved oxygen. This has now been corrected.

Remarks on code availability:
The files and codes are complete and fully functional.

We thank the reviewer for this positive feedback.

RESPONSE TO REFEREES

NCOMMS-25-49068A

Title: Pervasive deepening-driven range expansion of marine fish populations in a sea of change

Proposition of new title : Marine fish ranges are expanding and shifting deeper across the European continental shelf

We are grateful to the referees for their time and effort in evaluating our manuscript.

The revised manuscript is provided with tracked changes. Reviewers' comments are shown in **black**, and our responses are shown in **blue**.

Reviewer #1 (Remarks to the Author):

Firstly I would like to thank the authors for their detailed and thorough responses to all of the reviewer comments submitted at the last review.

All of my main concerns surrounding the manuscript and general approach have been addressed, often through additional and comprehensive statistical analyses.

I particularly like the approach taken to assess whether species having a distributional range larger than the survey areas impacted the ability to detect range shifts (lines 123 - 131). I was also glad to see that the removal of pelagic species did not cause large shifts in the results. One consideration is whether this needs to be in the main text, or the results for demersal species minus pelagics are presented, with the methods detailing that pelagics were removed. I think either way works, but that is an option to reduce some of the results text.
We thank the reviewer for this suggestion. Although these additional results (i.e., the effect of including or excluding pelagic species) slightly increase the length of the paper, we believe that presenting results for all species (both demersal and pelagic) is preferable to focusing solely on demersal species, as it provides more complete information about the fish community. However, we acknowledge that greater caution is necessary when interpreting results for pelagic species, which we addressed thanks to the previous round of review.

One further question is regarding the inclusion of some of the trawl data - on lines 392 - 394, you now show the spread of speed and duration for all hauls. Some of these seem both very slow (i.e. is the gear fishing properly < 1 kt?, depending on the gear, around 3 is ideal) and very fast (i.e. how did they manage to successfully deploy gear from a vessel moving 18 kts?). Equally, for short duration hauls - was this 5 minutes of bottom time? (I assume so) if not, this also may not have fished properly. It may be worth looking at the catch data for some of those outliers and removing them. You could even have some inclusion thresholds based on other trawl-focussed studies.

We thank the reviewer for this comment. Although speed values did not influence our calculations, we decided, as a precaution, to remove these “extreme” hauls from our analyses, as recommended. To prevent the risk of including non-representative catch data, we re-ran most of the analyses after excluding hauls with extreme speed values: within each survey, we retained only hauls with speeds between the 5th and 95th percentiles. We also removed hauls with a trawling duration of less than 10 minutes. Here is the added/modified text in the Methods section (see yellow highlights in Methods section) :

To prevent the inclusion of non-representative catch data, hauls associated with excessively low or high trawling speeds were excluded. For each survey, only hauls with speeds between the 5th and 95th percentiles were retained. Additionally, hauls with a trawling duration of less than 10 minutes were removed to ensure consistency in catch data. Sampling was conducted during daylight hours at an average speed of 3.4 ± 0.5 knots (min: 2.3; 1st decile: 2.8; median: 3.2; last decile: 4.1; max: 5.4) and an average duration of 34.4 ± 13.2 minutes (min: 10; 1st decile: 22; median: 30; last decile: 60; max: 90).

Overall, this procedure reduced the dataset by approximately 13%. We then re-ran most of analyses presented in the manuscript and supplementary informations, which did not change our results or conclusions. All results are now updated.

I have only a couple of minor edits, as follows:

Line 38: remove the second 'h' from shifting
This now corrected.

Line 83: what are the SD/SE values for ave speed and age duration? I know more detailed values are provided in the methods, but some accounting of variability would be nice to have here as well.
Standard deviation values are now included.

Line 334: change 'limit' to 'limits'
This is now corrected.

Reviewer #1 (Remarks on code availability):
n/a

Reviewer #4 (Remarks to the Author):

Dear Authors,
I have completed my review of the manuscript. I am pleased to see that all of my previous observations have been fully addressed. Furthermore, the revisions made by the other reviewers have significantly enhanced the quality of this research. At this stage, I have no further comments.
We thank the reviewer for this positive evaluation of our previous revisions.

Reviewer #4 (Remarks on code availability):
I have reviewed the supplementary material, finding the code and the results obtained in extenso.
