# Supplementary material for: Marine fish ranges are expanding and shifting deeper across the European continental shelf
Source: Nat Commun. 2026 Jul 15;17:8702. doi: 10.1038/s41467-026-74371-8 (PMC13490448; doi:10.1038/s41467-026-74371-8)
Supplement: Supplementary file 4 [file 41467_2026_74371_MOESM2_ESM.docx]

Supplementary Data 1. **Studies exploring latitudinal shifts in marine species and their relation to climate change**. Yellow shading depicts studies showing an overall poleward shift in abundance, blue the opposite, and white depicts equivocal shifts (e.g., poleward and equatorward).

Supplementary Data 2. **Studies exploring depth shifts in marine species and their relation to climate change**. Red shading depicts studies showing an overall deepening shift in abundance, blue the opposite, and white depicts equivocal shifts (e.g., deepening and shoaling).
