# Supplementary material for: Marine fish ranges are expanding and shifting deeper across the European continental shelf
Source: Nat Commun. 2026 Jul 15;17:8702. doi: 10.1038/s41467-026-74371-8 (PMC13490448; doi:10.1038/s41467-026-74371-8)
Supplement: Supplementary file 5 [file 41467_2026_74371_MOESM3_ESM.docx]

| **N** | **Reference** | **Geographic Focus** | **Length of Study** | **Species Examined** | **Latitudinal and bathymetric shift** |  |
| --- | --- | --- | --- | --- | --- | --- |
| 1. | Palacios-Abrantes, J. *et al.* Quantifying fish range shifts across poorly defined management boundaries. *PLoS ONE* 18, e0279025 (2023). | U.S. Northeast Atlantic Coast, Exclusive Economic Zone | 1951-2019 | Black sea bass, summer flounder, scup | Black sea bass shifted 3° north in the fall since 1980, with scup shifting 2° north in both seasons. Summer flounder shifted north by 1° in both seasons, with some eastward movement in the spring. |  |
| 2. | Astthorsson, O. S., Valdimarsson, H., Gudmundsdottir, A. & Óskarsson, G. J. Climate-related variations in the occurrence and distribution of mackerel (Scomber scombrus) in Icelandic waters. *ICES Journal of Marine Science* 69, 1289–1297 (2012). | Icelandic waters | 1883–2010 | Atlantic mackerel (*Scomber scombrus*) | Mackerel exhibited a significant northward shift during warm periods, extending up to 400 miles west and 120 miles north into Icelandic waters. Most of the mackerel population moved northward, especially during warm phases of the Atlantic Multidecadal Oscillation (AMO). The rate of this latitudinal shift became especially noticeable after 2007. |  |
| 3. | Bradbury, I. R. *et al.* Parallel adaptive evolution of Atlantic cod on both sides of the Atlantic Ocean in response to temperature. *Proc. R. Soc. B.* 277, 3725–3734 (2010). | North Atlantic (Eastern and Western sides) | 1996–2007 | Atlantic cod (*Gadus morhua*) | The study found significant latitudinal shifts in allele frequencies along both the eastern and western Atlantic, correlated with ocean temperature. Cod populations showed a strong temperature-associated clinal differentiation. The northward movement of populations was associated with warming waters, though no specific rate of movement was detailed. |  |
| 4. | Cheung, W. W. L., Watson, R. & Pauly, D. Signature of ocean warming in global fisheries catch. *Nature* 497, 365–368 (2013). | Global; 52 large marine ecosystems (LMEs), including tropical, temperate, and polar regions. | 1970–2006 | Marine fish and invertebrates (990 species) | Species are shifting toward higher latitudes (northward in the northern hemisphere, southward in the southern hemisphere). The rate of latitudinal shift in non-tropical LMEs is 0.23°C per decade, with noticeable poleward shifts in warmer-water species becoming dominant in higher-latitude catches. In tropical regions, the rate of change was initially 0.19°C per decade but stabilized. |  |
| 5. | Crear, D. P., Curtis, T. H., Hutt, C. P. & Lee, Y. Climate‐influenced shifts in a highly migratory species recreational fishery. *Fisheries Oceanography* 32, 327–340 (2023). | U.S. Northeast Shelf, from Maine to Virginia | 2002–2019 | 12 HMS groups: tunas, sharks, billfishes | All species except white marlin showed significant poleward shifts, with latitudinal shifts ranging from 1 to 10 km per year, driven by increasing SST. Notably, small bluefin tuna showed a 10 km/year shift, and blue sharks shifted 40 km/°C. |  |
| 6. | Fredston, A. *et al.* Range edges of North American marine species are tracking temperature over decades. *Global Change Biology* 27, 3145–3156 (2021). | Northeast U.S., U.S. West Coast, Eastern Bering Sea | 1968–2018 (varies by region) | 165 species of marine fish and invertebrates | Most species shifted poleward, with average shifts of 4.4 km/year in the Northeast U.S., 1.3 km/year on the U.S. West Coast, and minimal shifts (0.1 km/year) in the Eastern Bering Sea. A few species, such as American lobster, shifted faster, up to 24.5 km/year equatorward. 88% of species tracked thermal niches. |  |
| 7. | Hazen, E. L. *et al.* Predicted habitat shifts of Pacific top predators in a changing climate. *Nature Clim Change* 3, 234–238 (2013). | North Pacific, including the California Current and North Pacific Transition Zone | 2001–2010, with projections to 2100 | 15 species, including sharks, tunas, seabirds, and marine mammals | Predicted significant northward shifts in habitat for many species, with up to 1,000 km northward migration in the North Pacific Transition Zone. The rate of northward shift varied among species, with species such as tuna and sharks experiencing the largest shifts. Species with specialized habitats, like blue whales, experienced habitat losses, while generalists, such as seabirds, gained habitat. |  |
| 8. | Hollowed, A. B. & Sundby, S. Change is coming to the northern oceans. *Science* 344, 1084–1085 (2014). | North Pacific and North Atlantic Oceans | Early 20th century to 2013 | Northeast Arctic cod (*Gadus morhua*), Bering Sea pollock (*Gadus chalcogrammus*) | Both cod and pollock showed significant northward shifts. Cod moved toward the Arctic front, reaching new northern limits, while pollock expanded northward during warm periods. In recent decades, cod experienced a poleward displacement during warmer phases, while pollock remained confined to warmer southern waters. |  |
| 9. | ICES. Report of the Working Group on Fish Distribution Shifts (WKFISHDISH). (2016) | Northeast Atlantic (various ICES regions, including the North Sea, Bay of Biscay, Celtic Sea, and Barents Sea) | 1970s–2016 | 19 species, including cod, anchovy, blue whiting, mackerel, haddock, herring | Many species exhibited poleward shifts, especially in response to warming waters. For example, anchovy and mackerel exhibited significant northward shifts. Cod also displayed a northward shift in the Barents Sea. The rate of movement varied, with some species moving up to 10-70 km/°C. |  |
| 10. | Jacobsen, N. S., Marshall, K. N., Berger, A. M., Grandin, C. & Taylor, I. G. Climate-mediated stock redistribution causes increased risk and challenges for fisheries management. *ICES Journal of Marine Science* 79, 1120–1132 (2022). | North Pacific, specifically U.S. and Canadian waters of the California Current | 1965–2048 (projected scenarios) | Pacific hake (*Merluccius productus*) | The study highlighted significant northward shifts for Pacific hake, with the rate of movement linked to sea temperatures. Increased northward movement was observed during warmer years, with up to 80% of the stock in Canadian waters in some scenarios. These movements intensified over time, with a projected northward shift of ~2-4 km per year, depending on the scenario. |  |
| 11. | Le Luherne, E., Pawlowski, L. & Robert, M. Northeast Atlantic species distribution shifts over the last two decades. *Global Change Biology* 30, e17383 (2024). | Celtic-Biscay Shelf, Bay of Biscay, and Celtic Sea | 1997–2020 | 93 taxa, including fish (65 Actinopteri), cephalopods (11), and bivalves (2) | 56% of taxa shifted latitudinally, with the majority moving northward (mean rate: 65 km/decade). Lusitanian species displayed significant northward shifts, while a smaller portion of demersal species shifted southward. |  |
| 12. | MacKenzie, B. R., Payne, M. R., Boje, J., Høyer, J. L. & Siegstad, H. A cascade of warming impacts brings bluefin tuna to Greenland waters. *Global Change Biology* 20, 2484–2491 (2014). | East Greenland, Denmark Strait, Irminger Sea | Aug-12 | Atlantic bluefin tuna (*Thunnus thynnus*), Atlantic mackerel (*Scomber scombrus*) | Bluefin tuna were observed moving northward beyond their usual range into East Greenland waters, following mackerel and responding to warming waters. The area of suitable habitat for bluefin tuna (SST > 11°C) increased by 720,000 km² between 1985–1994 and 2007–2012. |  |
| 13. | Morita, K., Fukuwaka, M., Tanimata, N. & Yamamura, O. Size-dependent thermal preferences in a pelagic fish. *Oikos* 119, 1265–1272 (2010). | North Pacific Ocean (41°–49°N) | April – May 2006 | Pacific salmon (*Oncorhynchus* spp.) | A northward latitudinal shift was observed with larger salmon found in cooler, northern waters. The average fork length and ocean age of salmon increased with latitude. This latitudinal cline was significant for species like sockeye, chum, and pink salmon, reflecting their movement into colder northern areas as temperatures increased. |  |
| 14. | Bandara, R. M. W. J., Curchitser, E. & Pinsky, M. L. The importance of oxygen for explaining rapid shifts in a marine fish. Glob. Change Biol. 30, e17008 (2024). | Northeast U.S. Continental Shelf, Mid-Atlantic and Gulf of Maine | 1982–2010 | Black Sea Bass (*Centropristis striata*) | Black Sea Bass exhibited a northward shift, moving approximately 80 km/decade over the last five decades. This shift was linked to ocean warming, with the species expanding its range into the Gulf of Maine. The northward movement was consistent with rising temperatures and changes in oxygen availability. |  |
| 15. | Mueter, F. J. & Litzow, M. A. Sea ice retreat alters the biogeography of the bering sea continental shelf. *Ecological Applications* 18, 309–320 (2008). | Southeastern Bering Sea | 1982–2006 | 46 demersal fish and invertebrate species, including snow crab, Arctic cod, and Greenland halibut | Most species exhibited significant northward shifts, with the community-wide center of distribution shifting northward by an average of 34 km. Some species showed shifts up to 230 km north, driven by warming temperatures and retreating sea ice. |  |
| 16. | Nye, J., Link, J., Hare, J. & Overholtz, W. Changing spatial distribution of fish stocks in relation to climate and population size on the Northeast United States continental shelf. *Mar. Ecol. Prog. Ser.* 393, 111–129 (2009). | Northeast U.S. Continental Shelf (from Gulf of Maine to Mid-Atlantic Bight) | 1968–2007 | 36 fish stocks, including Atlantic cod, silver hake, yellowtail flounder, and haddock | 17 species exhibited significant poleward shifts, particularly southern stocks. Yellowtail flounder and American shad moved northward at an average rate of 1-8 km/year. Some species, such as Atlantic cod, showed range contractions. |  |
| 17. | Panzeri, D. *et al.* Future distribution of demersal species in a warming Mediterranean sub-basin. *Front. Mar. Sci.* 11, 1308325 (2024). | Adriatic and Western Ionian Seas (Mediterranean sub-basins) | 1999-2018 (observational) with projections to 2035 and 2050 | Nine demersal species | Many species, such as European hake and red mullet, show significant northward shifts, with migration rates increasing under warming conditions. The latitudinal shift is more pronounced in the central and northern Adriatic, where thermophilic species are moving northwards by up to 65 km/decade. Cold-water species face distribution losses in southern areas. |  |
|  |  |  |  |  |  |  |
| 18. | Perry, A. L., Low, P. J., Ellis, J. R. & Reynolds, J. D. Climate Change and Distribution Shifts in Marine Fishes. *Science* 308, 1912–1915 (2005). | North Sea | 1978-2001 | 36 species of demersal fishes. | Northward shifts for 13 of the 15 species exhibited significant latitudinal shifts. |  |
| 19. | Walsh, H. J., Richardson, D. E., Marancik, K. E. & Hare, J. A. Long-Term Changes in the Distributions of Larval and Adult Fish in the Northeast U.S. Shelf Ecosystem. *PLoS ONE* 10, e0137382 (2015). | Northeast U.S. Shelf Ecosystem | 1977-1987 and 1999-2008 | 58 taxa of larval and adult fish including Atlantic cod, haddock, red hake, yellowtail flounder | 50% of adult taxa exhibited latitudinal shifts, with 23 taxa moving predominantly northward. For example, Atlantic cod larvae shifted northward along with a significant proportion of other species such as yellowtail flounder and red hake. Adult Atlantic cod and yellowtail flounder also moved northward, especially in spring. Some species, such as spiny dogfish, moved southward. Shifts were generally consistent with warming sea temperatures, with rates of northward movement ranging from moderate to significant depending on species and region. |  |
|  |  |  |  |  |  |  |
| 20 | Gordó-Vilaseca, C. et al. Over 20% of marine fishes shifting in the North and Barents Seas, but not in the Norwegian Sea. PeerJ 11, e15801 (2023). | North Sea, Norwegian Sea, Barents Sea | 1998-2020 | Norwegian and Barents Sea: 147 marine fish species (classes Actinopterygii, Elasmobranchii, Holocephali, Myxini and Petromyzonti). North Sea : 29 marine fish species from GOV. | Overall significant northward shift of the marine fish community biomass in the North Sea, and individual species northward shifts in the Barents and North Seas, in 20% and 25% of the species’ biomass centroids in each respective region. No overall community shifts in the Norwegian Sea. When each species was assessed individually, 15 species of 58 showed clear northward shifts of their biomass centroid in the North Sea (26%), while two species showed a clear southward shift (4%). In the Norwegian Sea, two species were detected to shift northwards and two species southwards of a total of 26 species (8%), and seven species of 35 showed clear northward shifts in the Barents Sea (20%), while two species showed a clear southward shift (6%). |  |
| 21 | Dulvy, N. K. *et al.* Climate change and deepening of the North Sea fish assemblage: a biotic indicator of warming seas. *Journal of Applied Ecology* 45, 1029–1039 (2008). | North Sea | 1980–2004 | 28 species of demersal fish | Latitudinal shifts were heterogeneous, with widespread, abundant, warm-tolerant species (like grey gurnard) moving northward. However, some small, warm-tolerant southern species (like scaldfish and solenette) shifted southward, driven by warmer winter temperatures. The rate of northward shift for some species was 10-70 km/°C, while minimum latitudes for southern species shifted southward by up to 80 km/°C. |  |
| 22 | Keller, A. A., Bradburn, M. J. & Simon, V. H. Shifts in condition and distribution of eastern North Pacific flatfish along the U.S. west coast (2003–2010). *Deep Sea Research Part I: Oceanographic Research Papers* 77, 23–35 (2013). | U.S. West Coast, California Current System | 2003–2010 | 10 flatfish species, including arrowtooth flounder, Dover sole, Pacific sanddab | Species like Dover sole, deepsea sole, and rex sole shifted southward during cooler PDO phases and northward during warmer periods. Average latitudinal displacements ranged from 22 km to over 96 km, depending on the species and environmental conditions. |  |
| 23 | Lezama‐Ochoa, N. et al. Divergent responses of highly migratory species to climate change in the California Current. Divers. Distrib. 30, e13800 (2024). | California Current System | 1980–2100 (projected) | 10 highly migratory species, including blue shark, swordfish, leatherback sea turtle, California sea lion | Four species (blue shark, swordfish, shortfin mako, leatherback turtle) are projected to shift poleward (northward) by more than 100 km, while other species like thresher shark and California sea lion are expected to shift southward or towards the coast. The rate of northward shifts for swordfish and blue sharks is up to 198 km by 2100, while southern shifts are projected to be less than 100 km. |  |
|  |  |  |  |  |  |  |
| 24 | Pinsky, M. L., Worm, B., Fogarty, M. J., Sarmiento, J. L. & Levin, S. A. Marine Taxa Track Local Climate Velocities. *Science* 341, 1239–1242 (2013). | Coastal North America | 1968–2011 | 360 marine species (fish and invertebrates) | 74% of species shifted latitudinally in the same direction as climate velocity. Approximately 54% shifted northward, while 46% shifted southward. The rate of northward shift was variable, with some species moving >11 km/decade in regions like the Gulf of Alaska, where cooler periods caused shifts southward. The majority of shifts followed warming temperatures, but the study notes that not all species exhibited the same pattern due to regional temperature and climate velocity differences. |  |
|  |  |  |  |  |  |  |
| 25 | Ruiz-Diaz, R., Pennino, M. G., Fisher, J. A. D. & Eddy, T. D. Decadal changes in biomass and distribution of key fisheries species on Newfoundland’s Grand Banks. *PLoS ONE* 19, e0300311 (2024). | The Grand Banks, Newfoundland, Canada | 1996-2019 | Snow crab, yellowtail flounder, Atlantic cod | Snow crab: Slight northwestern shift in center of gravity. Yellowtail flounder: Center of gravity remained relatively stable. Atlantic cod: Significant northward shift toward the end of the study, potentially due to stock recovery in the 2J3KL area or a response to regional warming. |  |
| 26. | Atkinson, D. B., Rose, G. A., Murphy, E. F. & Bishop, C. A. Distribution changes and abundance of northern cod (Gadus morhua), 1981–1993. 54, (1997). | Newfoundland, NAFO divisions 2J, 3K, and 3L | 1981–1993 | Northern cod (*Gadus morhua*) | After 1987, the cod shifted southward. This shift intensified after 1989, as the stock contracted into its southern range, likely due to environmental factors and increased fishing pressure. |  |
|  |  |  |  |  |  |  |
