# Supplementary material for: Marine fish ranges are expanding and shifting deeper across the European continental shelf
Source: Nat Commun. 2026 Jul 15;17:8702. doi: 10.1038/s41467-026-74371-8 (PMC13490448; doi:10.1038/s41467-026-74371-8)
Supplement: Supplementary file 6 [file 41467_2026_74371_MOESM4_ESM.docx]

| **N** | **Reference** | **Geographic Focus** | **Length of Study** | **Species Examined** | **Bathymetric shift** |  |
| --- | --- | --- | --- | --- | --- | --- |
| 1. | Atkinson, D. B., Rose, G. A., Murphy, E. F. & Bishop, C. A. Distribution changes and abundance of northern cod (Gadus morhua), 1981–1993. 54, (1997). | Newfoundland, NAFO divisions 2J, 3K, and 3L | 1981–1993 | Northern cod (*Gadus morhua*) | Cod shifted into deeper waters after 1987, correlating with stock decline and colder temperatures. The depth shift was part of a general offshore movement to deeper waters. |  |
| 2. | Cheung, W. W. L., Watson, R. & Pauly, D. Signature of ocean warming in global fisheries catch. *Nature* 497, 365–368 (2013). | Global; 52 large marine ecosystems, including tropical, temperate, and polar regions. | 1970–2006 | Marine fish and invertebrates (990 species) | Species shift to deeper waters as oceans warm, especially in non-tropical regions. The rate of depth shift is not explicitly given but is inferred through the increasing MTC. |  |
| 3. | Dulvy, N. K. *et al.* Climate change and deepening of the North Sea fish assemblage: a biotic indicator of warming seas. *Journal of Applied Ecology* 45, 1029–1039 (2008). | North Sea | 1980–2004 | 28 species of demersal fish | The study found a significant deepening of fish assemblages at an average rate of ~3.6 meters per decade. Coldwater species deepened more rapidly (up to 14 m/decade), while warmwater species, such as sole and bib, showed shallowing trends (up to 7.6 m/decade). |  |
| 4. | Hollowed, A. B. & Sundby, S. Change is coming to the northern oceans. *Science* 344, 1084–1085 (2014). | North Pacific and North Atlantic Oceans, particularly the Barents Sea and Bering Sea | Early 20th century to 2013 | Northeast Arctic cod (*Gadus morhua*), Bering Sea pollock (*Gadus chalcogrammus*) | Cod stocks were observed to move into deeper waters as temperatures increased, particularly during warm phases. Pollock distribution was also influenced by cold bottom waters in the Bering Sea, but less is noted about depth shifts. |  |
| 5. | ICES. Report of the Working Group on Fish Distribution Shifts (WKFISHDISH). (2016) | Northeast Atlantic (various ICES regions, including the North Sea, Bay of Biscay, Celtic Sea, and Barents Sea) | 1970s–2016 | 19 species, including cod, anchovy, blue whiting, mackerel, haddock, herring | Some species showed significant deepening, particularly as a response to temperature increases. Cod and haddock were observed to shift deeper in certain regions. However, species like plaice and sole showed limited depth shifts, primarily influenced by density-dependent habitat use. |  |
| 6. | Keller, A. A., Bradburn, M. J. & Simon, V. H. Shifts in condition and distribution of eastern North Pacific flatfish along the U.S. west coast (2003–2010). *Deep Sea Research Part I: Oceanographic Research Papers* 77, 23–35 (2013). | U.S. West Coast, California Current System | 2003–2010 | 10 flatfish species, including arrowtooth flounder, Dover sole, Pacific sanddab | 80% of species showed significant depth shifts. Species like Dover sole, arrowtooth flounder, and rex sole moved into deeper waters during cooler phases. Curlfin sole and Pacific sanddab also shifted deeper, with curlfin sole showing a southward and offshore shift. |  |
| 7. | Le Luherne, E., Pawlowski, L. & Robert, M. Northeast Atlantic species distribution shifts over the last two decades. *Global Change Biology* 30, e17383 (2024). | Celtic-Biscay Shelf, Bay of Biscay, and Celtic Sea | 1997–2020 | 93 taxa, including fish (65 Actinopteri), cephalopods (11), and bivalves (2) | 56% of taxa shifted, with significant depth shifts observed, mostly showing deepening (mean rate: 16.5 m/decade). Some species shifted closer to the surface, particularly boreal species. Depth shifts varied by taxa and region, with deeper movements observed in the Bay of Biscay populations. |  |
| 8. | Nye, J., Link, J., Hare, J. & Overholtz, W. Changing spatial distribution of fish stocks in relation to climate and population size on the Northeast United States continental shelf. *Mar. Ecol. Prog. Ser.* 393, 111–129 (2009). | Northeast U.S. Continental Shelf (from Gulf of Maine to Mid-Atlantic Bight) | 1968–2007 | 36 fish stocks, including Atlantic cod, silver hake, yellowtail flounder, and haddock | 17 species showed significant depth shifts, with 8 species also shifting poleward. Examples include haddock and Atlantic cod, which moved into deeper waters, particularly in the Gulf of Maine. Average deepening across species of 7 m/decade. |  |
| 9. | Panzeri, D. *et al.* Future distribution of demersal species in a warming Mediterranean sub-basin. *Front. Mar. Sci.* 11, 1308325 (2024). | Adriatic and Western Ionian Seas (Mediterranean sub-basins) | 1999-2018 (observational) with projections to 2035 and 2050 | Nine demersal species | Significant deepening trends were observed, especially for European hake and red mullet, which move deeper as temperatures increase. Depth shifts for these species are driven by biogeochemical changes in the water column, with deepening projected to continue into 2050. |  |
| 10. | Perry, A. L., Low, P. J., Ellis, J. R. & Reynolds, J. D. Climate Change and Distribution Shifts in Marine Fishes. *Science* 308, 1912–1915 (2005). | North Sea | 1978-2001 | 36 species of demersal fishes. | Most species showed evidence of deepening. |  |
| 11. | Walsh, H. J., Richardson, D. E., Marancik, K. E. & Hare, J. A. Long-Term Changes in the Distributions of Larval and Adult Fish in the Northeast U.S. Shelf Ecosystem. *PLoS ONE* 10, e0137382 (2015). | Northeast U.S. Shelf Ecosystem | 1977-1987 and 1999-2008 | 58 taxa of larval and adult fish including Atlantic cod, haddock, red hake, yellowtail flounder | 43% of larval species exhibited depth shifts. Larval Atlantic cod shifted deeper, particularly on Georges Bank. About 75% of adult species shifted deeper, with red hake and yellowtail flounder showing notable movement into deeper waters. Pollock also shifted deeper in both spring and fall. Rates of depth shifts varied by species and region. |  |
| 12. | Palacios-Abrantes, J. *et al.* Quantifying fish range shifts across poorly defined management boundaries. *PLoS ONE* 18, e0279025 (2023). | U.S. Northeast Atlantic Coast | 1951-2019 | Black sea bass, summer flounder, scup | Black sea bass showed minimal depth shifts, expanding longitudinally (eastward), while scup exhibited both latitudinal and depth shifts. |  |
|  |  |  |  |  |  |  |
| 13. | Morita, K., Fukuwaka, M., Tanimata, N. & Yamamura, O. Size-dependent thermal preferences in a pelagic fish. *Oikos* 119, 1265–1272 (2010). | North Pacific Ocean (41°–49°N) | April – May 2006 | Pacific salmon (*Oncorhynchus* spp.) | Larger salmon were found to inhabit deeper, cooler waters. An ontogenetic shift in temperature preference was observed, with larger individuals selecting colder areas as they grew. The shift was consistent with body size, as smaller fish preferred warmer, shallower waters. |  |
|  |  |  |  |  |  |  |
| 14. | Pinsky, M. L., Worm, B., Fogarty, M. J., Sarmiento, J. L. & Levin, S. A. Marine Taxa Track Local Climate Velocities. *Science* 341, 1239–1242 (2013). | Coastal North America | 1968–2011 | 360 marine species (fish and invertebrates) | 58% of species shifted into shallower waters. The rate of depth shift correlated with surface temperatures but not bottom temperatures. Species tended to move shallower at rates consistent with the local climate velocity, though variation was observed across regions and species. Species in regions with faster climate velocities showed faster depth shifts. |  |
|  |  |  |  |  |  |  |
| 15. | Mueter, F. J. & Litzow, M. A. Sea ice retreat alters the biogeography of the bering sea continental shelf. *Ecological Applications* 18, 309–320 (2008). | Southeastern Bering Sea | 1982–2006 | 46 demersal fish and invertebrate species | Some species showed depth shifts, but the study primarily focused on latitudinal shifts. Demersal taxa showed a shoaling trend, with average depth distributions becoming shallower over time. |  |
|  |  |  |  |  |  |  |
